# Dynamic lateral organization of opioid receptors (kappa, mu_wt_ and mu_N40D_) in the plasma membrane at the nanoscale level

**DOI:** 10.1111/tra.12582

**Published:** 2018-06-21

**Authors:** Maciej K. Rogacki, Ottavia Golfetto, Steven J. Tobin, Tianyi Li, Sunetra Biswas, Raphael Jorand, Huiying Zhang, Vlad Radoi, Yu Ming, Per Svenningsson, Daniel Ganjali, Devin L. Wakefield, Athanasios Sideris, Alexander R. Small, Lars Terenius, Tijana Jovanović‐Talisman, Vladana Vukojević

**Affiliations:** ^1^ Department of Clinical Neuroscience Center for Molecular Medicine, Karolinska Institute Stockholm Sweden; ^2^ Department of Molecular Medicine, Beckman Research Institute, City of Hope Duarte California; ^3^ Department of Mechanical and Aerospace Engineering The Henry Samueli School of Engineering, University of California Irvine California; ^4^ Department of Physics and Astronomy California State Polytechnic University Pomona California; ^5^ Department of Molecular and Cellular Neurosciences The Scripps Research Institute La Jolla California

**Keywords:** dynamic lateral organization, fluorescence correlation spectroscopy, GPCR, nanoscopy, opioid receptor, pair‐correlation photoactivated localization microscopy, super‐resolution

## Abstract

Opioid receptors are important pharmacological targets for the management of numerous medical conditions (eg, severe pain), but they are also the gateway to the development of deleterious side effects (eg, opiate addiction). Opioid receptor signaling cascades are well characterized. However, quantitative information regarding their lateral dynamics and nanoscale organization in the plasma membrane remains limited. Since these dynamic properties are important determinants of receptor function, it is crucial to define them. Herein, the nanoscale lateral dynamics and spatial organization of kappa opioid receptor (KOP), wild type mu opioid receptor (MOP_wt_), and its naturally occurring isoform (MOP_N40D_) were quantitatively characterized using fluorescence correlation spectroscopy and photoactivated localization microscopy. Obtained results, supported by ensemble‐averaged Monte Carlo simulations, indicate that these opioid receptors dynamically partition into different domains. In particular, significant exclusion from GM1 ganglioside‐enriched domains and partial association with cholesterol‐enriched domains was observed. Nanodomain size, receptor population density and the fraction of receptors residing outside of nanodomains were receptor‐specific. KOP‐containing domains were the largest and most densely populated, with the smallest fraction of molecules residing outside of nanodomains. The opposite was true for MOP_N40D_. Moreover, cholesterol depletion dynamically regulated the partitioning of KOP and MOP_wt_, whereas this effect was not observed for MOP_N40D_.

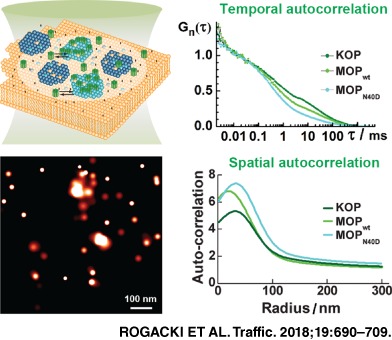

## 1 INTRODUCTION

As proteins and lipids self‐organize in the plasma membrane, cell surface receptors can partition and sort into nanoscale‐sized domains, about 10 to 250 nm in diameter. These domains, also known as lipid rafts, differ from the surrounding lipid bilayer in terms of protein and lipid composition.^1^, ^2^ While the existence of lipid rafts has been debated for many years,^3^ it is now becoming generally accepted that the plasma membrane is laterally organized.^4^ This lateral organization is dynamic, allowing receptors to segregate into domains. To clarify the physiological importance of receptor segregation, a number of details remain to be accurately defined, such as the chemical composition, size and lifetime of receptor‐harboring domains.

Herein, we interrogated receptor‐harboring domains using 2 quantitative approaches with single‐molecule sensitivity: fluorescence correlation spectroscopy (FCS),^5^, ^6^, ^7^, ^8^, ^9^ including its dual‐color 2‐channel variant fluorescence cross‐correlation spectroscopy (FCCS),^10^, ^11^ and photoactivated localization microscopy (PALM).^12^, ^13^, ^14^, ^15^ Through these approaches, we characterized the dynamics and lateral organization of 3 opioid receptors: the kappa opioid receptor (KOP), the wild type mu opioid receptor (MOP_wt_) and the naturally occurring MOP isoform (MOP_N40D_) where asparagine at position 40 in the N‐terminal domain is substituted by aspartic acid as a result of an A118G single nucleotide polymorphism (SNP) in the human OPRM1 gene.^16^ These G protein‐coupled receptor (GPCR) family members modulate a number of vital physiological processes and play an important role in respiratory, immune and neuroendocrine system function.^17^ In particular, the MOP and KOP receptors are important in both pain and reward processing, and they are major pharmacological targets for the management of chronic pain.^18^, ^19^, ^20^, ^21^ MOP_N40D_ is also clinically relevant in pain. Compared to MOP_wt_, this variant increased pain sensitivity and decreased the pain‐soothing effects of opiates.^22^

Opioid receptor signaling cascades have been well characterized in terms of interaction partners and key signaling events.^23^, ^24^, ^25^, ^26^ What remains largely unknown is the nanoscopic organization of opioid receptors into signaling domains. Different studies show that opioid receptors can form monomers, dimers and even higher order oligomers.^27^, ^28^, ^29^, ^30^, ^31^, ^32^, ^33^, ^34^, ^35^, ^36^, ^37^, ^38^, ^39^, ^40^, ^41^, ^42^, ^43^, ^44^, ^45^ Moreover, lateral dynamics of opioid receptors in the plasma membrane is complex,^46^, ^47^ and can be affected by a number of factors including plasma membrane lipid composition,^48^, ^49^, ^50^, ^51^ stimulation with specific ligands^52^ and heterologous activation of other GPCRs.^53^ While details are still debated, these studies suggest that opioid receptors may have intricate spatiotemporal signaling profiles.^36^, ^49^, ^52^, ^54^ However, quantitative characterization of the complex spatiotemporal organization of different opioid receptors in the plasma membrane remains limited.

FCS and PALM are exceptional tools for this task because they are quantitative, noninvasive, highly sensitive, and offer high spatial and temporal resolution.^5^, ^6^, ^7^, ^8^, ^9^, ^12^, ^13^, ^14^, ^15^ Furthermore, the 2 approaches provide complementary readouts. FCS performed on live cells yields information about modes of molecular motion (free Brownian diffusion, hindered diffusion due to obstacles and/or transient trapping, directed motion) and can detect aggregates that are dynamically linked.^5^ At the same time, PALM and other pointillistic super‐resolution microscopy techniques map the distribution of proteins with a spatial resolution of 10 to 25 nm, an order of magnitude below the spatial resolution of FCS.^12^, ^13^, ^14^, ^15^, ^55^, ^56^, ^57^, ^58^, ^59^, ^60^ To characterize opioid receptor distribution and dynamics, we combined FCS and PALM. Measurements were performed in 2 cell lines genetically modified to express different opioid receptors fused with fluorescent proteins (functionality of receptors was confirmed by agonist treatment, Figures S1‐S3). By integrating results obtained by FCS and PALM we were able to cross‐validate our analysis and interpretation of the data, thus adding significant value to the overall conclusions. Importantly, combination of FCS and PALM can be extended to other GPCRs and used to investigate the effects of pharmacological interventions on receptor partitioning and subsequent sorting into different plasma membrane domains. As such, the approach may ultimately aid in drug discovery.

## 2 BRIEF METHODOLOGICAL BACKGROUND ON FCS/FCCS AND PALM/PC‐PALM

### 2.1 Fluorescence correlation spectroscopy

**Figure 1:**
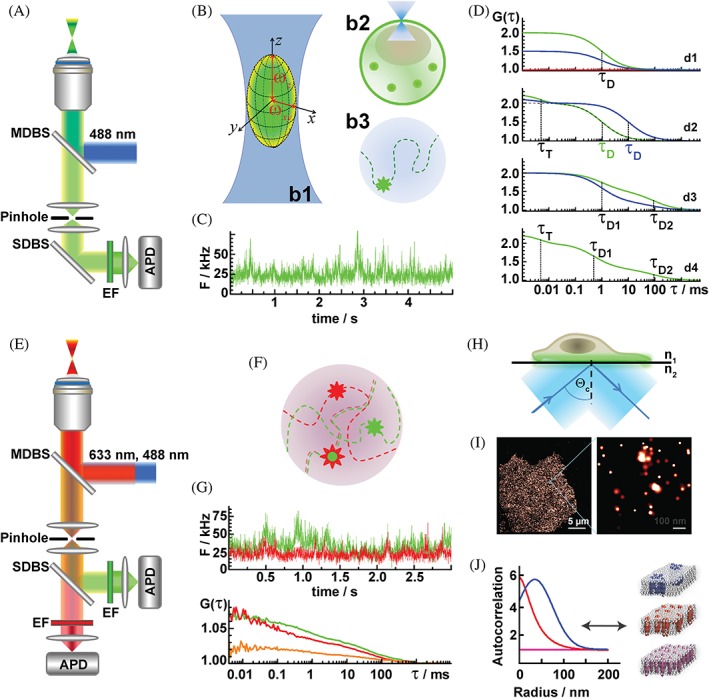
Schematic presentation of the instrumental setup and experimental design for FCS/FCCS and PC‐PALM studies. (A) Schematic drawing of the optical arrangement in an inverted epifluorescence confocal microscope for single‐color FCS measurements. Incident laser light (blue) is reflected by the main dichroic beam splitter (MDBS) and sharply focused by the objective into the sample, generating a double‐conus‐like illumination volume. The elastically scattered incident light (blue) and the spectrally distinct fluorescence (green‐yellow) are first collected by the objective, and then separated by the MDBS that reflects the elastically scattered light and allows the fluorescence light, which is of longer wavelength, to pass through the pinhole and the emission filter (EF) to the APD detector. For single‐color FCS measurements the secondary dichroic beam splitter (SDBS) is not obligatory, since fluorescence can be directly guided to the APD. The depicted setting is used to retain identical optical settings for the green fluorophore in FCS and FCCS (presented below, E). (B) Schematic presentation of the observation volume element (OVE). b1: Magnified image of the 3D double‐cone‐like illumination volume generated in the sample by focusing the incident laser light with a microscope objective (blue) and the idealized OVE in the form of a prolate ellipsoid from which fluorescence is being detected. *ω*_*xy*_ and *ω*_*z*_ are the 1/*e* ^2^ radial and axial radius of the OVE, respectively. For clarity, the incident (blue) and fluorescence (green) light were shown separately, while in reality they overlap (as shown in A). b2: For FCS/FCCS measurements, laser light is focused at the apical plasma membrane of a stably transformed PC12 cell, above the virtually transparent cell nucleus, in order to minimize background contribution from the cytoplasm. b3: For FCS measurements at the plasma membrane, the OVE is a 2D plane, schematically depicted as a circle with a radius *ω*_*xy*_. (C) Photons emitted by fluorescent molecules passing through the OVE are detected by an APD, which responds with an electrical pulse to each detected photon. The number of electrical pulses originating from photons detected during a specific time interval, the so‐called binning time, corresponds to the measured light intensity at a given point of time. An example is shown here of a fluorescence intensity fluctuation time series recorded at the plasma membrane of a PC12 cell stably transformed to express MOP‐eGFP. The electrical signal is transferred to a digital signal correlation unit and the corresponding normalized autocorrelation function *G*(*τ*) is calculated on‐line to yield an experimentally derived temporal autocorrelation curve. (D) Different theoretical model functions for fitting tACCs. d1: Randomly generated fluorescence intensity fluctuations do not give rise to a tACC (red), whereas processes with an underlying time constant yield distinct tACCs (green and blue)—model functions for 2D diffusion of a single fluorescent species: *G*(*τ*) = 1 + (1/*N*)(1/(1 + *τ*/*τ*_D_)), when the average number of molecules in the OVE is *N* = 1 (green) and *N* = 2 (blue), in both cases *τ*_D_ = 1 ms. d2: Model functions for 2D diffusion of a single fluorescent species and triplet formation with a characteristic decay time *τ*_T_ and an average equilibrium fraction of molecules in the triplet state *T*: *G*(*τ*) = 1 + (1/*N*)(1/(1 + *τ*/*τ*_D_))(1 + (*T*/(1 − *T*)) exp(−*τ*/*τ*_T_)), when *N* = 1, *τ*_D_ = 1 ms, *τ*_T_ = 5 μs and *T* = 0.2 (green) and *N* = 1, *τ*_D_ = 10 ms, *τ*_T_ = 5 μs and *T* = 0.1 (blue). The dashed line shows the tACC for 2D diffusion of a single component without triplet formation when *N* = 1 and *τ*_D_ = 1 ms. d3: Model functions for 2D diffusion of 2 fluorescent species with different diffusion times and different relative contribution of the slow component (*y*): *G*(*τ*) = 1 + (1/*N*)(1 − *y*)/(1/(1 + *τ*/*τ*_D1_) + *y*/(1 + *τ*/*τ*_D2_)), when *N* = 1, *τ*_D1_ = 1 ms, *τ*_D2_ = 90 ms, *y* = 0.2 (blue) and *N* = 1, *τ*_D1_ = 1 ms, *τ*_D2_ = 90 ms, *y* = 0.5 (green). d4: Model function for 2D diffusion of 2 fluorescent species with different diffusion times (*τ*_D1_ = 500 μs and *τ*_D2_ = 90 ms), relative contribution of the slow component (*y* = 0.3) and of the triplet (*τ*_T_ = 5 μs and *T* = 0.2): *G*(*τ*) = 1 + (1/*N*)((1 − *y*)/(1/(1 + *τ*/*τ*_D1_) + *y*/(1 + *τ*/*τ*_D2_))(1 + (*T*/(1 − *T*)) exp(−*τ*/*τ*_T_)). (E) Schematic drawing of the optical arrangement in an inverted epifluorescence confocal microscope for dual‐color FCCS measurements. Incident light from 2 lasers, 488 nm (blue) and 633 nm (red), are combined and reflected by the MDBS and sharply focused by the objective into the sample. The elastically scattered incident light from both lasers (blue and red) and the spectrally distinct fluorescence (green‐yellow and far red) are first collected by the objective, and then separated by the MDBS. The SDBS separates the emission of the longer wavelength (far red) fluorophore from the emission of the shorter wavelength (green). The emitted light is further spectrally narrowed by passing through matching EFs and detected by APD detectors. (F) As fluorescent molecules pass through the detection volume, the dually labeled molecules give rise to fluctuations in fluorescence intensity in both channels simultaneously (green and red dashed line), while this is not the case for the singly labeled molecules. (G) Top**:** Fluorescence intensity fluctuations recorded at the plasma membrane of a PC12 cell stably transformed to express 2 GPCR representatives genetically fused with spectrally distinct fluorescent proteins (MOP_wt_‐eGFP and serotonin 5‐HT1_A_‐Tomato). Bottom***:*** Corresponding tACCs (green and red) and tCCC (orange). (H) Schematic drawing of sample illumination in a TIRF microscope. The incident laser light, which enters at the critical angle (*θ*_c_), is reflected at the interface between the sample and the coverslip (*n*_1_ < *n*_2_) and an evanescent field is generated that penetrates about 100 to 200 nm into the sample. Only fluorophores in the evanescent field are excited, as indicated by the green color. (I) TIRF image of MOP_wt_‐paGFP in a COS‐7 cell (left) with zoom‐in (right). (J) sACC (left) for different lateral organizations. Random monomers show no spatial correlation (magenta); random oligomers are characterized by short‐length correlations that follows an exponential decay (red); and complex organizations is characterized by short‐ and long‐length correlations, yielding sACCs that follow a two‐function decay (blue)

FCS is a quantitative analytical method with single‐molecule sensitivity designed for detection of bright fluorescent molecules in dilute solutions. Originally developed for applications in physical chemistry to measure the kinetics of chemical reactions in systems at equilibrium,^9^, ^61^, ^62^ FCS is becoming widely used in cell biology as it enables quantitative biochemical measurements in live cells. In particular, FCS can be used to nondestructively measure molecular concentration in different cellular compartments, characterize their local transporting properties (diffusion and trafficking), and the kinetics of their interactions.^5^, ^10^, ^63^, ^64^, ^65^ To this aim, spontaneous fluorescence intensity fluctuations around a steady state are monitored with high temporal resolution in a very small volume. To generate this tiny volume, the conventional instrumentation for FCS takes advantage of the specific arrangement of optical elements in an inverted epifluorescence confocal microscope. In such a microscope, the incident laser light is sharply focused into the sample through a high numerical aperture (NA) objective and fluorescence is collected by the same objective. The volume from which fluorescence is detected is further reduced by placing a pinhole in the optically conjugate plane in front of the detector to eliminate out‐of‐focus light (Figure 1A). In this way, a miniature observation volume element (OVE) is generated in the sample (the yellow‐green prolate ellipsoid in Figure 1B, b1). For confocal laser scanning microscopy (CLSM) imaging, the OVE is scanned over the sample and fluorescence intensity at a specific location is recorded to map the spatial distribution, that is, to generate an image. For FCS measurements, the OVE is positioned in a specific location (Figure 1B, b2) and fluorescence intensity fluctuations that arise due to spontaneous, thermally driven microscopic changes in the positions of molecules through the OVE (schematically depicted in Figure 1B, b3) are recorded with sub‐microsecond temporal resolution (Figure 1C). The small size of the OVE is crucial to enable observation of tiny fluctuations in fluorescence intensity. In conventional systems, the OVE size is limited by the diffraction of light, and its volume is typically several tenths of a femtoliter. In such a tiny volume, the number of fluorescent molecules is small (for a 10‐nM solution and OVE of 0.17 fL, the average number of molecules in the OVE is 1). By looking at a small number of molecules at a time, the background noise originating from molecules present in a large excess, such as solvent molecules, is significantly reduced. Hence, passage of a bright fluorescent molecule through the small OVE gives rise to a prominent change in fluorescence intensity that can be readily detected (Figure 1C). Fluctuations in fluorescence intensity are then analyzed to extract information about: (1) the average number of molecules in the OVE (*N*), which depends on the concentration and (2) the average transition time, that is, the time needed for a molecule to pass through the OVE by translational diffusion, the so‐called translation diffusion time (*τ*_D_). *τ*_D_ is defined by the diffusion coefficient (*D*) and the size of the OVE: *τ*_D_ = *ω* ^2^_*xy*_/4*D* , where *ω*_*xy*_ is the axial radius of the OVE (Figure 1B, b1), that is, *ω* ^2^ *xy* is the waist area of the OVE.

While different signal processing approaches can be used to analyze the fluorescence intensity fluctuations and extract quantitative information about processes that give rise to them,^5^, ^66^ the originally proposed and most often used temporal autocorrelation analysis is applied in this study. The first step in temporal autocorrelation analysis is to determine whether the experimentally recorded fluorescence intensity fluctuations are generated by a random process, such as noise, or by processes that appear with a certain characteristic time, such as molecular diffusion or fluorescence twinkling due to photophysical or chemical processes. To establish this, the signal time series is subjected to self‐similarity analysis, that is, the signal is compared to a copy of itself delayed for a certain lag time (*τ*) and the so‐called normalized autocorrelation function *G*(*τ*) is calculated to establish whether the fluorescence intensity observed at one point in time (*F*(*t*)) in the analyzed time series is correlated with the value at (*F*(*t* + *τ*)):

(1a) $$G\left(\tau \right)=\frac{〈F\left(t\right)\cdot F\left(t+\tau \right)〉}{{〈F\left(t\right)〉}^{2}}.$$

Here, chevron brackets denote average values of the analyzed variables over time. Since fluorescence intensity can be represented as fluorescence intensity fluctuation over the mean fluorescence intensity 〈*F*(*t*)〉, it is also possible to express the normalized autocorrelation function using the deviation of fluorescence intensities from its mean value, *δF*(*t*) = *F*(*t*) − 〈*F*(*t*)〉 and *δF*(*t* + *τ*) = *F*(*t* + *τ*) − 〈*F*(*t*)〉:

(1b) $$G\left(\tau \right)=1+\frac{〈\mathrm{\delta F}\left(t\right)\cdot \mathrm{\delta F}\left(t+\tau \right)〉}{{〈F\left(t\right)〉}^{2}}.$$

We then examine whether *G*(*τ*) is dependent on *τ* by plotting *G*(*τ*) = *f*(*τ*). This graph is known as the temporal autocorrelation curve (tACC; Figure 1D, d1‐d4). When the fluorescence intensity observed at one point in time (*F*(*t*)) is not correlated with its value at any other point in time (*F*(*t* + *τ*)), random variations of *G*(*τ*) around the value *G*(*τ*) = 1 are observed (Figure 1D, d1, red). When the fluctuations are not random, a tACC is obtained that is characterized by a maximal limiting value of *G*(*τ*) as *τ* → 0, which decreases to the value of *G*(*τ*) = 1 at long lag times, indicating that correlation between the fluorescence intensities is lost (Figure 1D, d1 green and blue). If there is only one process that gives rise to fluorescence intensity fluctuations, the tACC shows only one inflection point, that is, one characteristic decay time (Figure 1D, d1 green and blue). If there are more processes giving rise to fluorescence intensity fluctuations that occur at different time scales, the tACC assumes a more complex shape with more than one characteristic decay time (Figure 1D, d3 and d4).

The zero‐lag amplitude of the tACC (*G*_0_ = *G*(0) − 1) and the characteristic decay time of the tACC yield valuable quantitative information about the investigated system. When fluorescence intensity fluctuations arise due to molecular diffusion, the zero‐lag amplitude of the tACC provides information about the concentration of fluorescent molecules as it equals the inverse average number of molecules in the OVE (*N*). In Figure 1D: d1, the green tACC corresponds to the case when *N* = 1, and the blue tACC represents the case when *N* = 2. Thus, the amplitude of the tACC decreases as the number of molecules in the OVE increases. The characteristic decay time of the tACC gives information about the rates at which processes that give rise to the fluorescence intensity fluctuations occur. When fluorescence intensity fluctuations are generated by molecular diffusion, the characteristic decay time of the tACC reflects the average time it takes for a molecule to cross through the OVE by translational diffusion. In Figure 1D: d2, *τ*_D_ = 1 ms for the green tACC and *τ*_D_ = 10 ms for the blue tACC with a clearly longer decay time. For an unabridged derivation of the underlying relationships, see References 5 and 67 and http://www.fcsxpert.com/classroom/theory/.

In order to read out *N* and *τ*_D_ from the experimentally derived tACCs, fitting with theoretical model functions is performed. Several model functions relevant for this study are represented in Figure 1D, d1‐d4 and described in detail in the figure legend. Whenever possible, selection of an appropriate analytical model needs to be based on prior knowledge of the composition of the system. If this prior knowledge does not exist, a model with the smallest number of parameters should be used that is sufficient to account for the experimental measurements.

### 2.2 Fluorescence cross‐correlation spectroscopy

To quantitatively characterize molecular interactions in live cells by FCCS, spectrally distinct fluorophores (emitting, e.g., in the green and the red region of the visible spectrum) are used to specifically label the molecules of interest. Fluorescence intensity fluctuations are simultaneously recorded with high temporal resolution using overlapping excitation pathways and separate detector pathways (Figure 1E). The experimentally recorded fluorescence intensity fluctuations are then processed using temporal auto‐ and cross‐correlation analysis. In that manner, we distinguish the unbound, independently diffusing singly labeled molecules from the co‐diffusing dually labeled bound molecules that give rise to fluorescence intensity fluctuations in both detectors simultaneously (Figure 1F). This analysis yields 2 individual, yet simultaneously recorded fluorescence intensity time series (Figure 1G, top) from which 2 tACCs for the unbound molecules (Figure 1G, green and red) and a temporal cross‐correlation curve (tCCC) for the dually labeled bound molecules (Figure 1G, orange) are derived by temporal auto‐ and cross‐correlation analysis, respectively. As in FCS, the zero‐lag amplitudes of the tACCs reflect the average number of molecules in the OVE, with the distinction that this value now is a sum of the number of unbound, singly labeled, and bound, dually labeled molecules. Thus, the total number of green‐labeled molecules is *N*_g,t_ = *N*_g_ + *N*_gr_, and the total number of red‐labeled molecules is *N*_r,t_ = *N*_r_ + *N*_gr_. Correlation of fluorescence intensity fluctuations between the channels, that is, cross‐correlation, identifies the dually labeled molecules only, since their passage through the OVE gives rise to fluorescence intensity fluctuation in both detectors simultaneously. Thus, cross‐correlation examines whether fluorescence intensity observed in one detector at one point in time, for example, *F*_green_(*t*) is correlated with the fluorescence signal in the other detector at *F*_red_(*t* + *τ*). The cross‐correlation function is:

(2) $$G_{\mathrm{CC}}\left(\tau \right)=1+\frac{〈\delta F_{\text{green}}\left(t\right)\cdot \delta F_{\mathrm{red}}\left(t+\tau \right)〉}{〈F_{\text{green}}\left(t\right)〉〈F_{\mathrm{red}}\left(t\right)〉},$$

plotted for different lag times yields the tCCC (Figure 1G, orange). In the absence of cross‐talk, the zero‐lag amplitude of the tCCC, *G*_CC,0_ = *G*_CC_(0) − 1, is directly proportional to the number of dually labeled molecules, *N*_gr_:

(3) $$G_{\mathrm{CC}}\left(0\right)-1\propto \frac{N_{\mathrm{gr}}}{\left(N_{g}+N_{\mathrm{gr}}\right)\cdot \left(N_{r}+N_{\mathrm{gr}}\right)}$$

Thus, for a constant total number of green‐ and red‐labeled molecules (*N*_g,t_ = *N*_g_ + *N*_rg_ and *N*_r,t_ = *N*_r_ + *N*_rg_), the amplitude of the tCCC increases for increasing number of dually labeled molecules (*N*_gr_).

### 2.3 Photoactivated localization microscopy

Pointilistic super‐resolution fluorescence microscopy imaging technique PALM^12^ relies on the use of photoinducible fluorescence reporters, such as the photoactivatable green fluorescent protein (paGFP)^68^ that can cycle to/from a metastable dark state when stimulated by light of a specific wavelength. Only a fraction of molecules are imaged at any time point and individual fluorophores can be resolved with a localization precision below the conventional diffraction limit (<200 nm). The precision by which a single fluorescent molecule can be localized by PALM and related techniques, depends on the instrumental setup, reflected by the width (*s*_*i*_) of the point spread function (PSF) and the collected number of photons (*n*)—the smaller *s*_*i*_ and the larger *n*, the better the localization precision (*σ*).^12^, ^13^, ^69^ It is therefore important to detect as many photons as possible from each molecule.

In order to optimally detect the relatively faint emission from single‐molecules localized in the plasma membrane, total internal reflection fluorescence (TIRF) is used. TIRF illumination generates an evanescent excitation wave that penetrates 100 to 200 nm into the specimen, leading to significantly reduced background fluorescence (Figure 1H). The sequence of events for determining the precise location of paGFP is as follows. Initially, all molecules in the specimen are inactive (native nonemissive state). A 488 nm laser is used to simultaneously photoactivate and excite a subset of molecules in the specimen that are positioned at distances >200 nm. Photoactivation occurs stochastically, and the number of activated paGFP molecules is maintained low by ensuring that the laser intensity is sufficiently weak at the focal plane. The 488 nm laser is also used to excite the activated molecules triggering fluorescence, while an electron multiplying charge‐coupled device (EMCCD) camera records emitted photons. During acquisition, the photoactivated molecules are spontaneously and irreversibly photobleached. A new subset of molecules is then photoactivated, recorded and photobleached. This sequence is repeated until all molecules in the specimen have been localized and exhausted. Typically, 10,000 to 30,000 diffraction‐limited images are acquired to yield 1 super‐resolution image. Data analysis is performed thereafter to identify single molecules above background noise, calculate the PSFs for the molecules and determine their centers with a specific localization precision. paGFP molecules are typically localized with a precision of 10 to 25 nm in a super‐resolution PALM image (Figure 1I).

### 2.4 Pair‐correlation photoactivated localization microscopy

Pair‐correlation photoactivated localization microscopy (PC‐PALM)^14^, ^15^ utilizes the radial distribution function, *g*(*r*), also called the pair‐correlation function, to determine the probability of finding the center of a molecule at a given distance from the center of a reference molecule in a PALM image. By plotting the amplitude of *g*(*r*) as a function of radial distance (*r*), a spatial autocorrelation curve (sACC) is derived (Figure 1J, left). From sACCs, information about spatial organization of molecules in the plasma membrane can be extracted (Figure 1J, right). For example, in the case of randomly distributed monomers, spatial correlation is not observed and the sACC yields values around 1 for all radial distances (Figure 1J, magenta). In the case of randomly distributed oligomers, strong correlation is observed at short lengths, which exponentially decays with distance (Figure 1J, red). In the case of more complex lateral organization, such as clustered dimers, complex sACCs with short‐ and long‐ correlation lengths can be expected (Figure 1J, blue). From these sACCs important organization parameters can be extracted (eg, nanodomain size and protein domain occupancy).

## 3 RESULTS

### 3.1 Opioid receptors differ in nanoscale lateral dynamics and spatial organization in the plasma membrane

**Figure 2:**
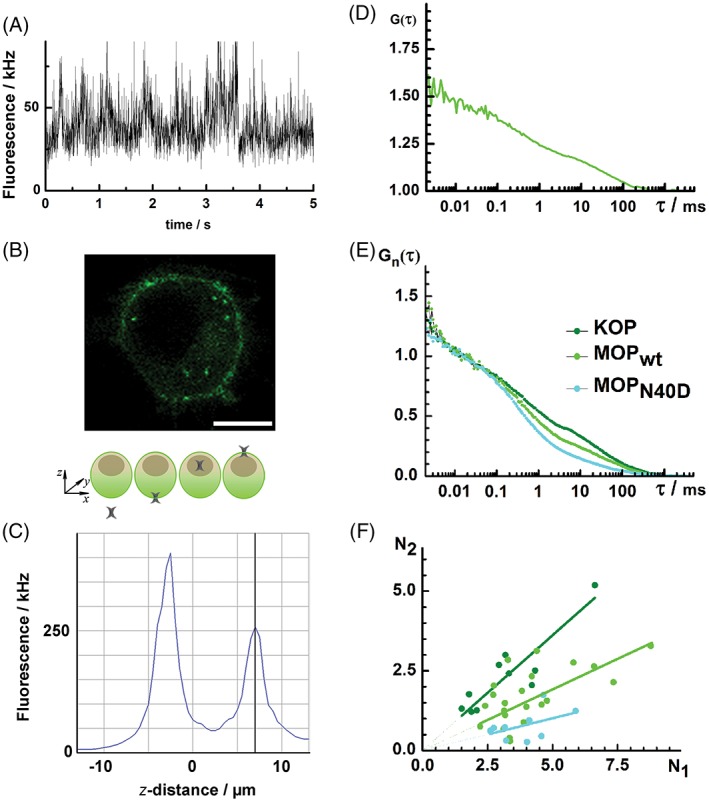
Opioid receptor lateral dynamics in the plasma membrane characterized by FCS in live PC12 cells. (A) Fluorescence intensity fluctuation time series recorded at the apical plasma membrane of live PC12 cells stably transformed to express the wild type mu‐opioid receptor fluorescently tagged with the enhanced green fluorescent protein (MOP_wt_‐eGFP). For clarity, first 5 seconds out of a 10 seconds measurement are shown. (B) CLSM image of a live PC12 cell stably transformed to express MOP_wt_‐eGFP. Scale bar = 5 μm. (C) Fluorescence intensity profile across a single PC12 cell expressing MOP_wt_‐eGFP determined by a linear scan in the axial direction (z‐scan; step size, 0.5 μm), as schematically depicted in the panel above. The first and the second fluorescence intensity maximums indicate the position of the basal and apical plasma membrane, respectively. (D) Representative temporal autocorrelation curve (tACC) for MOP_wt_‐eGFP recorded at the apical plasma membrane of 1 cell (average of 10 consecutive measurements, each measurement lasting for 10 seconds). (E) Average tACC normalized to the same amplitude, *G*_n_(*τ*) = 1 at *τ* = 10 μs, derived by averaging normalized tACC recorded at the apical plasma membrane of 8 to 10 cells for each opioid receptor. Each individual average tACC is an average of 10 tACC obtained in 10 consecutive 10 seconds measurements. The complex shape of the tACC indicates that processes with different characteristic times contribute to the fluorescence intensity fluctuations: *τ* < 200 μs, 200 μs < *τ*_D1_ < 1 ms, 10 ms < *τ*_D2_ < 250 ms. (F) Number of molecules (*N*_2_) characterized by the long diffusion time (*τ*_D2_) as a function of the number of molecules (*N*_1_) characterized by the short diffusion time (*τ*_D1_). Points indicate measurements on individual cells. The total number of molecules in the observation volume element is the sum, *N* = *N*_1_ + *N*_2_. Slopes of the fitted lines show the fraction of molecules characterized by slow diffusion over the fraction of molecules characterized by fast diffusion, which is (0.42 ± 0.06) for KOP‐eGFP, (0.30 ± 0.15) for MOP_wt_‐eGFP and (0.16 ± 0.07) for MOP_N40D_‐eGFP. Additional statistical considerations for panels D and E are provided in Table S1

Fluorescence intensity fluctuations (Figure 2A) were recorded at the apical plasma membrane of live PC12 cells stably transformed to express opioid receptors tagged with the enhanced green fluorescent protein (eGFP) at the C‐terminal end (Figure 2B). Measurements were performed above the nearly transparent cell nucleus, where the contribution of fluorescence from the cytoplasmic fraction is minimal (Figure 2C). Results were analyzed by temporal autocorrelation to obtain tACCS (Figure 2D).

Temporal autocorrelation analysis for all investigated opioid receptors yielded complex tACCs with more than 1 characteristic decay time (Figure 2E). Control experiments and fitting analysis of tACCs are explained in detail in Appendix S1, Supporting Information: FCS and FCCS, and in Figures S4‐S7. We identified (1) a short decay time, *τ* < 200 μs, that is not related to molecular diffusion but rather to the kinetics of complex photophysical processes and conformational fluctuations of eGFP^70^ and (2) 2 well separated diffusion‐related decay times, 200 μs < *τ*_D1_ < 1 ms and 10 ms < *τ*_D2_ < 250 ms. Of note, the indicated intervals reflect differences between lateral diffusion times measured in different cells, expressing different opioid receptors (KOP, MOP_wt_ or MOP_N40D_), and not differences between consecutive FCS measurements on the same cell.

For all analyzed cells, the investigated opioid receptors displayed a similar short decay time (*τ* < 200 μs), a result made clear after normalizing average tACCs to the same amplitude (Figure 2E). In contrast, the amplitude of the second tACC component, (1 − *y*), which revealed the relative fraction of receptors with the longer diffusion time (*τ*_D2_), was different for different opioid receptors. This fraction was largest for KOP (0.40 ± 0.04), smaller for MOP_wt_ (0.27 ± 0.04), and smallest for MOP_N40D_ (0.19 ± 0.04). Furthermore, the number of opioid receptors (*N*_2_) characterized by *τ*_D2_ was not independent, but rather scaled with the total number of receptors, giving rise to a linear dependence between the number of opioid receptors (*N*_2_) characterized by the longer diffusion time (*τ*_D2_) and the number of opioid receptors (*N*_1_) characterized by the shorter diffusion time (*τ*_D1_) (Figure 2F). Slopes of the fitted lines, showing the fraction of molecules characterized by long diffusion time over the fraction of molecules characterized by the short diffusion time, (1 − *y*)/*y*, were different for different opioid receptors: (0.42 ± 0.06) for KOP‐eGFP, (0.30 ± 0.15) for MOP_wt_‐eGFP and (0.16 ± 0.07) for MOP_N40D_‐eGFP. Differences were statistically significant in all cases (*P* <.05; Table S1). This suggests that the 2 opioid receptor fractions are not independent, but rather dynamically interrelated. This interrelation, which is reflected by the slope of the linear regression in Figure 2F, is different for different opioid receptors.

Taken together, FCS analysis suggests that all investigated opioid receptors partition in the plasma membrane, yielding 2 principal fractions that are dynamically linked and identifiable by differences in lateral diffusion times. In addition, FCS showed that opioid receptors differentially organize: KOP had the largest and MOP_N40D_ the smallest fraction of receptors with a long diffusion time, as evident from the relative amplitudes in Figure 2E and the slopes in Figure 2F.

In order to validate the interpretation of FCS data, PC‐PALM was used to investigate the spatial distribution of KOP, MOP_wt_ and MOP_N40D_ tagged with the paGFP at the C‐terminal. The individual constructs were transiently expressed in COS‐7 cells. According to western blot analyses, expression levels of MOP‐paGFP and KOP‐paGFP were comparable to expression levels of endogenous MOP and KOP found in several other cell lines. As expected, MOP_N40D_‐paGFP was expressed at lower levels compared to MOP_wt_‐paGFP (Figure S3A). No endogenous MOP or KOP was detected in COS‐7 cells. Next, opioid receptor functionality in COS‐7 cells was confirmed by showing that agonists activated downstream effectors: protein kinase B (AKT) and extracellular signal‐regulated kinase (ERK) (Figure S3B).

**Figure 3:**
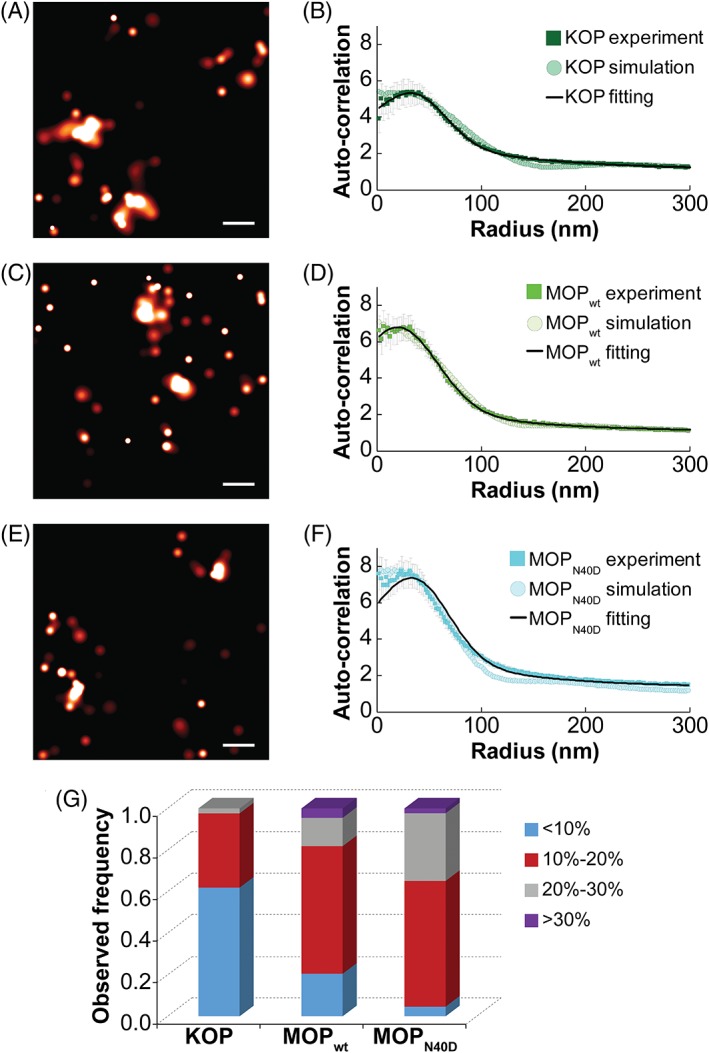
Opioid receptors differ in their spatial organization at the nanoscale level. (A) PALM image of KOP‐paGFP from a region on a single COS‐7 cell (scale bar, 100 nm). (B) The average KOP‐paGFP auto‐correlation function obtained from experimental data (dark green squares; 14 cells, *N* = 42) and simulation (dark green circles); the fitting curve is also shown (black line). (C) PALM image of MOP_wt_‐paGFP from a region on a single COS‐7 cell (scale bar = 100 nm). (D) The average MOP_wt_‐paGFP auto‐correlation function obtained from experimental data (green squares; 14 cells, *N* = 44) and simulation (green circles). The fitting curve is also shown (black line). (E) PALM image of MOP_N40D_‐paGFP from a region on a single COS‐7 cell (scale bar = 100 nm). (F) The average MOP_N40D_‐paGFP auto‐correlation function obtained from experimental data (cyan squares; 15 cells, *N* = 43) and simulation (cyan circles). The fitting curve is also shown (black line). Fitting and simulation results are in agreement for all 3 constructs; the results are summarized in Tables 1 and S2. PALM images were generated by analyzing datasets in PeakSelector^12^ and grouping peaks within a group radius of 3*σ*_MAX_ with maximum dark time of 5 seconds. SE bars are shown. (G) For each ROI, we calculated the fraction of receptors that reside outside of nanodomains. We next provided normalized histogram for such molecules: Fraction of ROIs with less than 10% of receptors residing outside nanodomains is in blue; with 10% to 20% in red; with 20% to 30% in gray and with more than 30% in purple. The average values with SEs were 10.0 ± 0.7% (KOP‐paGFP); 15 ± 1% (MOP_wt_‐paGFP) and 18.1 ± 0.9% (MOP_N40D_‐paGFP). *P*‐value between KOP‐paGFP and MOP_wt_‐paGFP is 4 × 10^−5^; *P*‐value between MOP_wt_‐paGFP and MOP_N40D_‐paGFP is .03. Thus, KOP‐paGFP has the smallest and MOP_N40D_‐paGFP has the largest fraction of receptors that reside outside of nanodomains

We next acquired PALM images of opioid receptors. As described in the methodological background and similarly to previous work,^14^, ^15^, ^71^ the 488 nm laser light was used to activate and detect paGFP. Using our PALM image localization protocol (based on Peak Selector software,^12^ and described in detail in Appendix S1), we obtained average localization precisions with SDs of (15.8 ± 1.5), (16.5 ± 1.0) and (16.2 ± 1.5) nm for MOP_wt_, KOP and MOP_N40D_, respectively (Figure 3A,C,E). These low SDs highlight strong reproducibility in localization precision values across different cells. From localized peak datasets, we also calculated molecular densities similarly as described before.^14^, ^15^ MOP_wt_ and KOP surface densities were comparable, with (52 ± 4) and (51 ± 4) detected molecules/μm^2^ on average, respectively. In contrast, MOP_N40D_ density was lower, with (43 ± 3) detected molecules/μm^2^ (*P* < .03). These results are consistent with the western blot shown in Figure S3A. Localized peak datasets were then used to calculate spatial correlation curves (sACCs) using PC analysis.^14^, ^15^

Interestingly, our data suggest that opioid receptors are not randomly distributed. Rather, they exhibit distinct lateral organization, which is evident from the complex sACCs with 2 characteristic correlation lengths (Figure 3B,D,F). In evaluating the sACC by fitting, classical approaches using a single exponential decay function failed to properly represent the experimental data. Instead, a Gaussian function was used to fit the sACC at short distances and an exponential function was used for longer distances.^72^ Based on this analysis, we identified that opioid receptors are organized into nanodomains (indicated by long‐distance correlations) and their organization within these nanodomains is not random (indicated by short‐distance correlations). Results from fitting analysis, summarized in Table 1, suggest that domain radius and occupancy increase in the following order: MOP_N40D_, MOP_wt_ and KOP. At the same time, MOP_N40D_ had the highest and KOP had the lowest increased local density in domains (increased local density in domains is defined as domain density compared to the average cell density).

**Table 1:** Lateral organization of KOP, MOP_wt_ and MOP_N40D_ at the nanoscale level obtained by fitting of sACC with PC‐PALM and by ensemble‐averaged MC simulations

	KOP‐paGFP		MOP_wt_‐paGFP		MOP_N40D_‐paGFP	
	Fitting	Simulation	Fitting	Simulation	Fitting	Simulation
Detected proteins per domain	9.4	9‐10	8.1	8‐9	7.9	7‐8
Domain radius (nm)	105	101	92	86	82	79
Increased local density in domains	5.3	5.2‐5.8^a^	5.8	6.4‐7.2^a^	8.8	7.8‐8.9^a^

a Calculated values.

Interpretation of PC‐PALM data by fitting analysis was first validated using model proteins vesicular stomatitis viral glycoprotein (VSVG) and glycosyl‐phosphatidylinositol‐anchored protein (GPI), Figure S8. Parameters obtained from sACC curves agree well with previously published data: we detected largely trimers for VSVG and small clusters with few random monomeric regions for GPI.^14^, ^71^, ^73^, ^74^, ^75^ Interpretations were further validated by ensemble‐averaged Monte Carlo (MC) simulations.^76^ To this end, a model convolved with realistic approximations to the instrument response (photon count distributions, PSFs, average localization precisions, average number of appearances and density of appearances) was used to derive images of opioid receptor domains by simulation (details are given in Appendix S1: MC simulations). From these images, we generated position estimates, calculated correlation functions (using the same software and algorithms used to analyze experimental data) and compared them with experimental results. Simulation and fitting results summarized in Table 1 show excellent agreement. Statistical considerations are provided in Table S2. For all opioid receptors studied, we show that the variances of 2 populations (experimental and best‐fit simulated curves) are equal within 5%. Additionally, we used simulations to explore alternative organization features. We show that the experimental data do not follow a single exponential function, nor do they fit simple distributions of molecules (Figure S9A‐C). Such distributions include 1 to 4 proteins in tight random domains (ie, oligomers) and proteins arbitrarily organized in domains with the size and occupancy predicted from experiments (Table 1). We also show alternative complex distributions that did not produce a good fit, such as the combination of smaller and larger domains (Figure S9D); and small changes in domain occupancy/radius from best‐fit reported in Table 1 (Figure S9E). Statistical considerations shown in Table S2 imply that changes in domain occupancy for 1 protein, or changes in domain radius for ~10 nm result in significant variations in sACCs that do not agree well with experimental curves. Seeding for the model was robust; obtained distributions appear to be qualitatively the same irrespective of which initial seed value was used for the simulations (Figure S10).

To account for opioid receptors that reside outside nanodomains, we extended the PC‐PALM methodology and calculated the fraction of these receptors. We used a clustering algorithm (described in detail in Section 6) that incorporated the domain size of opioid receptors obtained from fitting analysis. As shown in Figure 3G, KOP has the smallest fraction and MOP_N40D_ has the largest fraction of molecules residing outside the nanodomains, a result consistent with our FCS data (Figure 2E,F).

Thus, PC‐PALM, complemented by MC simulations, confirms that opioid receptors display complex lateral organization—they organize into nanodomains and their organization within these nanodomains is not random. The domain radius and occupancy increased, while the increased local density in domains decreased in the following order: MOP_N40D_, MOP_wt_ and KOP. Finally, the fraction of opioid receptors that reside outside nanodomains is the smallest for KOP and the largest for MOP_N40D_.

### 3.2 Opioid receptors partially associate with cholesterol‐enriched domains

**Figure 4:**
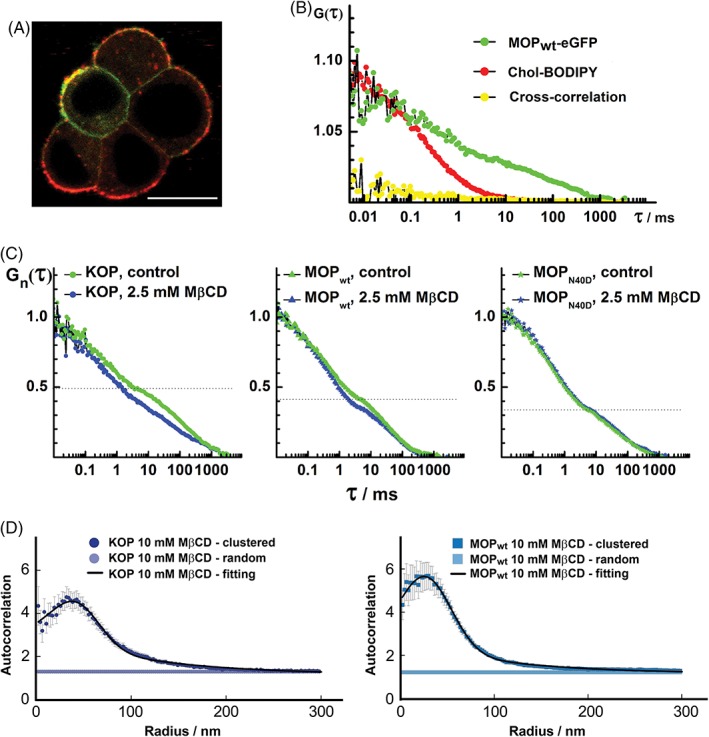
Opioid receptors partially associate with cholesterol‐enriched domains. (A) CLSM image of live stably transformed PC12 cells showing partial colocalization (yellow) between MOP_wt_‐eGFP (green) and cholesteryl‐BODIPY (red). Scale bar = 10 μm. (B) FCCS shows that in the region where colocalization between MOP_wt_‐eGFP (green) and cholesteryl‐BODIPY (red) is observed, cross‐correlation is not observed (yellow/black). (C) FCS shows that cholesterol depletion by 2.5 mM MβCD somewhat alters KOP and MOP_wt_ but not MOP_N40D_ dynamics in the plasma membrane, as evident from the differences in normalized tACC recorded before (green) and after treatment with MβCD (blue). The tACC shown are average curves normalized to the same amplitude, *G*_n_(*τ*) = 1 at *τ* = 10 μs, obtained for each opioid receptor subtype from measurements on 10 cells (ie, from 10 × 10 individual tACC). Additional statistical considerations for panels B and C are provided in Table S1. (D) Auto‐correlation curves from PC‐PALM analysis show that cholesterol depletion leads to random spatial organization of opioid receptors in a number of investigated regions and decreases the number of opioid receptors in domains in nonrandom regions when compared to steady state. The average KOP‐paGFP auto‐correlation function obtained from experimental data for nonrandom (dark blue circles; *N* = 18) and random organization (blue circles; *N* = 24, 57% of ROIs); the fitting curve is shown in black (107 nm domain radius, 6.5 detected proteins per domain and 4.4 increased local density in domains). In total, 19 cells were imaged; 6 cells had ROIs with both nonrandom and random organizations. The average MOP_wt_‐paGFP auto‐correlation function obtained from experimental data for nonrandom (blue squares) and random organization (light blue squares; *N* = 15, 33% of ROIs); the fitting curve is shown in black (117 nm domain radius, 5.2 detected proteins per domain and 3.0 increased local density in domains). In total, 13 cells were imaged; 7 cells had ROIs with both nonrandom and random organizations. Additional statistical considerations for panel D are provided in Table S2

The fluorescently labeled cholesterol analog Cholesteryl 4,4‐Difluoro‐5‐(4‐Methoxyphenyl)‐4‐Bora‐3a,4a‐Diaza‐s‐Indacene‐3‐Undecanoate (cholesteryl‐BODIPY) and methyl‐β‐cyclodextrin (MβCD) were used to probe opioid receptor association with cholesterol‐enriched domains (Figure 4). CLSM imaging revealed a nonhomogeneous distribution of cholesteryl‐BODIPY across the plasma membrane in live PC12 cells expressing MOP_wt_ (Figure 4A). Images show: (1) short (~500 nm) interspaced regions where cholesterol‐ (red) and opioid receptor‐ (green) enriched domains interchange; (2) macroscopic areas where one or the other fluorescence signal prevails over long distances (several μm) and (3) regions where cholesterol and opioid receptor colocalize in domains that are smaller than the size of the confocal volume (<200 nm, yellow).

In areas where colocalization was observed, interactions between opioid receptors and cholesteryl‐BODIPY were examined by FCCS (Figure 4B). FCCS showed that the lateral mobility of cholesteryl‐BODIPY is higher than that of opioid receptors, as evident from the significantly shorter tACC decay time for cholesteryl‐BODIPY lateral diffusion (Figure 4B, red), compared to the tACC decay time for MOP_wt_ lateral diffusion (Figure 4B, green). Moreover, cross‐correlation between the cholesteryl‐BODIPY and the MOP_wt_‐eGFP signal was not observed (Figure 4B, yellow), suggesting that cholesteryl‐BODIPY does not bind to eGFP‐tagged opioid receptors in the regions where colocalization was observed by CLSM imaging.

Cholesterol sequestration from the plasma membrane using 2.5 mM MβCD affected to some extent the tACC recorded for KOP and MOP_wt_, but not for MOP_N40D_ (Figure 4C). Most notably, cholesterol depletion reduced the relative amplitude of the second component ((1 − *y*); Figure 4C, Table S1).

Opioid receptor reorganization upon membrane cholesterol depletion with MβCD was also observed using PC‐PALM (Figure 4D). Cholesterol sequestration from the plasma membrane resulted in reduced average receptor densities with respect to the steady state showing a decrease from (52 ± 4) to (36 ± 2) detected molecules/μm^2^ for MOP_wt_ (*P* < .001) and a decrease from (51 ± 4) to (32 ± 3) detected molecules/μm^2^ for KOP (*P* < .001). Regarding nanoscale organization, a large fraction of receptors had a random distribution (~33% of analyzed areas for MOP_wt_ and ~57% for KOP). The fraction of receptors that showed nonrandom organization had a complex behavior, with domains containing fewer proteins compared to the steady state. A similar effect was previously observed for the lipid raft marker GPI after cholesterol sequestration, wherein a large fraction of regions exhibited a random distribution.^14^ These results further suggest that opioid receptors may be partially associated with cholesterol‐enriched domains where they can engage in signaling platform activities.

### 3.3 Opioid receptors are largely excluded from GM1 ganglioside‐enriched domains

**Figure 5:**
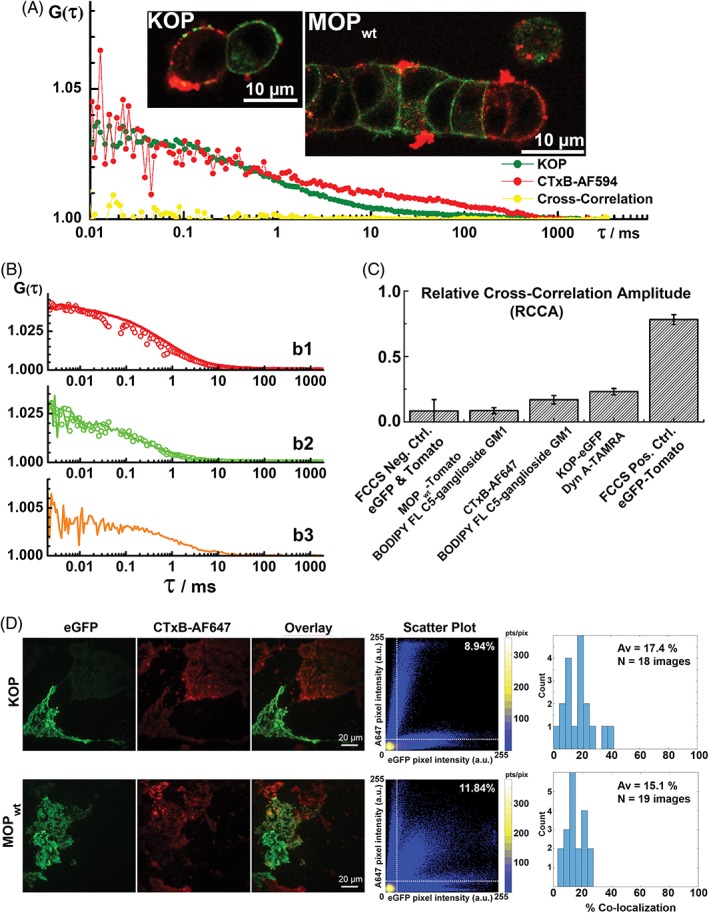
Opioid receptors are largely excluded from GM1‐enriched domains in live stably transformed PC12 cells and in COS‐7 cells. (A) CLSM imaging shows very limited KOP‐eGFP (left inset, green) or MOP_wt_‐eGFP (right inset, green) colocalization with GM1 ganglioside‐enriched domains visualized using CTxB‐AF594 (red). FCCS shows that in regions where colocalization was observed, there is no cross‐correlation (yellow) between the opioid receptor (here KOP) related signal (green) and the CTxB‐AF594 (red). (B) b1: tACC of CTxB‐AF647 recorded in the red channel using continuous excitation in both channels simultaneously (red line) vs alternating excitation (red circles). b2: tACC of BODIPY FL C_5_‐ganglioside GM1 recorded in the green channel using continuous excitation in both channels simultaneously (green line) vs alternating excitation (green circles). b3: tCCC recorded using continuous excitation in both channels simultaneously (orange line). (C) The degree of association between different molecules assessed from the relative cross‐correlation amplitude (RCCA), that is, the amplitude of the tCCC relative to the amplitude of the tACC in the green channel (*A*_CC_/*A*_AC,green_). The relative cross‐correlation amplitude below (0.10 ± 0.08) indicates no binding (FCCS negative control, eGFP and Tomato), whereas the relative cross‐correlation amplitude of (0.75 ± 0.04) indicates full binding (eGFP‐Tomato‐linked dimer). The RCCA amplitude for BODIPY FL C_5_‐ganglioside GM1 binding to CTxB‐AF647 (0.17 ± 0.04) is significantly different from the negative control, as evident from the 2‐tail *P*‐value: *P* < .03. (D) TIRF images showing the spatial distribution of KOP‐eGFP (green) and GM1 ganglioside‐enriched domains visualized using CTxB‐AF647 (red) in COS‐7 cells (top row); and MOP_wt_‐eGFP (green) and GM1 ganglioside‐enriched domains visualized using CTxB‐AF647 (red) in COS‐7 cells (bottom row). Scatter plots of red and green pixel intensities show limited colocalization. Pixel intensity scatter plots are prepared from corresponding red (CTxB‐A647) and green (KOP/MOP‐eGFP) images. The density of points in each scatter plot is indicated by a color bar. Additionally, the white dotted lines define a cut‐off for 10% of the maximum intensities from the 2 images, and the percentage of pixels residing within the upper right regions is indicated. Similar approach for characterizing colocalization has been used previously^77^

Cholera toxin subunit B conjugated with the fluorescent markers Alexa Fluor 594 or Alexa Fluor 647 (CTxB‐AF594 and CTxB‐AF647, respectively) were used to visualize GM1 ganglioside‐enriched domains in opioid receptor transfected PC12 and COS‐7 cells (Figure 5). We first probed opioid receptor association with the GM1 ganglioside‐enriched domains by CLSM/FCCS in live stably transformed PC12 cells (Figure 5A). CLSM imaging suggested that colocalization is minimal between opioid receptor‐rich domains and GM1‐rich domains detected with CTxB‐AF594, and FCCS performed in cells where colocalization was observed showed no appreciable cross‐correlation (Figure 5A, yellow).

In order to ascertain that lack of cross‐correlation is not an artifact arising due to fluorescence labeling, this finding was further scrutinized using BODIPY FL C5‐ganglioside GM1 to visualize endogenous GM1 ganglioside‐enriched domains in PC12 cells stably expressing MOP_wt_‐Tomato (Figure S7). In line with previous observations, these experiments showed that while BODIPY FL C5‐ganglioside GM1 readily binds to the majority of cells, no appreciable cross‐correlation between GM1 and MOP is seen (Figure S7B, yellow). Finally, we have verified by FCCS that fluorescently labeled CTxB‐AF647 and BODIPY FL C5‐ganglioside GM1 (Figure 5B, b1‐b3) bind and have calculated the extent of cross‐correlation for this complex (Figure 5C). Detailed discussion is given in Appendix S1.

Likewise, TIRF microscopy indicated that CTxB‐AF647 readily binds to COS‐7 cells expressing opioid receptors at low levels, whereas it is largely excluded from cells expressing opioid receptors at high levels (Figure 5D). Consequently, in a majority of cells, colocalization between KOP or MOP_wt_ and GM1‐rich domains is limited. The percentage of colocalizing pixels was 15% to 17% on average, for all analyzed images (Figure 5D). Together, our data suggest that opioid receptors are largely excluded from GM1 ganglioside‐enriched domains.

## 4 DISCUSSION

Compartmentalization is a fundamental feature of cellular plasma membranes, with profound consequences on how cells perceive information from their surroundings and relay this information to the cell interior. Compartmentalization allows physical separation of molecules from their immediate surroundings and their accumulation in/exclusion from confined areas, thereby altering their local concentration, kinetics of interaction with other molecules and chemical reaction equilibria. To better understand opioid receptor compartmentalization in the plasma membrane, we used 2 techniques with single molecule sensitivity, FCS and PALM, to quantitatively characterize the lateral dynamics and spatial organization of opioid receptors KOP, MOP_wt_ and MOP_N40D_. These opioid receptors show important similarities but also subtle differences in their overall tertiary three‐dimensional (3D) configurations.^78^

Multiple controls were performed to validate the approach by FCS and PALM. In particular, fluorescently tagged opioid receptor function in PC12 and COS‐7 cells was evaluated in detail using agonist stimulation (Figures S1‐S3). Furthermore, care was taken to use optimal conditions to detect opioid receptors by FCS and PALM, and technical aspects were assessed using model systems and simulations (Figures S4‐S10). Finally, we verified in a key control experiment that the location of the fluorescent protein tag did not significantly affect the observed results (Figure S11).

Despite all precautions, some inevitable limitations are still present. For example, neither FCS nor PALM can account for endogenous nonfluorescent opioid receptors, opioid receptor constructs with irreversibly photobleached fluorophores or with fluorophores residing for various reasons in dark states. In addition, FCS cannot account for proteins associated with large immobile structures, which contribute to the overall background signal but do not give rise to fluorescence intensity fluctuations. As a consequence, the receptor surface density can be in general somewhat underestimated by both FCS and PALM.^60^, ^79^ In contrast, the number of opioid receptors may also be overestimated by both methods. In PALM, recurrent detection of the same fluorophore due to blinking may result in an overestimation of the number of opioid receptors detected. In order to prevent overcounting, we have evaluated the average number of appearances per molecule and incorporated this in our analysis (see Supplementary Information for details and Figure S8 for validation with model proteins). In FCS, high background signal when compared to fluorescence intensity may lead to an artificially low amplitude of tACCs, and, hence, overestimation of molecular numbers. To minimize this risk, we have performed FCS measurements above the nearly transparent cell nucleus, where the contribution of background fluorescence from the cytoplasm is minimal (Figure 2C). Additionally, we adjusted the laser power so that the detected number of photons per eGFP molecule per second (so‐called counts per second and per molecule) was 1 to 5 kHz. This ensured that the measured signal came from eGFP, rather than from autofluorescence, without inducing extensive photobleaching (Figure S4). In FCS, photobleaching of fluorophores may induce errors in the measurements of molecular numbers and lateral diffusion, yielding both a smaller number of molecules and shorter values of *τ*_D_, and hence apparently larger diffusion coefficients. To avoid artifacts due to photobleaching in FCS measurements, the incident laser intensity was kept as low as possible, but sufficiently high to allow for a good signal‐to‐noise ratio. We similarly optimized PALM imaging parameters, and the densities of opioid receptors reported here match well with expected GPCR densities, and they are consistent with previously reported values using super‐resolution microscopy.^80^ Moreover, the use of fluorescent tags on, for example, opioid receptors, opioid peptide ligands, GM1 and CTxB, alters their binding properties to a different extent. In particular, opioid receptor ligand binding and/or signaling properties may be altered by tagging with fluorescent proteins^81^, ^82^. The fluorescent tag on the opioid receptor peptide ligand β‐endorphin‐TAMRA may affect its binding to the receptor,^83^ possibly a reason why limited internalization of MOP_N40D_ was observed with β‐endorphin‐TAMRA (Figure S2F) while previous reports show that β‐endorphin readily internalizes MOP_N40D_ 84. Photophysical properties and emission of the fluorescent tag BODIPY depend strongly on the total lipid packing density^85^ and the fluorescent tag BODIPY can influence GM1 partitioning in the plasma membrane, thus affecting its interactions with CTxB.^86^ Moreover, CTxB interactions with GM1 ganglioside are notoriously complex and their binding constant is affected by a number of parameters, including the concentration of soluble GM1 in the cell culture medium.^86^, ^87^, ^88^, ^89^ While we discuss some of these limitations in relation to CTxB interactions with GM1 ganglioside in Appendix S1, the results of fluorescence labeling assays should always be carefully interpreted since a number of factors may influence fluorescence intensity.

With this in mind, we have complemented the FCS and PC‐PALM analyses with MC simulations. Experimental and theoretical studies revealed that opioid receptor lateral organization is dynamic and complex. It is characterized by both a distribution of timescales and short‐ and long‐range spatial organization. In particular, our study indicates that: (1) opioid receptors dynamically partition between compartments in the plasma membrane and a fraction of opioid receptor molecules associate with domains whereas the remaining molecules are embedded in the surrounding lipid bilayer (Figures 2 and 3); (2) the number of opioid receptors in nanodomains, the nanodomain size and fraction of proteins residing in nanodomains were largest for KOP and smallest for MOP_N40D_ (Figures 2 and 3; Table 1); (3) opioid receptor organization within domains is not random (Figures 3 and S9); (4) MOP_wt_ and KOP are partially associated with cholesterol‐enriched domains and partition between the domains and the lipid bilayer in relative proportion to cholesterol abundance in the plasma membrane, while this is not observed for MOP_N40D_ (Figure 4) and (5) the studied opioid receptors are largely excluded from GM1 ganglioside‐enriched domains (Figure 5).

**Figure 6:**
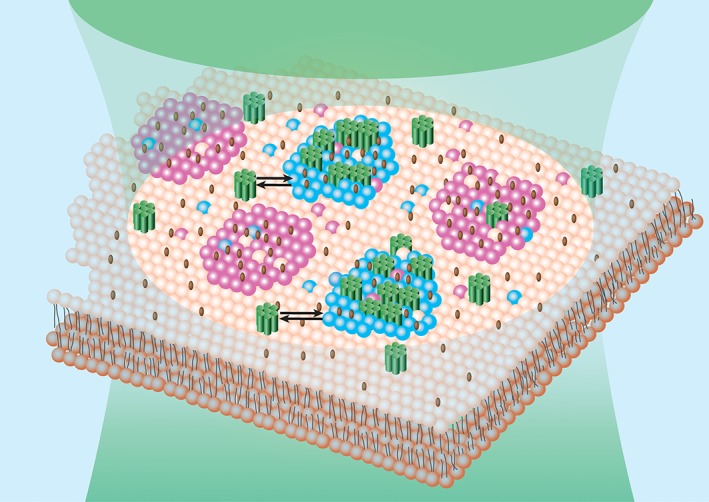
Complex lateral organization of opioid receptors at the nanoscale level. Schematic presentation of dynamic lateral organization of opioid receptors (green cylinders) in the plasma membrane lipid bilayer (pale orange). Magenta regions indicate GM1‐enriched domains from which opioid receptors are largely excluded. Cyan regions indicate domains enriched in cholesterol (brown rods), with which opioid receptors partially associate. Arrows indicate that opioid receptor partitioning between the nanodomains and the lipid bilayer, and their number in each phase is dynamically regulated. In the case of KOP and MOP_wt_, the fraction of molecules associated with nanoscale domains decreases when the cholesterol content in the plasma membrane is depleted

Based on these data, the data obtained in our previous work,^48^, ^71^ and the notion that chemical interactions can influence the apparent diffusion behavior of molecules,^90^ we propose a model to explain the organization of opioid receptors within the plasma membrane. According to this model, a fraction of opioid receptors (Figure 6, green) is associated with cholesterol and GPI‐enriched domains (Figure 6, cyan). The remaining opioid receptors are distributed throughout the phospholipid bilayer (Figure 6, orange) and largely excluded from GM1 ganglioside‐enriched domains (Figure 6, magenta). Protein‐ and lipid‐enriched domains in the plasma membrane are not static physical entities, but in fact dynamic structures that continuously form and dissipate over the lifetime of a cell. At any given time, a finite number of nanodomains exist and a fraction of the total opioid receptor population is associated with these domains. The actual proportion of opioid receptors associated with domains is receptor‐specific—KOP has the largest and MOP_N40D_ has the smallest fraction of receptors that are located inside nanodomains. Taken together, the opioid receptors appear to have different spatial organizations within the lipid bilayer.

Observed differences between organization of MOP and MOP_N40D_ could in part be due to posttranslational modifications. Glycans and glycosylation play a prominent role in plasma membrane lateral organization. The intensity of interactions that maintain protein assemblies at the cell surface can be dynamically modulated by altering protein glycosylation.^91^ The SNP A118G abolishes the N‐glycosylation site in MOP,^92^, ^93^ thereby reducing the strength of cohesive interactions in MOP_N40D_. This is reflected in FCS as a decrease in the relative contribution of the slow tACC component (Figure 2B,C), and in PALM as an increase in the fraction of receptors that reside outside the domains (Figure 3G). Detected density of MOP_N40D_ was lower compared to MOP_wt_ in both cell lines (Figures 2F and S3), in line with previous observations.^84^ While surface density can influence the distribution of proteins,^94^, ^95^, ^96^, ^97^ loss of the N‐glycosylation site is likely to drive this effect in the case of MOP_N40D_. While a specific, cholesterol‐enriched environment appears to be an important factor in opioid receptor lateral organization, the abundance of the freely diffusing component was insensitive to plasma membrane cholesterol content for MOP_N40D_ (Figure 4C).

Differences in lateral organization of opioid receptors could also be due to the local lipid environment and mechanisms of cholesterol regulation.^98^ Cholesterol is a vital constituent of the plasma membrane, known to modulate the function of many GPCRs by direct interaction or by altering local properties of the plasma membrane.^46^, ^52^, ^98^, ^99^ The 2 proposed mechanisms are not mutually exclusive and both may be occurring in cells (reviewed recently in Reference 98). As suggested by both FCS and PC‐PALM measurements, KOP nanodomains are most populated and most sensitive to cholesterol sequestration from the plasma membrane. The finding that cholesterol sequestration distinctly affects KOP, MOP_wt_ and MOP_N40D_ is novel but not entirely surprising, as it was previously shown that MOP and DOP (delta opioid receptor) signaling to adenylyl cyclase is differently affected by cholesterol sequestration from the plasma membrane.^100^

Finally, observed differences in nano‐organization of opioid receptors could also be due to (1) specific associations with cytoskeletal elements^46^, ^101^ and (2) their local protein environment (eg, availability of interacting proteins^102^).

Irrespective of how the lateral organization of opioid receptors (or other GPCRs) is brought about and maintained in live cells, enrichment of plasma membrane proteins in multi‐protein assemblies may be a fundamental principle with important functional implications for lateral transfer of information in the plasma membrane and for signal transduction across the plasma membrane.^46^, ^52^, ^103^, ^104^, ^105^, ^106^ Recently, Halls et al^52^ have shown that MOP_wt_ lateral organization is subject to distinct changes upon stimulation with specific agonists. They have also shown that lateral organization is a relevant determinant of MOP_wt_ function, as it gives rise to distinct ligand‐induced spatiotemporal signaling profiles.^52^ Our observation that lateral organization of MOP_wt_ is dynamic in unstimulated cells is in line with the finding by Halls et al.^52^ Importantly, our results suggest that other investigated opioid receptors also have dynamic and distinct lateral organization in unstimulated cells. Dynamic lateral organization of opioid receptor molecules and their sorting between nanodomains may be an efficient way to fine‐tune their surface density in unstimulated cells. Receptor synthesis and trafficking are slow processes, which take place at the minute and hour time scales. In contrast, plasma membrane domain sorting is significantly faster, with sub‐second time scales. By transiently sorting opioid receptors to and from nanodomains, the number of functional units at the cell surface can be swiftly altered without the need to change the total number of receptor molecules in the cell, which would require the activation of slow processes such as cellular trafficking and receptor synthesis.

The results reported here were obtained using 2 techniques with single‐molecule sensitivity in 2 cell lines (FCS was performed on the apical membrane of live PC12 cells while PC‐PALM was performed on the basal membrane of fixed COS‐7 cells). Despite experimental differences, congruent results were obtained. For example, both FCS (Figure 2C) and PC‐PALM show that the MOP_N40D_ surface density is lower than the MOP_wt_ surface density in both cell lines. These results are also in agreement with literature reporting on other cell systems and at the organism level. At the organism level, studies consistently show that natural and heterologous expression of the MOP_N40D_ variant is lower, both at the mRNA and protein levels.^92^, ^107^ Moreover, FCS and PC‐PALM (with the latter supported by MC simulations) concomitantly showed that the fraction of receptors associated with plasma membrane domains was largest for KOP and smallest for MOP_N40D_. Different cell lines lead to different absolute values, but consistent trends have been seen across the receptors.

## 5 CONCLUDING REMARKS

In summary, our results provide the first detailed view on the dynamic lateral organization of 3 distinct opioid receptors in the plasma membrane. Our study shows that opioid receptors organize largely within nanodomains; they show distinct sensitivities to cholesterol sequestration from the plasma membrane and they are largely excluded from GM1‐enriched domains in the cell lines investigated here. Furthermore, the studied opioid receptors subtly differ in their lateral organization at the nanoscale level. For example, the largest and most populated domains were found for KOP, whereas the smallest and least populated domains were found for MOP_N40D_. Our data also suggest that cholesterol is a major determinant of KOP and MOP_wt_ lateral organization and that cholesterol sequestration perturbs the integrity of lipid raft domains harboring KOP and MOP_wt_ receptors. The extent of this effect was not observed for MOP_N40D_. More studies at the nanoscale level are needed to characterize the functional role of these hitherto unobserved differences. Specifically, these will be important for shedding new light on the dynamic regulation of opioid receptor lateral organization by other plasma membrane constituents besides cholesterol, the perturbation of receptor organization in disease states, and the effects of pharmacological substances. Advancing our understanding of these effects is critical because they involve a dynamic regulatory mechanism that does not affect opioid receptor function by acting directly on the orthosteric binding site. Allosteric modulation of GPCR function is of paramount importance for drug discovery.^108^ Quantitative methods are needed to characterize the capacity of drug candidates to fine‐tune receptor functions by utilizing this dynamic regulatory mechanism. Our work represents a significant step forward in this direction. Correlating PC‐PALM results with FCS, we show how the dynamic lateral organization of cell surface receptors can be effectively and nonintrusively characterized in great detail.

## 6 MATERIALS AND METHODS

### 6.1 DNA constructs

Plasmids encoding MOP_wt_‐eGFP, MOP_N40D_‐eGFP and KOP‐eGFP in an *N*_1_ vector were designed as previously described.^48^ Plasmids encoding MOPwt‐paGFP, MOP_N40D_‐paGFP and KOP‐paGFP in an *N*_1_ vector were generated by exchanging eGFP in the corresponding opioid receptor‐eGFP constructs with paGFP using AgeI/NotI restriction enzyme sites. Plasmids encoding N‐terminally tagged GFP‐MOP_wt_ and GFP‐KOP were kindly obtained from Brilliant BioSciences and used in control experiments to assess whether fluorescent protein tag location affects opioid receptor lateral dynamics.

### 6.2 Cell culture, transfection, vital cell staining and immunoblotting

PC12, COS‐7, PANC‐1, MCF‐7 and MDA‐MB‐468 cells were purchased from the American Type Culture Collection.

PC12 cells stably transformed to express opioid receptors C‐terminally tagged with eGFP were cultured in collagen‐coated flasks using RPMI 1640 medium supplemented with 5% FBS, 10% heat inactivated horse serum, 100 U/mL penicillin and 100 mg/mL streptomycin (all from Invitrogen). They were maintained at 37°C in a humidified 5% CO_2_ incubator. For FCS/FCCS experiments, the cells were plated on 8‐well Nunc Lab‐Tek Chambered Coverglass with a 1.0 borosilicate bottom (Thermo Fisher Scientific) 2 to 3 days before the experiment. Cells were grown in phenol red‐free RPMI medium supplemented with 10% horse serum, 5% FBS, penicillin (100 units/mL) and streptomycin (100 mg/mL) in a humidified 5% CO_2_ atmosphere at 37°C.

For cholesterol depletion experiments, stably transformed PC12 cells were incubated for 3 hours with 2.5 mM MβCD (Sigma) in a serum‐free medium, in a humidified 5% CO_2_ atmosphere at 37°C. To visualize cholesterol, PC12 cells in culture (total volume of 250 μL) were incubated for 10 minutes with 3 μL cholesteryl BODIPY (542/563 C_11_; MolecularProbes, Thermo Fisher Scientific). To visualize GM1 ganglioside‐enriched domains in stably transformed PC12 cells, the standard growth medium was removed from cells grown in 8‐well chambered coverglass and the cells were incubated for 10 minutes at room temperature with refrigerated RPMI phenol red‐free medium supplemented with 10% horse serum, 5% FBS and 1% PenStrep and augmented with 1 μg/mL Cholera Toxin Subunit B (Recombinant) Alexa Fluor 594 or Alexa Fluor 647 Conjugate (MolecularProbes, Thermo Fisher Scientific). BODIPY FL C_5_‐ganglioside GM1 was used in control experiments to characterize its association with CTxB Alexa Fluor conjugates. Stock solutions of BODIPY FL C_5_‐ganglioside GM1 and CTxB Alexa Fluor conjugates were prepared by suspending a scaled amount of the powder in 200 μL of PBS with 10% DMSO. Stock solution of 50 μL was diluted further in 300 μL PBS and the concentration was established by FCS.

COS‐7, PANC‐1, MCF‐7 and MDA‐MB‐468 cells were cultured in phenol red‐free Dulbecco's modified eagle medium (DMEM) supplemented with 10% FBS, 1 mM sodium pyruvate, 100 units/mL penicillin, 100 units/mL streptomycin and 2 mM l‐alanyl‐l‐glutamine (full DMEM). GPI and VSVG tagged with paGFP (paGFP‐GPI and VSVG‐paGFP constructs, respectively) were transiently transfected similarly as described before.^71^ Following manufacturer's instructions, 3 μg of opioid receptor constructs tagged with paGFP were transiently transfected in COS‐7 cells using Jetprime (PolyPlus) transfection reagent. Approximately, 24 to 36 hours (GPI and VSVG constructs) and 48 hours (opioid receptors) after transfection, cells were fixed as described before.^71^

PALM cholesterol depletion experiments were conducted in COS‐7 cells transfected with either MOP_wt_‐paGFP or KOP‐paGFP. Forty‐eight hours posttransfection, cells were incubated with 10 mM MβCD (Sigma) in full DMEM supplemented with 10 mM HEPES and 1 mg/mL BSA for 30 minutes at 37°C, similarly as described before.^14^ Cells were rinsed 3 times with PBS and fixed as described previously.^71^

For TIRF measurements, COS‐7 cells were transiently transfected with KOP‐eGFP or MOP‐eGFP constructs. Approximately, 48 hours posttransfection, cells were incubated with 18 nM CTxB‐AF647 for 15 minutes at 37°C and fixed.

### 6.3 Optical setup for CLSM and FCS

CLSM imaging and FCS were performed under controlled temperature (37°C) and atmosphere (5% CO_2_ in humidified air) using a LSM 510 ConfoCor 3 system (Carl Zeiss) individually modified to enable the use of avalanche photodiodes (APDs; SPCMAQR‐1X; Perkin‐Elmer) for imaging. These detectors are characterized by higher sensitivity and lower noise levels, making it possible to visualize by imaging fluorescently labeled molecules at nanomolar concentrations.^109^ eGFP and BODIPY FL C_5_‐ganglioside GM1 were excited using the 488 nm line of the Ar‐ion laser; TAMRA, Tomato and CTxB Alexa Fluor 594 were excited using the HeNe 543 nm laser; and Alexa Fluor 647 was excited using the HeNe 633 nm laser. For dual color imaging and FCCS, the HFT 488/543/633 main dichroic beam splitter was used to separate the incident and emitted light. eGFP fluorescence was transmitted to the detector through a band‐pass filter BP 505‐530; a band‐pass filter BP 560‐610 was used for TAMRA and Tomato; and a long‐pass filter LP 650 was used for Alexa Fluor 594 or Alexa Fluor 647. Images were acquired using the C‐Apochromat ×40 NA = 1.2 water immersion UV‐VIS‐IR objective, scanning speed of 25.6 μs/pixel, without averaging and 512 × 512 pixel resolution.

FCS and FCCS measurements were performed using the same optical pathway that was used for imaging (described above). Fluorescence intensity fluctuations were recorded without pre‐bleaching, in a series of 10 consecutive measurements, each measurement lasting 10 seconds. Temporal autocorrelation analysis was used to analyze the fluorescence intensity fluctuations and determine the concentration and the diffusion time of the investigated species. Since great care was taken to minimize photobleaching, we could typically analyze all 10 traces by temporal autocorrelation analysis (Figure S4). Occasionally, however, the first measurement was obviously different from the remaining 9 tACCs collected in one series and was not considered in the averaging. The optical setup and control experiments are described in detail in Appendix S1: FCS and FCCS.

### 6.4 Fitting temporal autocorrelation curves

Fitting of tACCs was performed using the dedicated Zeiss software. As described in detail in Appendix S1: FCS and FCCS, the simplest model that could account for the tACC recorded at the cellular plasma membrane is a model for free two‐dimensional (2D) diffusion of 2 components with intersystem crossing:

(4) $$G\left(\tau \right)=1+\frac{1}{N}\left(\frac{y}{\left(1+\frac{\tau }{\tau_{D1}}\right)}+\frac{1-y}{\left(1+\frac{\tau }{\tau_{D2}}\right)}\right)\cdot \left[1+\frac{T}{1-T}\exp\left(-\frac{\tau }{\tau_{T}}\right)\right].$$

Here, *N* is the average number of molecules in the OVE; *τ*_D1_ is the shorter and *τ*_D2_ the longer diffusion time; *y* is the fraction of opioid receptor molecules with the shorter diffusion time *τ*_D1_; (1 − *y*) is the fraction of opioid receptor molecules with the longer diffusion time *τ*_D2_; *T* is the average equilibrium fraction of molecules in the triplet state and *τ*_T_ the triplet correlation time, related to the rate constants for intersystem crossing and triplet decay. In all measurements the triplet state occupancy was <20%.

### 6.5 Optical setup and preparatory procedures for super‐resolution PALM and TIRF imaging

PALM imaging was performed on a 3D N‐STORM super‐resolution microscope (Nikon). The N‐STORM system (Nikon Instruments) consists of a fully automatic Ti‐E inverted microscope with piezo stage on a vibration isolation table. This system includes a ×100 1.49 NA TIRF objective (Apo); an N‐STORM lens and *λ*/4 plate; and Quad cube C‐NSTORM (97 355 Chroma). The microscope has a Perfect Focus Motor to maintain imaging at the desired focal plane; an MLC‐MBP‐ND laser launch with 405, 488, 561 and 647 nm lasers (Agilent); and an EM‐CCD camera iXon DU897‐Ultra (Andor Technology). Cells were grown on clean coverslips coated with fibronectin‐like engineered protein as described before.^71^

Images of a 27.3 μm × 27.3 μm area were collected with an exposure time of 100 ms using the software Andor SOLIS for Imaging X‐07779 (Andor Technology); pixel size was 106.7 nm. paGFP, an excellent monomeric optical highlighter protein with good signal‐to‐noise ratio, was simultaneously activated and excited using the 488 nm laser with the power set within the range of 1.45 to 1.9 mW (measured at the optical fiber). Imaging was done until paGFP was completely exhausted, typically 20,000 frames. TetraSpeck beads (Life Technologies) were used as fiducial markers for drift‐correction during PALM acquisition.

TIRF imaging was performed on the same microscope system described herein for PALM; ×60 1.49 NA TIRF objective (Apo) was used. Images of 512 × 512 pixels were collected using NIS Elements 4.3 Software (Nikon); eGFP was excited using the 488 nm laser and Alexa Fluor 647 was excited using 647 nm laser. Overlay images were constructed using Matlab.

### 6.6 Fitting spatial autocorrelation curves

PALM image analysis was performed as described in Appendix S1 and in References 14, 15 and 71. Densities of proteins were calculated from areas of 7 to 18 μm^2^. Average detected densities with SEM were reported. Subsequently, auto‐correlation functions were calculated for each area independently to estimate protein organization parameters. As demonstrated previously^71^ and detailed in Figure S8, protein organization can be inferred from averaged autocorrelation curves. Mathematical equations related to the single exponential model of the auto‐correlation function *g*(*r*)^protein^ can be found in References 14, 15. The following equation, which is a combination of Gaussian and exponential function, is utilized to account for the complex spatial organization of opioid receptors, that is, the complex shape of the experimentally obtained sACC *g*(*r*)^protein^:

(5) $$g{\left(r\right)}^{\text{protein}}=A_{1}e^{\frac{-{\left(r-{\text{peak}}_{\mathrm{MAX}}\right)}^{2}}{2B^{2}}}+A_{2}e^{\frac{-r}{\xi_{D}}}+1.$$

Here, the first component of the equation (Gaussian) describes short‐range interactions, while the second component (exponential) describes long‐range interactions. The variable *r* is the radius in nm. The constant peak_MAX_ is the maximum value of the auto‐correlation function. We estimated peak_MAX_ from the average auto‐correlation function computed for experimental data: the value of the peak_MAX_ was iteratively changed until we reached fitting optimization (as confirmed by MC simulations). We used this value to fit the equation. *A*_1_, *A*_2_, *B* and *ξ*_D_ are the variables in Equation 5, where *A*_1_ is the short‐range pre‐exponential factor; *A*_2_ is the long‐range (domain) pre‐exponential factor; *B* is the short‐range radius and *ξ*_D_ is the long‐range (domain) radius.

In this way, we report the following parameters related to domain organization as:

(6) $${\text{radius}}^{\text{domain}}\left(\mathrm{nm}\right)=\frac{1}{A_{2}}\int_{0}^{\infty }A_{2}e^{\frac{-r}{\xi_{D}}}\mathrm{dr}=\xi_{D},$$

(7) $$N^{\text{domain}}≅\rho_{\mathrm{avg}}\int_{{\text{peak}}_{\text{MAX}}}^{\infty }A_{1}e^{\frac{-{\left(r-{\text{peak}}_{\mathrm{MAX}}\right)}^{2}}{2B^{2}}}\left(2\mathrm{\pi r}\right)\mathrm{dr}+\rho_{\mathrm{avg}}\int_{0}^{\infty }A_{2}e^{\frac{-r}{\xi_{D}}}\left(2\mathrm{\pi r}\right)\mathrm{dr}≅\rho_{\mathrm{avg}}2\pi \left(A_{1}B^{2}+A_{2}\xi_{D}^{2}\right),$$

where *ρ*_avg_ is the average density of molecules in the analyzed region of interest and *N* ^domain^ is the number of detected proteins per domain. The increased local density in a domain (density in the domain compared to average cell density), *ψ* ^domain^ can be estimated using the following equation:

(8) $$\psi^{\text{domain}}=\frac{\rho^{\text{domain}}}{\rho_{\mathrm{avg}}}=\frac{N^{\text{domain}}}{{\pi \xi }_{D}^{2}\rho_{\mathrm{avg}}}=\frac{2\left(A_{1}B^{2}+A_{2}\xi_{D}^{2}\right)}{\xi_{D}^{2}},$$

where *ρ* ^domain^ is the density of molecules in a domain. Thus, both domain density and cell density influence *ψ* ^domain^ value. The average auto‐correlation functions for opioid‐receptor‐paGFP constructs were fitted using the complex model; results are shown in Tables 1 and S2. All fittings resulted in *R* ^2^ ≥ 0.98.

### 6.7 Clustering algorithm to identify domains

PALM datasets were used to determine the fraction of proteins residing outside domains. The domains are recognized by a clustering algorithm (code written in Matlab) which utilizes the following: spatial coordinates of peaks, number of photons, the localization precision of the peaks, the blinking time of paGFP, and the domain radius calculated using the complex fitting. The clustering algorithm proceeds in 2 steps:

1. Identifying real detected proteins without artifacts coming from multiple appearances of peaks. Multiple peaks are assigned to one protein if they satisfy the 2 conditions, 1 spatial and 1 temporal. Peaks that appear within a resolution‐limited spot and within a temporal window equal to the paGFP maximum blinking time are assigned to the same protein. If one of these conditions is not satisfied, peaks are assigned to a new protein.
2. Identifying domains of proteins. Proteins are assigned to a domain if the radial distance between the protein and the center of the domain is less than or equal to the domain radius. If the number of proteins inside the confined space within the domain radius is equal to or greater than 3, then the above proteins belong to a domain. If the number is less than 3, then the proteins are considered free floaters. The shapes of the domains are assumed to be circular with a known maximum domain radius, obtained from fitting (Table 1). Since the number of domains is unknown a priori, a flexible clustering algorithm was employed. Here the DP‐means algorithm^110^ similar to the standard *k*‐means algorithm was used: a new domain was created when a point was farther than a distance DR away from the centers of existing domains.

### 6.8 Statistical analysis

Paired sample *t* test statistical analysis was performed in Excel or Matlab. Matlab built‐in function “vartest2” was used to calculate the *F* test statistics. The null hypothesis is accepted if the variances of 2 populations are equal within 5%. SEs of the estimate (*S* values) were calculated using Matlab.^111^

## Supporting information


**Editorial Process**

**Appendix S1** Supplementary methods and References.
**Table S1** Comparison between tACC for KOP, MOP_wt_ and MOP_N40D_ across different experiments.
**Table S2** *F* test and *S* values between correlation curves reported in Figures 3B,D,F; 4D S9D, and S9E. Matlab built‐in function “vartest2” was used to calculate the *F* test statistics and this value was subsequently compared to the *F*‐critical value based on the number of data points (*F*‐critical = 1.2172; No. of observations: 282). The null hypothesis is accepted if the variances of 2 populations are equal within 5%. SEs of the estimate (*S* values) were calculated using Matlab (46). PPD, protein per domain.
**Figure S1** CLSM images of live PC12 cells stably transformed to express different opioid receptors. (A) KOP‐eGFP; (B) MOP_wt_‐eGFP; (C) MOP_N40D_‐eGFP. Scale bar: 10 μm.
**Figure S2** CLSM images of live PC12 cells expressing different opioid receptors before and after stimulation with corresponding specific ligands.
**Figure S3** Expression levels and activities of opioid receptor constructs in COS‐7 cells.
**Figure S4** FCS measurements and fitting analysis of experimentally derived temporal autocorrelation curves (tACC) for MOP_wt_‐eGFP.
**Figure S5** Experimental validation of FCS data interpretation by fitting analysis.
**Figure S6** Circumventing spectral cross‐talk in dual‐color FCCS by alternating excitation.
**Figure S7** Dual‐color FCCS analysis of MOPwt‐Tomato interactions with BODIPY FL C5‐ganglioside GM1.
**Figure S8** paGFP‐GPI and VSVG‐paGFP molecular distribution.
**Figure S9** Monte Carlo simulations without organized nanodomains do not resemble experimental results.
**Figure S10** Monte Carlo simulations are robust to different random seeds. Consistent results are obtained for all opioid receptors: (A) KOP; (B) MOP_wt_ and (C) MOP_N40D_.
**Figure S11** Opioid receptor lateral dynamics in the plasma membrane is not significantly altered by fluorescent protein tag localization.
