# Supplementary material for: Dynamic lateral organization of opioid receptors (kappa, muwt and muN40D) in the plasma membrane at the nanoscale level
Source: Traffic. 2018 Jun 21;19(9):690–709. doi: 10.1111/tra.12582 (PMC6120469; doi:10.1111/tra.12582)
Supplement: Supplementary file 2 — Appendix S1 Supplementary methods and References. Table S1 Comparison between tACC for KOP, MOPwt and MOPN40D across different experiments. Table S2 F test and S values between correlation curves reported in Figures 3B,D,F; 4D S9D, and S9E. Matlab built‐in function “vartest2” was used to calculate the F test statistics and this value was subsequently compared to the F‐critical value based on the number of data points (F‐critical = 1.2172; No. of observations: 282). The null hypothesis is accepted if the variances of 2 populations are equal within 5%. SEs of the estimate (S values) were calculated using Matlab (46). PPD, protein per domain. Figure S1 CLSM images of live PC12 cells stably transformed to express different opioid receptors. (A) KOP‐eGFP; (B) MOPwt‐eGFP; (C) MOPN40D‐eGFP. Scale bar: 10 μm. Figure S2 CLSM images of live PC12 cells expressing different opioid receptors before and after stimulation with corresponding specific ligands. Figure S3 Expression levels and activities of opioid receptor constructs in COS‐7 cells. Figure S4 FCS measurements and fitting analysis of experimentally derived temporal autocorrelation curves (tACC) for MOPwt‐eGFP. Figure S5 Experimental validation of FCS data interpretation by fitting analysis. Figure S6 Circumventing spectral cross‐talk in dual‐color FCCS by alternating excitation. Figure S7 Dual‐color FCCS analysis of MOPwt‐Tomato interactions with BODIPY FL C5‐ganglioside GM1. Figure S8 paGFP‐GPI and VSVG‐paGFP molecular distribution. Figure S9 Monte Carlo simulations without organized nanodomains do not resemble experimental results. Figure S10 Monte Carlo simulations are robust to different random seeds. Consistent results are obtained for all opioid receptors: (A) KOP; (B) MOPwt and (C) MOPN40D. Figure S11 Opioid receptor lateral dynamics in the plasma membrane is not significantly altered by fluorescent protein tag localization. [file TRA-19-690-s001.docx]

**Dynamic lateral organization of opioid receptors (kappa, mu_wt_ and mu_N40D_) in the plasma membrane at the nanoscale level**

Maciej K. Rogacki,^1,*^,**^∴^** Ottavia Golfetto,^2,*^ Steven J. Tobin,^2^ Tianyi Li,^1^ Sunetra Biswas,^2^ Raphael Jorand,^2^ Huiying Zhang,^2^ Vlad Radoi,^1^ Yu Ming,^1^ Per Svenningsson,^1^ Daniel Ganjali,^3^ Devin L. Wakefield, ^2^ Athanasios Sideris,^3^ Alexander R. Small,^4^ Lars Terenius,^1,5^ Tijana Jovanović-Talisman,^2,#^, Vladana Vukojević^1,#^

^1^Department of Clinical Neuroscience, Karolinska Institutet, Center for Molecular Medicine, CMM L8:01, 17176 Stockholm, Sweden

^2^Beckman Research Institute, City of Hope, 1500 East Duarte Road Duarte, CA 91010, USA

^3^Department of Mechanical and Aerospace Engineering, The Henry Samueli School of Engineering, University of California, Irvine, Irvine, CA 92697

^4^Department of Physics and Astronomy, California State Polytechnic University, Pomona, California 91768, USA

^5^Department of Molecular and Cellular Neurosciences, The Scripps Research Institute, 10550 North Torrey Pines Road, La Jolla, CA 92037, USA

*Equally contributing authors.

**^∴^**Current address: Department of Pharmaceutical Sciences, Medicinal Chemistry, University of Antwerp, 2610 Wilrijk, Belgium

^#^Co-senior, co-corresponding authors.

*Co-corresponding authors:* Tijana Jovanović-Talisman, [ttalisman@coh.org](mailto:ttalisman@coh.org); Vladana Vukojević, [vladana.vukojevic@ki.se](mailto:vladana.vukojevic@ki.se)

Supplementary Methods

**Antibodies**

The antibodies used in the western blot assay have all been well documented and characterized in the antibody profile database Antibodypedia for reactivity in human derived samples. Multiple vendors carry the antibodies used herein; these particular reagent references are listed on the original manufacturers’ website, Pubmed and the aforementioned database.

Primary antibodies used include anti-phospho-AKT (S473) (rabbit polyclonal, Abcam) ([1](#_ENREF_1), [2](#_ENREF_2)); anti-AKT (rabbit monoclonal, Abcam) ([3](#_ENREF_3), [4](#_ENREF_4)); anti-ERK1/2 (rabbit polyclonal, Abcam) ([5](#_ENREF_5), [6](#_ENREF_6)); anti-phospho-ERK1/2 (Thr202/Tyr204) (rabbit monoclonal, Cell Signaling) ([7](#_ENREF_7), [8](#_ENREF_8)); anti-MOR (guinea pig polyclonal, Abcam) ([9-12](#_ENREF_9)); anti-KOR (mouse monoclonal, Neuromics) ([12](#_ENREF_12)); anti-GFP (mouse monoclonal, Abcam) ([13](#_ENREF_13), [14](#_ENREF_14)); and anti-β-actin (mouse monoclonal, Cell signaling) ([15](#_ENREF_15), [16](#_ENREF_16)).

**Extraction of RNA, cDNA synthesis and RT-PCR**

RNA was extracted from cell lines using the RNAeasy Plus Kit (Qiagen) (according to the manufacturer’s specifications). 1 μg of RNA was used to synthesize cDNA using the Bioline cDNA synthesis kit (according to the manufacturer’s protocol). The synthesized cDNAs were used as templates for 1) KOP PCRs using the KOP- forward primer 5′-TGTTTGTCATCATCCGATACAC-3′ and reverse primer 5′-AAACTGCAAGGAGCATTCAATG-3′ ([17](#_ENREF_17)); and 2) MOP PCRs using the MOP- forward primer 5′-TCTGGCTCCAAAGAAAAGGA-3′ and reverse primer 5′-CAATGCAGAAGTGCCAAGAA-3′ ([18](#_ENREF_18)). RT-PCR was performed using 5 x RT Mastermix (Applied Biosystems) on the CFX96 Real time instrument (Biorad). Themocycler conditions were 95 °C for 10 min for 1 cycle; 95 °C for 15 sec; and 60 °C for 1 min for 50 cycles. GAPDH was used as an internal control. CHO cells were used as negative controls and MCF-7 cells as positive controls. Results indicated that COS-7 cells were negative for KOP and MOP gene expression.

**Transient transfection of PC12 cells and plasmids for control FCS/FCCS experiments**

PC12 cells were transiently transfected by lipofection following manufacturer's protocol (Lipofectamine® 2000, ThermoFisher). Prior to transfection, the cell culturing medium was exchanged to serum free Opti-MEM. Otherwise, the cells were treated as described in the Materials and Methods.

*N-terminally labelled opioid receptors:* Plasmids encoding KOP or MOP_wt_ fused with the Green Fluorescent Protein at the N-terminus, GFP-KOP and GFP-MOP_wt_, respectively, were kindly provided by Brilliant BioSciences, FL, USA.

*FCCS negative control:* pEGFP-N1 plasmid was used to express free eGFP. The ptd-Tomato-N1 plasmid designed with two copies of the Tomato coding region linked together to allow intramolecular dimerization, was modified to enable monomeric Tomato expression. Equal amounts of the pEGFP-N1 and the modified ptd-Tomato-N1 plasmids were simultaneously used.

*FCCS positive control:* Nucleotide sequence encoding eGFP was introduced into the modified ptd-Tomato-N1 plasmid and fused to Tomato *via* a linker.

*MOP_wt_-Tomato:* Nucleotide sequence encoding MOP_wt_ was introduced into the modified ptd-Tomato-N1 plasmid and fused to Tomato *via* a linker.

**Validation of opioid receptor functionality by agonist treatment**

To confirm that opioid receptors in stably transformed PC12 cells (Fig. S1) are functional, live PC12 cells were stimulated with dynorphin A (Dyn A) and β-endorphin (β-End) or hepta-enkephalin (hepta-Enk), endogenous peptide ligands for KOP and MOP, respectively ([19](#_ENREF_19)). Peptide ligands fluorescently labeled with carboxytetramethylrhodamine (TAMRA) were custom synthesized and purified > 98% by Biomatik (Wilmington, DE, USA). Time-lapse CLSM imaging was used to capture the dynamics of ligand-induced receptor internalization (Fig. S2).

As expected, Dyn A-TAMRA and β-End-TAMRA induce massive internalization of their corresponding receptors, KOP (Fig. S2 D) and MOP_wt_ (Fig. S2 E), respectively; and a massive simultaneous internalization and co-localization of ligands and receptors in inwards moving transporting vesicles is observed. Stimulation of PC12 cells expressing MOP_N40D_ with β-End-TAMRA causes a pronounced internalization of the ligand, but massive internalization of the receptor or ligand-receptor complexes was not observed (Fig. S2 F). Similarly, hepta-Enk-TAMRA induces massive internalization of the ligand (Fig. S2 G, red), and some internalization of ligand-receptor complexes was also observed (Fig. S2 G, yellow). While it is possible that β-End-TAMRA/hepta-Enk-TAMRA internalization in PC12 cells expressing MOP_N40D_-eGFP may partially occur through endogenous MOP_wt_, which are expressed at very low levels in PC12 cells ([20](#_ENREF_20)), other mechanisms are also possible. In comparison to MOP_wt_, the MOP_N40D_ variant is lacking a putative N-linked glycosylation site in the N-terminus. Altered N-linked glycosylation was shown to affect receptor trafficking to the cell surface, ligand binding affinity, and the ensuing ligand-induced trafficking cascades in many GPCRs. For example, lack of N-glycosylation in human KOP was shown to increase agonist-induced receptor phosphorylation and affect downstream steps in agonist-induced receptor regulation ([21](#_ENREF_21)). Conflicting results are reported in the literature for MOP_wt_ and its naturally occurring and most commonly encountered isoform MOP_N40D_. Findings reported in the classical study by Bond et al. ([22](#_ENREF_22)), showing that these two MOP isoforms differ by about three times in beta-endorphin binding affinity, were contested by Beyer et al. ([23](#_ENREF_23)), showing that there are no differences between them. Kroslak et al. ([24](#_ENREF_24)), on the other hand, have suggested that receptor internalization is a primary rate-limiting step for receptor resensitization and that enhanced resensitization confers apparent resistance to agonist-induced desensitization. Thus, consequences of altered N-linked glycosylation may be multifaceted and the fact that the internalized ligand β-End-TAMRA does not co-localize with the receptor MOP_N40D_-eGFP in trafficking vesicles may reflect such differences occurring *via* rapid receptor recycling, an internalization mechanism that is dominated by transient formation of rapidly dissociating ligand-receptor complexes, followed subsequently by ligand internalization and receptor recycling to the plasma membrane ([25](#_ENREF_25)). Finally, limited internalization of MOP_N40D_ could be due to fluorescent tag on β-End. Significant internalization of MOP_N40D_ observed by Beyer et al. ([23](#_ENREF_23)) was accomplished using β-End without a fluorescent tag. Taken together, the results described above demonstrate that all opioid receptor subtypes are functional in PC12 cells, that KOP and MOP_wt_ show canonical ligand-induced opioid receptor internalization behavior, and that MOP_N40D_ differs in this respect.

To confirm KOP-paGFP, MOP_wt_-paGFP, and MOP_N40D_-paGFP functionality, receptor transfected COS-7 cells were treated with PBS as a vehicle control; 10 nM Dermorphin [Y(D-ALA)FGYPKC; Genscript]; or 2 nM Dynorphin A (Abcam). To prepare protein extracts for immunoblotting, cells were centrifuged and the pellets were washed 2 times in PBS. The cell pellets were then lysed using lysis buffer (150 mM sodium chloride, 50 mM Tris, pH 8.0, 1% NP-40, Protease and Phosphatase Inhibitor Mini Tablets, Pierce) and rotated at 4 °C for 30 min. The cells were next centrifuged for 20 min at 10,000 rpm at 4 °C. The supernatant consisting of the protein lysate was stored at –80 °C. Per standard protocols, SDS-polyacrylamide gel electrophoresis and western blotting were conducted using the lysates. Primary antibodies used included anti-phospho-AKT (S473) (rabbit polyclonal, Abcam); anti-AKT (rabbit polyclonal, Abcam); anti-phospho-ERK1/2 (Thr202/Tyr204) (rabbit monoclonal, Cell Signaling); anti-ERK1/2 (rabbit polyclonal, Abcam); anti-MOR (guinea pig polyclonal, Abcam); anti-KOR (mouse monoclonal, Neuromics); anti-GFP (mouse monoclonal, Abcam); and anti-β-actin (mouse monoclonal, Cell signaling). Proteins were detected with Pierce ECL detection reagents (Pierce). The blots were imaged on film (Fig. S3).

**FCS and FCCS**

FCS and FCCS measurements were performed using the individually modified LSM 510 ConfoCor 3 system (Carl Zeiss, Jena, Germany) described in detail in ([26](#_ENREF_26)). To minimize background signal coming from the cytoplasm, FCS and FCCS measurements were performed at the apical plasma membrane, above the nearly transparent cell nucleus (as described in the main text, Fig. 1A-G) of PC12 cells stably transformed to express opioid receptors tagged with eGFP (Fig. S1). During FCS and FCCS measurements, the cells were maintained under controlled conditions, in humidified 5% CO_2_ atmosphere at 37 °C, using an incubator for the microscope stage consisting of a heating plate (Heating insert P, PeCon GmbH, Erbach, Germany), incubator box (Incubator S, PeCon GmbH), atmosphere controlling device (CTI-Controller 3700, PeCon GmbH), and a temperature controlling device (Tempcontrol 37–2 digital, PeCon GmbH).

For single-color FCS measurements, fluorescence intensity fluctuations were recorded without pre-bleaching on the same spot at the cell surface in a series of 10 consecutive measurements, each measurement lasting 10 s (Fig. S4 A-C). eGFP fluorescence was excited using the 488 nm line of the Ar-ion laser; fluorescence was separated from the incident light using the main dichroic beam splitter HFT KP 700/488 and was passed to the detector through a long-pass filter LP 505. The system was calibrated using a standard solution of Rhodamine 6G (Rh6G; Fig. S4 D). The lateral diffusion time of Rh6G in aqueous solution was measured to be, τ_D, Rh6G_ = (27 ± 3) µs, and using the known diffusion coefficient D_Rh6G_ = (4.14 ± 0.05)×10^-10^ m^2^s^-1^ ([27](#_ENREF_27)) and the relationship τ_D_ = $\omega_{xy}^{2}$/4D_Rh6G_, the e^-2^ radius of the detection volume was determined to be ω_xy_ ~ 210 nm.

For measurements on live cells, the laser intensity at the objective lens was < 10 µW. Under these conditions fluorescence loss due to photobleaching, while barely visible from the very small difference in the mean fluorescence intensity between the first and the last measurement in the same series (Fig. S4 A, top; black *versus* yellow trace) and the overlapping fluorescence intensity fluctuation traces recorded in 10 consecutive measurements (Fig. S4 A, bottom), was revealed by the increasing amplitude of the temporal autocorrelation curves (tACC; Fig. S4 B, top). While inevitable, this minimal level of photobleaching did neither affect the relative contribution of the fast *versus* slow component nor the characteristic decay times, as evident from the well overlapping tACC normalized to the same amplitude (G_n_(τ) = 1 at τ = 10 µs; Fig. S4 B, bottom). And, a robust signal was obtained – the photon count rate per second per eGFP molecule (CPSM) was 1 – 5 kHz. Experiments where CPSM < 1 kHz were not included in the analysis.

Analysis of experimentally obtained tACC by fitting with different autocorrelation functions identified at least three characteristic decay times, of which the shortest decay time, τ < 200 µs, is generated by eGFP-related processes and the two well separated decay times, 200 µs < τ_D1_ < 1 ms and 10 ms < τ_D2_ < 250 ms, are diffusion related and related to opioid receptor lateral dynamics (Fig. S4 C). To assess the validity of this fitting model, control FCS experiments were performed on live cells expressing eGFP in the cytoplasm (Fig. S5 A, violet); opioid receptor lateral diffusion was measured on giant native vesicles that occasionally form in the culture by cell swelling (Fig. S5 A, light magenta); and the size of the observation volume element (OVE) was modified in order to test whether or not the characteristic decay time of the tACC is changing to determine whether the fluctuations in fluorescence intensity are generated by kinetic processes or molecular diffusion (Fig. S5 B-D). These experiments showed that the tACC of eGFP in live cells, KOP-eGFP on native vesicles and KOP-eGFP in live cells, overlap to a large extent at short time lags, τ < 200 µs, while they differ markedly at longer time lags, τ > 200 µs, (Fig. S5 A). The OVE size-scan analysis showed that the shortest decay time is independent of the size of the OVE, as evident from the overlap at short time lags of normalized tACC recorded using OVEs of different size (Fig. S5 C). This suggests that the underlying process is not related to molecular diffusion, but rather to a kinetic process. In contrast, pronounced differences were observed at longer time lags. Most notably, the characteristic decay times were different for different systems examined (Fig. S5 A) and shifted to longer time scales when the OVE size was increased (Fig. S5 C and D). This indicates that the processes that give rise to fluorescence intensity fluctuations at these time scales are diffusion related and arise due to molecular motion. It is interesting to point out that tACC recorded in the plasma membrane of live PC12 cells were markedly different from tACC recorded at the plasma membrane of giant native vesicles (Fig. S5 A, dark green *vs* light magenta). Consistent with measurements on live cells, tACC recorded at the giant native vesicle showed a contribution from processes at the fast timescale, τ < 200 µs. At the longer time scale, however, only one characteristic decay time was observed, τ_D,vesicle_ = (7 ± 1) ms, its value falling between τ_D1_ and τ_D2_ measured at the plasma membrane of live cells (Fig. S5 A). Taken together, the control results summarized in (Fig. S5) verify that the shortest decay time, τ < 200 µs, does not reflect opioid receptor properties, but is rather related to photophysical processes and/or conformational transformations of eGFP ([28](#_ENREF_28)), whereas the other two decay times, 200 µs < τ_D1_ < 1 ms and 10 ms < τ_D2_ < 250 ms, are diffusion-related and reflect properties of opioid receptors. Hence, a model assuming lateral diffusion of two components and triplet state formation (equation [1] in the main text) was chosen as the simplest model for fitting experimentally derived tACC. When fitting tACCs, all parameters in equation [1] were kept free.

Not surprisingly, average values of characteristic decay times (τ_D1_ and τ_D2_) and their corresponding relative amplitudes (*y* and (1 – *y*)) differed somewhat between different series of experiments (Table S1), subject to cell batch and passage number in culture, but the overall trend within the same series of experiments was always the same, with KOP showing the largest relative contribution of the second component, (1 - *y*), whereas corresponding values were lowest for MOP_N40D_ (Table S1). Also, standard deviation between measurements recorded on the same cell was smaller than the standard deviation of measurements between different cells. See for example the closely overlapping tACC normalized to the same amplitude shown in Fig. S4B, and the values given in Table S1.

Finally, we would like to point out that while FCS is often used to determine the diffusion coefficient (*D*) of molecules of interest, we have opted not to convert the diffusion times τ_D1_ and τ_D2_ into diffusion coefficients as this is not straight forward in complex systems. It is well known that chemical interactions affect the apparent diffusion behavior of molecules ([29](#_ENREF_29)). Thus, transient association of opioid receptors with domains would be reflected in FCS as longer diffusion times because the receptor is temporarily “stalled” when associated with domains. Conversely, if the receptor is excluded from domains, the area that is accessible by free diffusion is smaller than the total area that is determined by FCS calibration measurements. This would be reflected in FCS as shorter diffusion times, but without implying faster mobility.

Dual-color FCCS was used to assess binding between two species labeled with spectrally distinct fluorophores. For dual-color FCCS measurements, the 488 nm line of the Ar-ion laser was used to excite eGFP fluorescence, the HeNe 543 laser was used to excite TAMRA, Tomato or Alexa Fluor® 594, and the HeNe 633 laser was used to excite Alexa Fluor® 647. Fluorescence was separated from incident light using the main dichroic beam splitter HFT 488/543/633. eGFP fluorescence was transmitted to the detector through a band-pass filter BP 505-530; a band-pass filter BP 560-610 was used for TAMRA and Tomato; a long-pass filter LP 650 was used for Alexa Fluor® 594 and for Alexa Fluor® 647. For dual-color FCCS measurements, fluorescence intensity fluctuations were recorded without pre-bleaching, on the same spot at the apical plasma membrane of PC12 cells in a series of 20 consecutive measurements, each measurement lasting 10 s.

First, we point out that tACC for opioid receptors under dual-color settings (Figs 4B and 5A) did not differ qualitatively from the tACC recorded under single-color settings (Figs. 2D and 2E, Fig. 4C), showing the same number of characteristic decay times, similar decay time values and similar relative contributions of the two opioid receptor fractions. The diffusion times recorded under dual-color settings were somewhat longer, since a larger OVE was used in these measurements, and the photon count rate per molecule was lower under dual-color settings, CPSM ~ 2 kHz as compared to CPSM ~ 5 kHz obtained under single-color settings. Fluorescence photon loss arises under dual-color settings for two reasons: because the signal is split between two detectors and because part of eGFP fluorescence was lost by using a narrower band-pass filter for dual-color than for single-color measurements.

There are two main artifacts in dual-color FCCS that can lead to false conclusions about molecular interactions: imperfect overlap of the OVEs for the spectrally distinct fluorophores, which reduces the cross-correlation amplitude and may lead to a false negative result, and spectral cross-talk, which increases the cross-correlation amplitude and may lead to a false positive result ([30](#_ENREF_30)). To assess the contribution of these factors, cells co-expressing eGFP and Tomato were used as a negative control and cells expressing the eGFP-Tomato fusion protein were used as a positive control. Control experiments were performed using continuous excitation of both fluorophores simultaneously and by alternating excitation, *i.e.* by switching from one laser to the other after 120 µs (Fig. S6). As can be seen from the data presented in Fig. S6 A, spectral cross-talk from the green channel into the red channel can be readily recognized by a large difference in the amplitude of the tACC acquired using continuous excitation of both fluorophores simultaneously (Fig. S6 A1, magenta) as compared to the amplitude of the tACC obtained using alternating excitation (Fig. S6 A1, red circles). At the same time, spectral cross-talk from the red to the green channel was negligible, as evident from the overlapping tACCs recorded in the green channel (Fig. S6 A2). Spectral cross-talk could be effectively avoided by alternating excitation (Fig. S6 A3) and the false positive cross-correlation observed by continuous excitation of both fluorophores simultaneously (Fig. S6 A3, brown) was not observed when alternating excitation was used (Fig. S6 A3, orange circles). When fluorophore excitation is adjusted so that spectral cross-talk is minimized and the tACCs recorded by alternating excitation and by continuous excitation of both fluorophores simultaneously are similar (Fig. S6 B1 and B2), the temporal cross-correlation curves (tCCC) acquired by continuous and alternating excitations overlap (Fig. S6 B3). Thus, by using alternating excitation of fluorophores the negative and positive controls could be effectively distinguished by FCCS (Fig. S6 C).

While it is possible to retrieve the complete tACC from FCS measurements with alternating excitation ([31](#_ENREF_31)), less photons are acquired when alternating excitation is used, necessitating even longer signal acquisition. As this is not optimal for measurements on live cells, we assess the spectral cross-talk in individual channels using alternating excitation. When the tACC recorded with continuous or alternating excitation overlap (Fig. 5B:b1 and b2), we regard the spectral cross-talk between channels to be minimal and record the cross-correlation curve using continuous illumination (Fig. 5B: b3) and evaluate the degree of association from the relative cross-correlation amplitude, i.e. the amplitude of the tCCC relative to the amplitude of the tACC recorded in the green channel (Fig. 5C).

**Control FCCS experiments for opioid receptor association with GM1 ganglioside**

Two different FCCS approaches were used to probe opioid receptor association with GM1 ganglioside in live PC12 cells: (1) using the GM1-specific fluorescently labeled cholera toxin B subunit conjugated with the fluorescent marker Alexa Fluor® 594 or Alexa Fluor® 647 (CTxB-AF594 and CTxB-AF647, respectively) and probing its interactions with opioid receptors C-terminally tagged with eGFP (Fig. 5A); and (2) using BODIPY® FL C5-ganglioside GM1 to visualize endogenous GM1 ganglioside-enriched domains in PC12 cells stably expressing MOP_wt_-Tomato (Fig. S7). While both GM1-selective probes could label live PC12 cells, appreciable cross-correlation between these markers and opioid receptors was not observed in neither of the two assays.

FCCS was also used to quantitatively characterize interactions between CTxB-AF647 and BODIPY® FL C5-ganglioside GM1 in solution (Fig. 5B and C), yielding an apparent equilibrium dissociation constant K_d_ = (2 ± 1)×10^-6^ M (determined from the amplitudes of the tACCs and the tCCC (Fig. 5 B: b1-b3) using Eq. [3]). This K_d_ value agrees within an order of magnitude with flow cytometry measurements showing that the apparent equilibrium binding constant for fluorescently labeled pentameric cholera toxin B-subunit (FITC-CTxB) binding to soluble GM1-pentasaccharide is about K_b_ = 4×10^6^ M^-1^ (K_d_ = 2.5×10^-7^ M) ([32](#_ENREF_32)); and with TIRF measurements showing K_d_ = (3.7 ± 0.3)×10^-7^ M for soluble Alexa 594-CTxB interactions with ganglioside GM1-populated distearoylphosphatidylcholine**:** cholesterol supported lipid bilayers ([33](#_ENREF_33)). Difference between the K_d_ value measured by FCCS and the values measured by flow cytometry and TIRF are likely due to the presence of fluorophores on both reactants in the FCCS assay, while only one reactant was labeled in the other assays. Based on the determined K_d_ value, it is possible to conclude that when local concentration of GM1 is above 20 µM, about 90% of CTxB-AF647 is bound. Hence, selective labeling of PC12 cells with CTxB-AF647 may suggest that cells expressing opioid receptors at low levels, to which CTxB-AF647 readily binds present at their surface GM1 ganglioside levels that give rise to such high local concentrations (Fig. 5A, red). The opposite seems to be true for PC12 cells expressing opioid receptors at high levels, to which CTxB-AF647 binding is limited (Fig. 5A, green).

Taken together, these data suggest that opioid receptors are largely excluded from GM1 ganglioside-enriched domains. However, we also need to underline that CTxB interactions with GM1 ganglioside are notoriously complex and the results of fluorescence labeling assays should be carefully interpreted due to the intricacy of these interactions. For example, it has been recently shown that headgroup accessibility and partitioning of acyl-chain labeled bodipy-GM1 probes may differ from those of native GM1 ([34](#_ENREF_34)), CTxB recognition of GM1 may be significantly reduced by GM1 clustering ([35](#_ENREF_35)) and CTxB can also bind to other glycolipid receptors ([36](#_ENREF_36), [37](#_ENREF_37)). In all of these cases, the GM1 ganglioside-enriched domains visibility by the CTxB reaction would be reduced.

**PALM image analysis protocol**

In PALM images, a single paGFP molecule generally appears in several non-consecutive frames ([38](#_ENREF_38)). To improve data analysis accuracy background was reduced by removing single peaks (approximately 5-7% of total peaks in the image). Peaks were localized using Peak Selector software (Research Systems, Inc.) with a cylindrically symmetric Gaussian point spread function ([39](#_ENREF_39)). For each image, we obtained a distribution of localization precisions (σ) and determined a value that encompasses 98% of the total peaks, σ_MAX_. To remove single peaks, the following steps were performed. We created a first dataset containing the coordinates of all identified peaks. Then a second dataset was created containing the coordinates of grouped peaks. The peaks were grouped using the group radius of 3σ_MAX_ and a maximum dark time of 25.5 seconds (this value is much longer than typical paGFP maximum dark time). Finally, the two datasets were compared in Microsoft Access. Peaks with the same coordinates in the two datasets were discarded (single peaks).

Datasets without single peaks were finally grouped using group radius of 3σ_MAX_ and maximum dark time of 0 s to group only consecutive frames of single paGFP molecules. The resulting coordinates were used for PC-PALM analysis using code custom written in MATLAB (The Mathworks, Inc., Natick, MA) as reported before ([38](#_ENREF_38), [40](#_ENREF_40)). The average number of appearances of individual paGFP molecules (due to blinking) was estimated to be equal to 5-6. These numbers were calculated using image analysis of 1) very sparse paGFP covalently attached on the surface (random distribution, auto-correlation curve = 1) and 2) sparsely expressed paGFP tagged constructs in cells. Same imaging parameters were used in all cases.

To validate this protocol, we first detected organization of two model proteins: glycosylphosphatidylinositol-anchored protein (GPI), and trimeric protein vesicular stomatitis viral glycoprotein (VSVG). We used established VSVG and GPI constructs tagged with photoactivatable green fluorescent protein (paGFP) ([38](#_ENREF_38)) overexpressed in COS-7 and MDA-MB-468 cells, respectively. To calculate auto-correlation functions, random square regions (10-24 µm^2^) from individual cells were analyzed as described before ([38](#_ENREF_38)). All autocorrelation curves for GPI and VSVG were fitted to a single exponential function with a fast decay (Fig. S8, R^2^ ≥ 0.95), which was consistent with the organization of proteins within tight domains. For paGFP-GPI, 1-4 proteins were detected in each domain (N=16); for VSVG-paGFP 3 proteins, on average, were detected in each domain (N = 12). These protein distributions were consistent with previous results ([11](#_ENREF_11), [38](#_ENREF_38)) and other published data ([41-43](#_ENREF_41)). We compared experimental data to correlation functions obtained from ensemble-averaged Monte Carlo simulations ([44](#_ENREF_44)) similar to the approach applied for opioid receptors. Briefly, from simulated images of paGFP-GPI and VSVG-paGFP, we generated position estimates, calculated correlation functions (using the same software and algorithms used to analyze experimental data), and compared them with experimental results. We obtained excellent agreement between experiments and simulations (Fig. S8B and S8D).

**Monte Carlo Simulations**

Numerical simulations of images were performed. The following key variables were used:

1. The probabilities of different numbers of proteins existing in a domain (1, 2, 3, etc.) and, for complex organizations, the distribution of these proteins among different oligomer types (*i.e.* fraction of proteins in monomers, dimers, and higher order oligomers). The number of domains simulated equaled the number of proteins in the field of view divided by the average number of proteins per domain.
2. The protein radius. Proteins were modeled as hard disks interacting via excluded-area forces; i.e. proteins could not overlap in our simulation, but no longer-range forces between proteins were assumed. This parameter was typically 13 nm.
3. Protein separation in oligomers (i.e. center-to-center spacing, Figs S8 and S9A-C, left panel). We did not assume *a priori* that this equaled the protein radius used for excluded-area interactions with other domains so that we could approximate domains with irregular shapes using partially-overlapping disks if necessary. In other words, we assumed that different oligomers cannot overlap, but within an oligomer, we allowed overlaps to approximate elliptical geometries. For the VSVG trimer, we used experimental estimates and thus set the protein separation equal to 26 nm.
4. The average number of photons *N* obtained from a protein during a given frame and the average localization precision *σ*. These variables were connected to the width *w* of the PSF via the approximation . The actual number of photons obtained from a protein in a given frame was drawn from a Poisson distribution with mean *N*. The average number of photons per frame was typically assumed to be between 160 and 170 and was based on the average number of photons obtained from super-resolution data.
5. The average number of appearances *n* per fluorophore, i.e. the average number of frames in which a fluorophore appears. This variable was set to the same value obtained from experimental data. The actual number of frames *k* in which a given fluorophore produced an image was drawn from a geometric distribution of the form $p {(1-p)}^{k-1}$, where $p=\frac{1}{n}$ is the probability of the fluorescent protein irreversibly turning off at the end of the frame.
6. The size of the field of view. Larger fields of view held larger numbers of fluorophores, reducing noise (due to finite size effects) in the correlation functions computed on the simulation output.
7. The density of proteins per square micron. The density of proteins was set equal to the experimentally observed average number of fluorophore appearances per square micron, divided by the experimentally measured average number of appearances per protein. We used the density of proteins per square micron and the average number of proteins per domain to determine how many domains to model.
8. The minimum and maximum domain radii. No significant domain size heterogeneity was observed in experiments, so the minimum and maximum domain radii typically varied by no more than 10% in simulations, and the distribution of domain radii was assumed (for simplicity) to be uniform. Uniform domain radii were generated by using the same numbers for the minimum and maximum radii.
9. The range *r_int_* and strength *V_0_* of attractive interactions between domains, to model the fact that correlation functions did not decay to unity at long distances. We assumed that domain-domain interactions were weak and thus dominated by nearest neighbors. We used a potential of the form $V\left( r \right)= V_{0} (1- \frac{1}{1+{( \frac{r}{r_{\mathrm{int}}})}^{2}})$ where *r* is the edge-to-edge separation between a domain and its nearest neighbor (i.e. if the center-to-center spacing of two domains was 200 nm, and the domain radii were both 90 nm, then *r* would be 20 nm). In most simulations we either used *V_0_* = 0 (no interactions) or *V_0_* = 2 (approximately 14% probability of accepting domain positions outside the interaction range). We do not interpret this interaction potential as a literal potential energy; we have no reason to assume that the distribution of domain separations reflects a thermal equilibrium. Rather, we used this potential only as a convenient way of generating a distribution of domain sizes (as discussed below).

Simulations proceeded in four stages: Domain generation, dimer/oligomer generation (when applicable), appearance generation, and image generation. Domains were generated one at a time. Candidate domain positions (center coordinates) were drawn from a uniform distribution over the simulated field of view.

Candidate domain radii were generated from a uniform distribution (typically with a very narrow range, no more than 10% of the mean). If the candidate position and radius did not yield an overlap with an existing domain, the potential was computed based on the distance to the nearest neighbor; and a random number between 0 and 1 was generated. In accordance with the Metropolis algorithm ([45](#_ENREF_45)), the candidate domain position and radius were accepted if the random number was smaller than . This process was repeated until the required number of domains was generated.

Once all domains had been generated, they were populated with proteins. The number of proteins in the domain was generated from a user-defined discrete probability distribution. When applicable, we then generated candidate dimers/oligomers one at a time from user-defined discrete probability distribution. Candidate dimers/oligomers were accepted if none of their proteins overlapped proteins from other dimers/oligomers in that domain (assuming excluded-area interactions between circular proteins with user-defined radii). This process was continued until the domain was occupied with the specified number of proteins.

Subsequently, localizations (protein coordinate estimates) were generated. For each protein, a number of appearances was first generated from a geometric distribution with a user-defined mean. Next, for each appearance, a photon count was generated from a Poisson distribution with a user-defined mean. From this mean and the PSF width a localization precision was computed. The position of each appearance was generated from a 2D Gaussian distribution centered at the protein’s true position, with a standard deviation equal to the localization precision.

From these lists of localizations we generated images in a format similar to the experimental images, so that they could be fed into MATLAB code used to compute the auto-correlation functions of the experimental images. Finally, we computed the auto-correlation function of simulated images.

For KOP, MOP_wt_, and MOP_N40D_, we systematically varied the fraction of monomers/dimers/oligomers in domains and calculated the normalized inner product between experiment and simulations. Best fit (monomers for KOP and MOP_N40D_, 67% dimers/33% monomers for MOP_wt_) is presented in Fig. 3; however other options could not be completely excluded. We could exclude the formation of higher order oligomers (i.e. tetramers) within domains based on poor agreement with the experimental data.

**Table S1:** Comparison between tACC for KOP, MOP_wt_ and MOP_N40D_ across different experiments.

| **Fitting results on individual cells** | | | | | | | |  | | | | |  |  |  |  |  |  |  |  |
| --- | --- | --- | --- | --- | --- | --- | --- | --- | --- | --- | --- | --- | --- | --- | --- | --- | --- | --- | --- | --- |
| **Figure** | **Receptor** | **1-*y*** | | | **τ_D1_ / µs** | | **τ_D2_ / ms** |  | | | | | |  |  |  |  |  |  |  |
| **Fig. 2D** | MOP_wt_-eGFP | 0.37 ± 0.04 | | | 280 ± 30 | | 50 ± 8 |  |  |  |  |  |  |  |  |  |  |  |  |  |
| **Fig. 2E*** | KOP-eGFP | 0.40 ± 0.10 | | | 300 ± 30 | | 40 ± 20 |  |  |  |  |  |  |  |  |  |  |  |  |  |
|  | MOP_wt_-eGFP | 0.27 ± 0.08 | | | 400 ± 50 | | 40 ± 30 |  |  |  |  |  |  |  |  |  |  |  |  |  |
|  | MOP_N40D_-eGFP | 0.19 ± 0.07 | | | 400 ± 50 | | 12 ± 10 |  |  |  |  |  |  |  |  |  |  |  |  |  |
| **Fig. 4B** | MOP_wt_-eGFP | 0.37 ± 0.04 | | | 300 ± 30 | | 90 ± 40 |  |  |  |  |  |  |  |  |  |  |  |  |  |
| **Fig. S4B** | MOP_wt_-eGFP | 0.36 ± 0.04 | | | 290 ± 40 | | 40 ± 10 |  |  |  |  |  |  |  |  |  |  |  |  |  |
| **Fig. S5B** | KOP-eGFP | 0.43 ± 0.03 | | | 360 ± 40 | | 90 ± 40 |  |  |  |  |  |  |  |  |  |  |  |  |  |
| **Comparison between different receptors** | | | | | | | | | | | | | | | | | | | | |
| **Figure** | **Receptor** | **(1-*y*)*/ y*** | | | **n_Cell_** | | **n_tACC_** | **P**** | | | | | | | **1-*y*** | | **τ_D1_ / µs** | | **τ_D2_ / ms** | |
| **Fig. 2F** | KOP-eGFP | 0.42 ± 0.06 | | | 10 | | 100 |  | <0.05 | |  |  | | | 0.30 ± 0.04 | | 410 ± 80 | | 90 ± 50 | |
|  | MOP_wt_-eGFP | 0.30 ± 0.15 | | | 25 | | 250 |  |  |  | <0.05 | < 0.02 | | | 0.23 ± 0.09 | | 310 ± 60 | | 80 ± 40 | |
|  | MOP_N40D_-eGFP | 0.16 ± 0.07 | | | 9 | | 90 |  | | |  |  | | | 0.14 ± 0.06 | | 230 ± 60 | | 40 ± 20 | |
| **Fig. S11C** | GFP-KOP | 0.43 ± 0.04 | | | 14 | | 140 | < 0.05 | | | | | | | 0.30 ± 0.09 | | 350 ± 80 | | 80 ± 80 | |
|  | GFP-MOP_wt_ | 0.29 ± 0.03 | | | 14 | | 140 |  |  |  |  |  |  |  | 0.23 ± 0.08 | | 280 ± 60 | | 60 ± 40 | |
| **Cholesterol sequestration using MβCD***** | | | | | | | | | | | | | | | | | | | | |
| **Figure** | **Receptor** | | **Control** | **MβCD** | | **Control** | | **MβCD** | | **Control** | | **MβCD** | | | **n_Cell_** | **n_tACC_** | **P****** | | | |
|  |  |  | **1-*y*** | **1-*y*** | | **τ_D1_ / µs** | | **τ_D2_ / ms** | | **τ_D1_ / µs** | | **τ_D2_ / ms** | | |  |  |  |  |  |  |
| **Fig. 4C** | KOP-eGFP | | 0.5 ± 0.1 | 0.4 ± 0.1 | | 510 ± 120 | | 200 ± 80 | | 410 ± 40 | | 100 ± 60 | | | 10 | 100 | <0.05 |  | |  |
|  | MOP_wt_-eGFP | | 0.4 ± 0.1 | 0.34 ± 0.05 | | 310 ± 60 | | 80 ± 40 | | 260 ± 22 | | 60 ± 20 | | | 15 | 150 |  | <0.05 | |  |
|  | MOP_N40D_-eGFP | | 0.32 ± 0.08 | 0.31 ± 0.07 | | 280 ± 60 | | 70 ± 40 | | 250 ± 60 | | 60 ± 30 | | | 10 | 100 |  |  | | > 0.5 |

*Average tACCs, n = 8-10 cells.
**P values refer to differences between the slopes of the linear regression lines for different receptors, slope = (1-y)/y. The difference is considered to be statistically significant when P < 0.05. P values were calculated using Excel.
***The cells were cultured in serum-free medium.
****P values refer to differences between the relative contributions of the second component, characterized with longer diffusion time, 1-*y*. The difference is considered to be statistically significant when P < 0.05. P values were calculated using Excel.

**Table S2**: F-test and S-values between correlation curves reported in Figs 3B, 3D, 3F, 4D, S9D, and S9E. Matlab built-in function ‘vartest2’ was used to calculate the F-test statistic and this value was subsequently compared to the F-critical value based on the number of data points (F-critical= 1.2172; # of observations 282). The null hypothesis is accepted if the variances of two populations are equal within 5%. Standard errors of the estimate (S-values) were calculated using Matlab ([46](#_ENREF_46)). PPD=protein per domain.

|  | **F-Test** | **Result 95%** | **S-Value** |
| --- | --- | --- | --- |
| **Experimental Data vs. Simulation (Figs 3B, 3D, 3F, top 3 lines; Fig. 4D, bottom two lines)** | | | |
| KOP exp. vs KOP simulation | 1.1689 | ACCEPT | 0.19 |
| MOP_wt_ exp. vs MOP_wt_ simulation | 1.021 | ACCEPT | 0.13 |
| MOP_N40D_ exp. vs MOP_N40D_ simulation | 1.2056 | ACCEPT | 0.34 |
| KOP MβCD clustered exp. vs KOP MβCD clustered simulation | 1.0041 | ACCEPT | 0.08 |
| MOP_wt_ MβCD clustered exp. vs MOP_wt_ MβCD clustered simulation | 1.0058 | ACCEPT | 0.05 |
| **Experimental Data vs. Experimental Data (Fig. 3)** | | | |
| KOP exp. vs MOP_wt_ exp. | 1.7022 | REJECT | 0.43 |
| KOP exp. vs MOP_N40D_ exp. | 2.1436 | REJECT | 0.75 |
| MOP_wt_ exp. vs MOP_N40D_ exp. | 1.2594 | REJECT | 0.48 |
| **Varying proteins per domain vs. MOP experimental (Fig. S9E, left)** | | | |
| 9-10 PPD, 86 nm radius simulation vs MOP_wt_ exp. | 1.3717 | REJECT | 0.32 |
| 8-9 PPD, 86 nm radius simulation vs MOP_wt_ exp | 1.0114 | ACCEPT | 0.12 |
| 7-8 PPD, 86 nm radius simulation vs MOP_wt_ exp | 1.3223 | REJECT | 0.27 |
| **Varying Radius vs. MOP experimental (Fig. S9E, right)** | | | |
| 8-9 PPD, 79 nm radius (MOP_N40D_ radius) simulation vs MOP_wt_ exp | 1.3772 | REJECT | 0.31 |
| 8-9 PPD, 101 nm radius (KOP radius) simulation vs MOP_wt_ exp | 1.7993 | REJECT | 0.47 |
| **Bimodal distribution vs. MOP experimental (Fig. S9D)** | | | |
| 50% dimers, 50% 6 MOPs in 86 nm domain simulation vs MOP_wt_ exp | 2.7827 | REJECT | 0.76 |
| 75% dimers, 25% 6 MOPs in 86 nm domain simulation vs MOP_wt_ exp | 2.991 | REJECT | 0.82 |
| 50% dimers, 50% 7 MOPs in 86 nm domain simulation vs MOP_wt_ exp | 2.0359 | REJECT | 0.57 |
| 78% dimers, 22% 7 MOPs in 86 nm domain simulation vs MOP_wt_ exp | 2.3945 | REJECT | 0.69 |
| 50% trimers, 50% 6 MOPs in 86 nm domain simulation vs MOP_wt_ exp | 2.0246 | REJECT | 0.60 |
| 67% trimers, 33% 6 MOPs in 86 nm domain simulation vs MOP_wt_ exp | 1.7544 | REJECT | 0.57 |

**
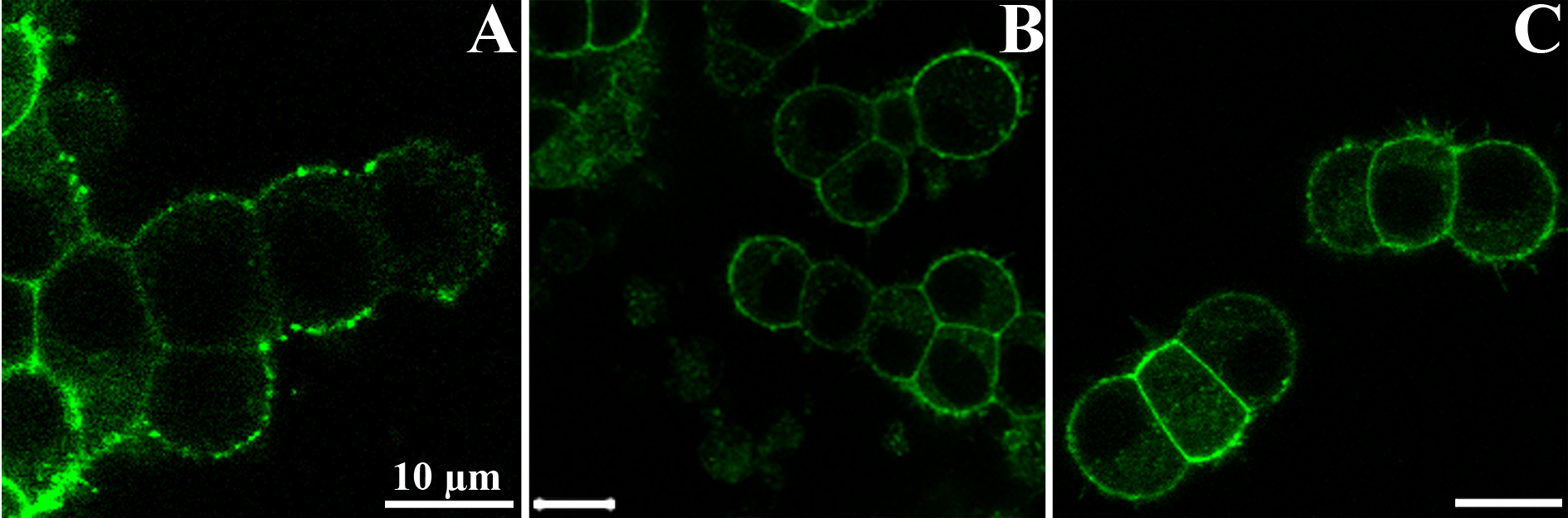
**

**Figure S1. CLSM images of live PC12 cells stably transformed to express different opioid receptors.** **A.** KOP-eGFP. **B.** MOP_wt_-eGFP. **C.** MOP_N40D_-eGFP. Scale bar 10 µm.

**
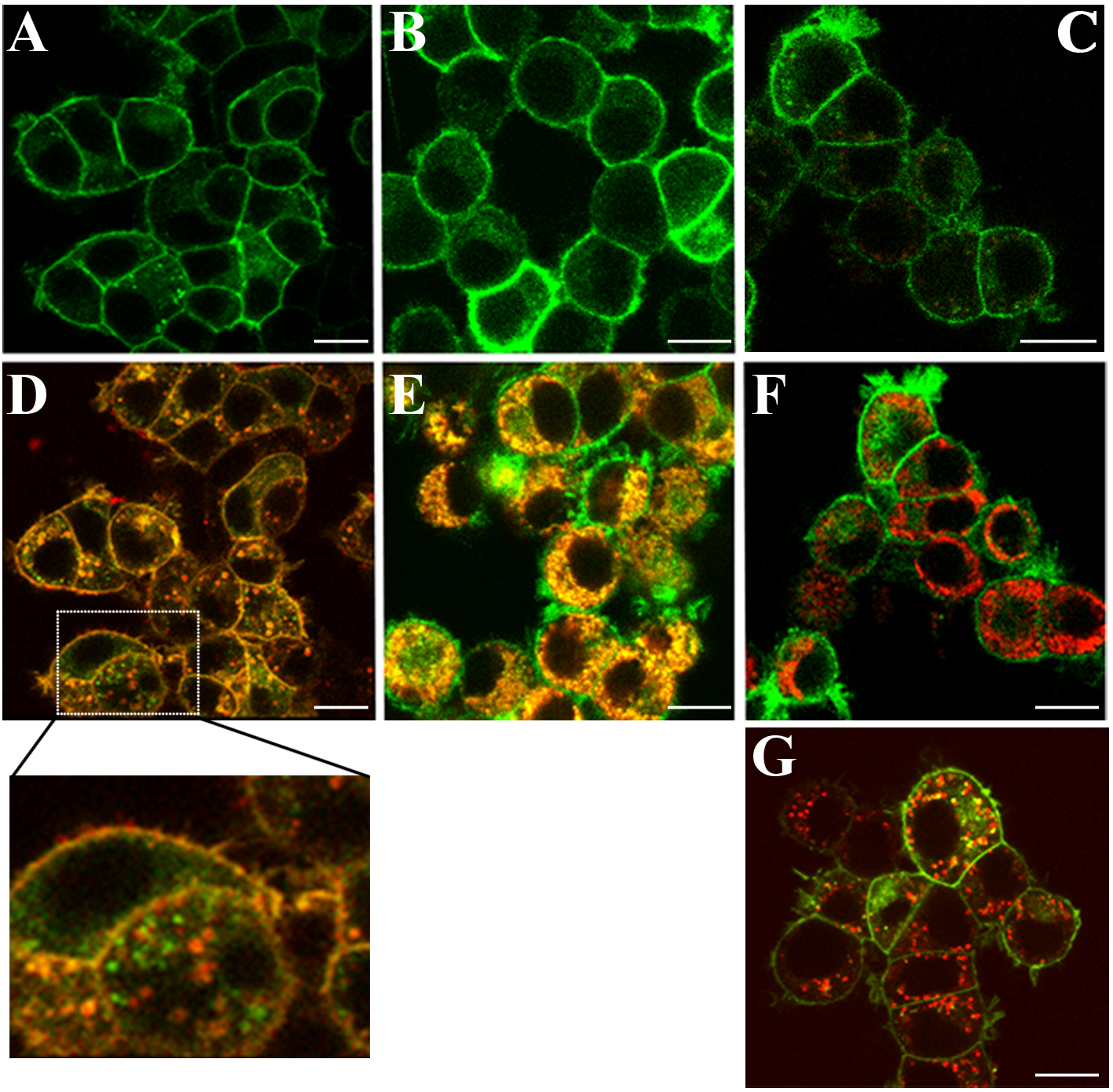
**

**Figure S2. CLSM images of live PC12 cells expressing different opioid receptors before and after stimulation with corresponding specific ligands.** **A-C.** eGFP-tagged opioid receptor (green) distribution in live PC12 cells before stimulation (t = 0 min): KOP-eGFP (A); MOP_wt_-eGFP (B); MOP_N40D_-eGFP (C). **D-F.** CLSM images of the same cells (shown in A-C) after stimulation with corresponding specific peptide ligands fluorescently labeled with TAMRA. **D.** KOP-eGFP (green) internalization observed after 15 min of stimulation with 150 nM Dyn A-TAMRA (red). The magnified detail (dotted line box) shows inward moving trafficking vesicles (yellow) containing ligand-receptor complexes and/or both the ligand and the receptor, which can be identified through co-localization between the green and the red signal; outward moving trafficking vesicles (green) transporting newly synthesized KOP-eGFP receptors to the plasma membrane; and lysosomes (red), where eGFP is degraded and TAMRA (not biodegradable) accumulates. **E.** Massive internalization of MOP_wt_-eGFP (green) observed after 5 min stimulation with 250 nM β-Endorphin-TAMRA (red). **F.** Live PC12 cells expressing MOP_N40D_-eGFP (green) imaged 9 min after treatment with 200 nM β-Endorphin-TAMRA (red) show that the ligand is rapidly internalized (red), limited internalization of the receptor was observed. **G.** Live PC12 cells expressing MOP_N40D_-eGFP (green) imaged 10 min after stimulation with 100 nM hepta enkephalin-TAMRA (red). CLSM imaging shows that the ligand (red) and to some extent ligand-receptors (yellow) are internalized. Scale bar 10 µm.

**
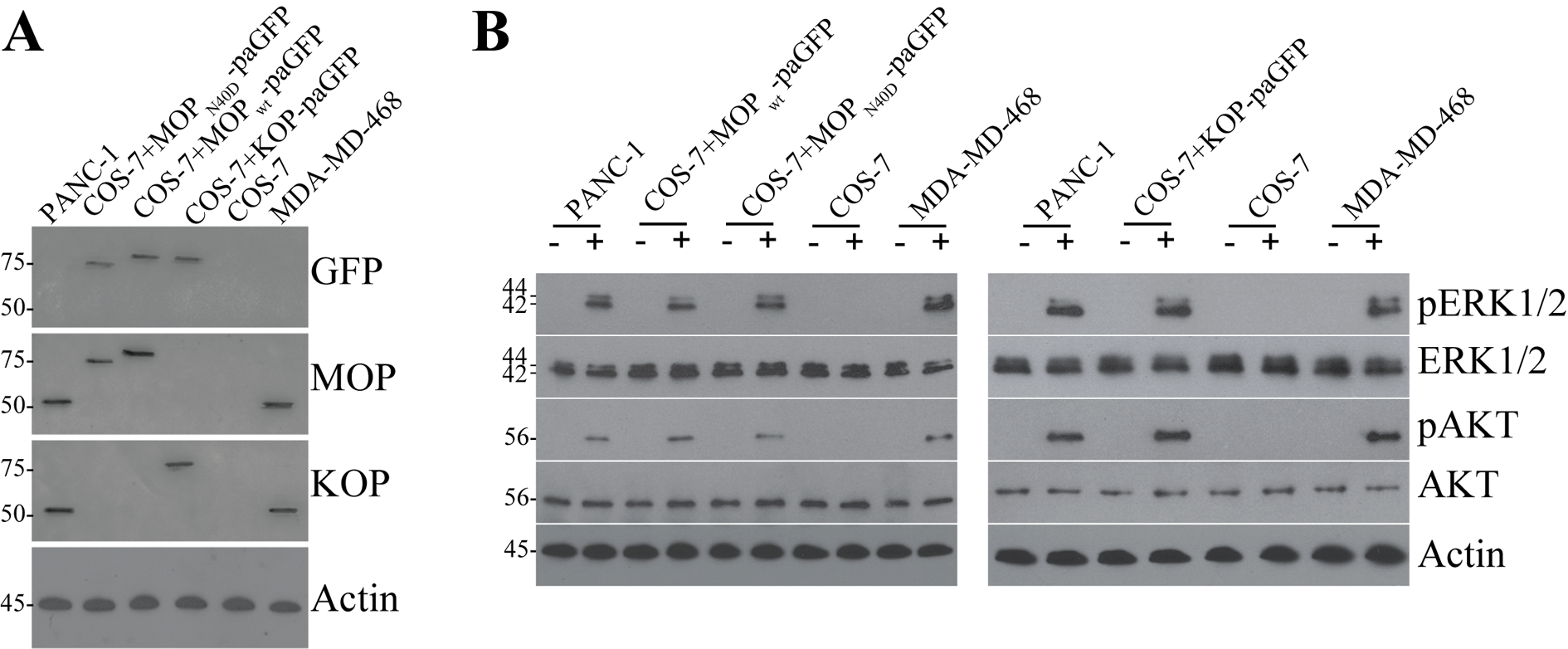
**

**Figure S3. Expression levels and activities of opioid receptor constructs in COS-7 cells.** **A.** Endogenous MOP and KOP in MDA-MB-468 and PANC-1 cells have expression levels comparable to that of MOP_wt_-paGFP and KOP-paGFP transiently expressed in COS-7 cells. MOP_wt_-paGFP was expressed at higher levels than MOP_N40D_-paGFP. Change in MW between MOP_wt_ and MOP_N40D_ is likely due to loss of glycosylation site in mutant variant. **B.** Dermorphin activation of MOP_wt_-paGFP and MOP_N40D_-paGFP expressed in COS-7 cells (left) and dynorphin activation of KOP-paGFP expressed in COS-7 cells (right) leads to phosphorylation of AKT and ERK1/2; similar effect is observed with endogenous receptors.

**
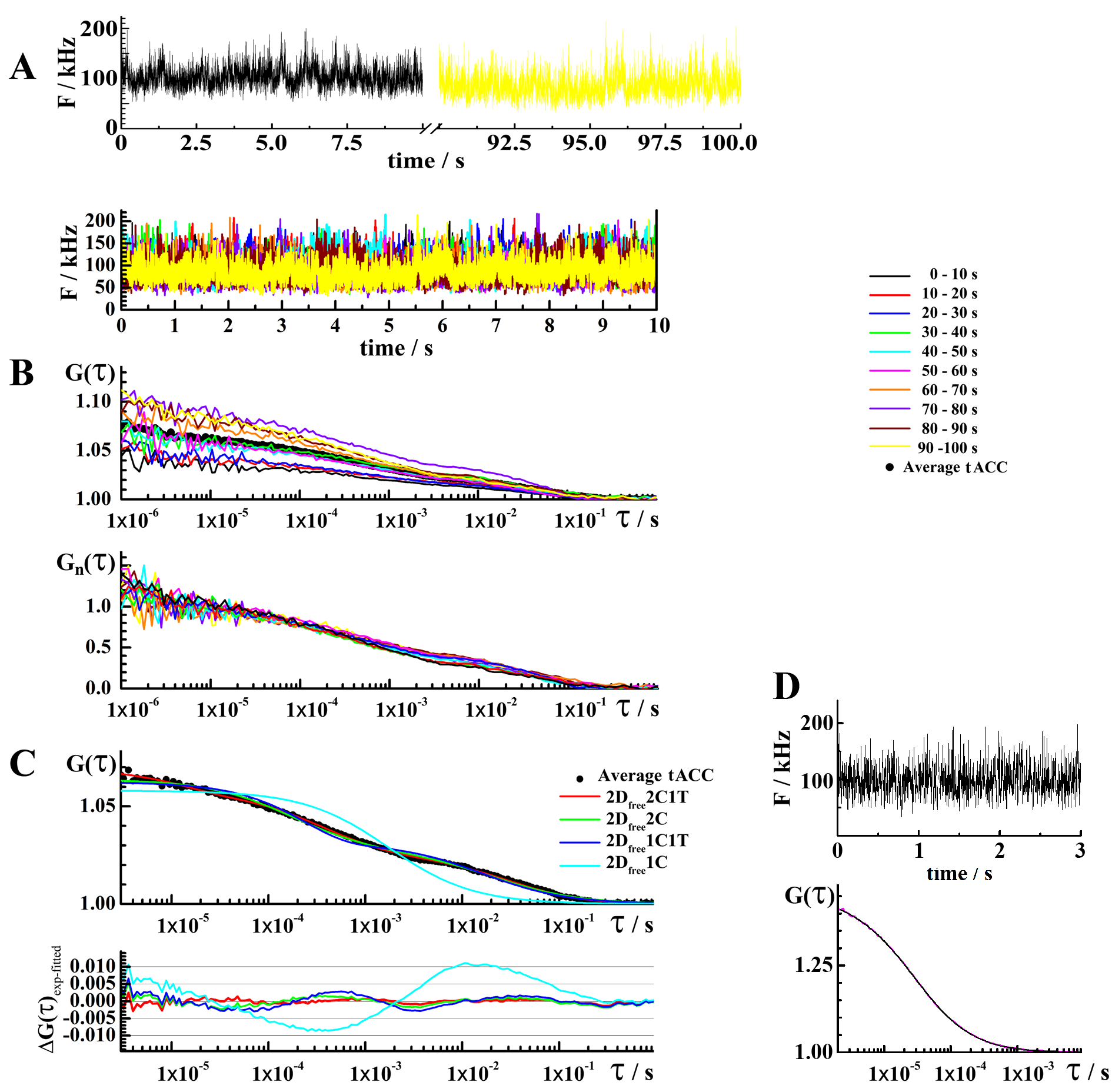
**

**Figure S4. FCS measurements and fitting analysis of experimentally derived temporal autocorrelation curves (tACC) for MOP_wt_-eGFP. A.** Top: First (black) and last (yellow) fluorescence intensity count trace, F(t), acquired at the same spot on the apical plasma membrane in a series of 10 consecutive measurements, each measurement lasting 10 s. Bottom: Overlaid 10 fluorescence intensity count traces from the series above. **B.** Top: Corresponding tACC, G(τ), calculated from the individual 10 s runs (lines) and the average tACC for the whole series, *i.e.* for all 10 measurements (black dots). Bottom: tACC curves presented above normalized to the same amplitude, G_n_(τ) = 1 at τ = 10 µs. Graphs of the same run in A and B are displayed with the same color. Statistical considerations are provided in Table S1. **C.** Top: Fits of the experimentally derived average tACC assuming different underlying processes. Cyan - single species freely diffusing in the two-dimensional plasma membrane and no triplet state (2D_free_1C); Blue - single species freely diffusing in the plasma membrane and triplet state (2D_free_1C1T); Green - two species freely diffusing in the plasma membrane and no triplet state (2D_free_2C); Red - two species freely diffusing in the plasma membrane and triplet state (2D_free_2C1T). Bottom: Corresponding fit residuals. Graphs of the same run are displayed with the same color. Smallest residuals, *i.e.* best fitting is obtained using the model for two species freely diffusing in the plasma membrane and a triplet state (2D_free_2C1T). **D.** Top: Fluorescence intensity fluctuations recorded in a 25 nM aqueous solution of Rh6G (top). Bottom: Corresponding tACC (magenta) fitted using a model for a single freely diffusing species in three dimensions and triplet state (3D_free_1C1T) (black). Diffusion time of Rh6G was τ_D_ = (27 ± 3) µs. The structure parameter, as defined by the Zeiss software for tACC analysis, was (6 ± 1).


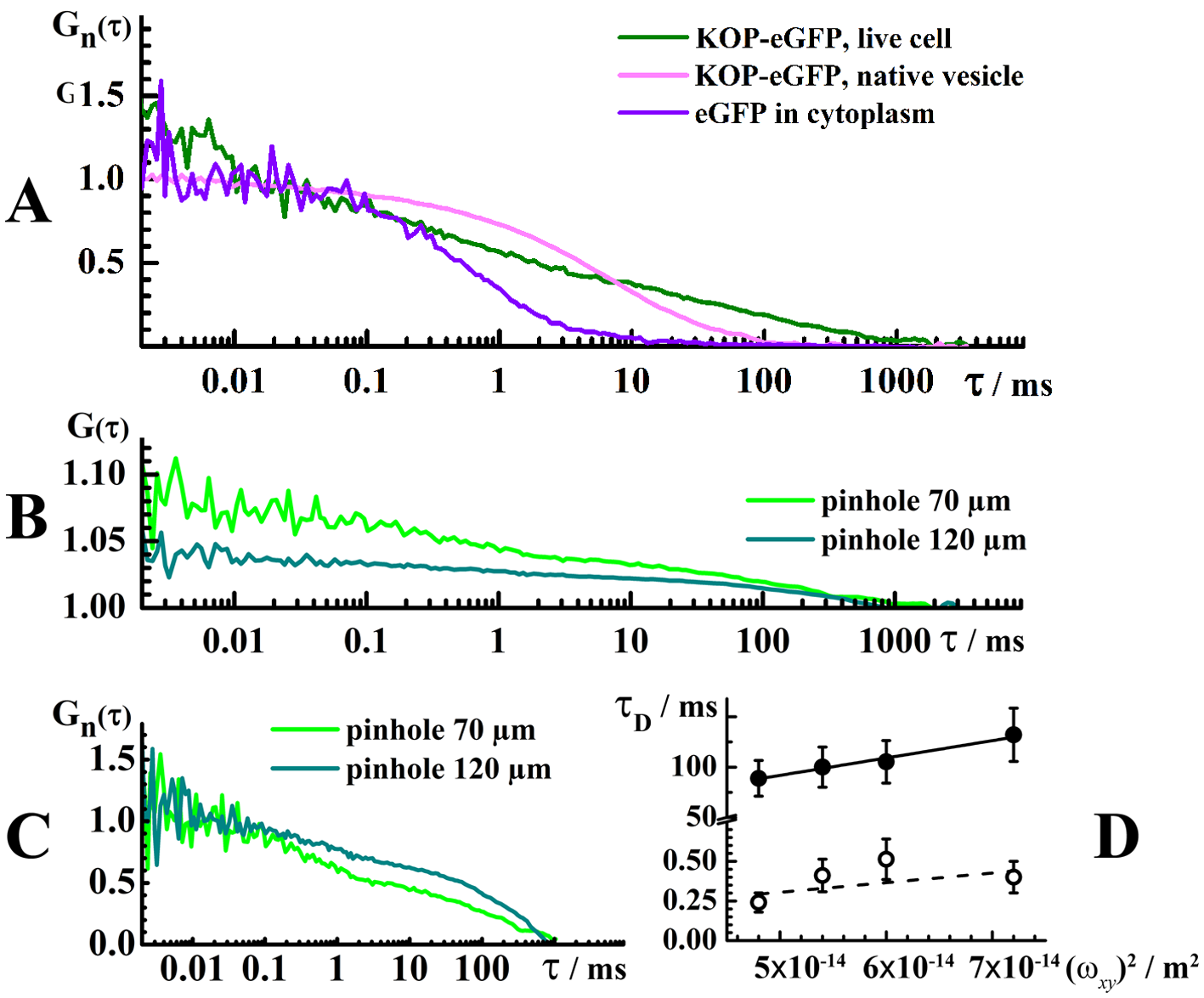


**Figure S5. Experimental validation of FCS data interpretation by fitting analysis. A.** tACC recorded at the apical plasma membrane of a live PC12 cell stably transformed to express KOP-eGFP (dark green), showing two principle diffusion-related characteristic decay times, τ_D1_ = (360 ± 40) µs and τ_D2_ = (90 ± 40) ms. tACC recorded at a giant native vesicle spontaneously formed by cell swelling in the culture of PC12 cells stably expressing KOP-eGFP, showing a single diffusion-related characteristic decay time τ_D,vesicle_ = (7 ± 1) ms that falls between τ_D1_ and τ_D2_ (light magenta). tACC recorded in the cytoplasm of live cells expressing eGFP, showing a single diffusion-related characteristic decay time τ_D,eGFP_ = (450 ± 50) µs (violet). **B.** tACC recorded at the apical plasma membrane of a live PC12 cell stably transformed to express KOP-eGFP recorded using OVEs of different size, as defined by the pinhole: 70 µm (green) and 120 µm (dark cyan). **C.** tACC shown in B, normalized to the same amplitude, G_n_(τ) = 1 at τ = 1×10^-5^ s. **D.** Characteristic decay times τ_D2_ (dots) and τ_D1_ (circles) scale linearly with the radial waist area of the OVE, indicating that the underlying processes are diffusion dependent.

**
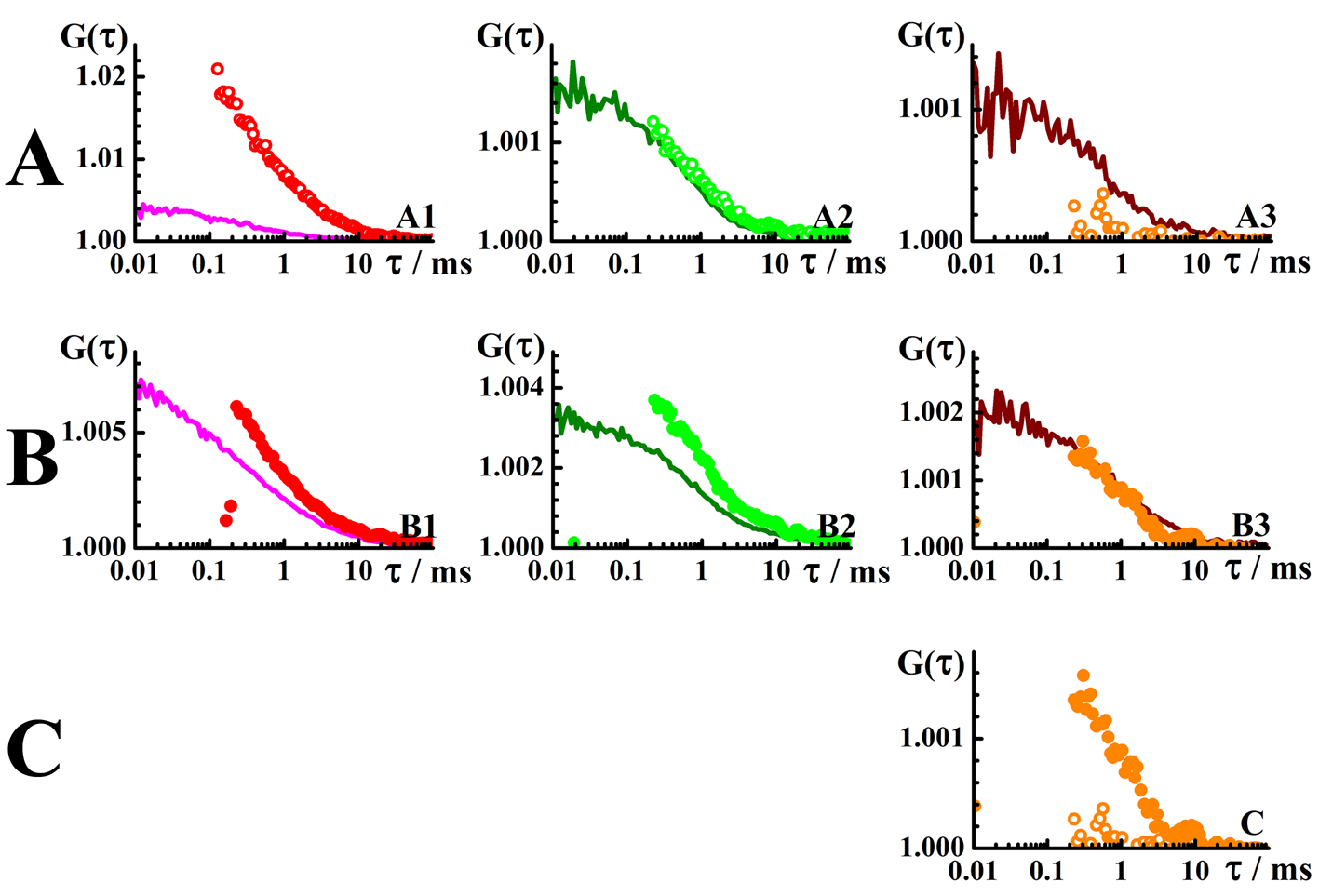
**

**Figure S6. Circumventing spectral cross-talk in dual-color FCCS by alternating excitation.** **A.** *Negative control:* *tACC recorded in the cytoplasm of cells expressing eGFP and Tomato.* **A1:** tACC recorded in the red channel using continuous excitation in both channels simultaneously (magenta) *versus* alternating excitation (red circles). **A2:** tACC recorded in the green channel using continuous excitation in both channels simultaneously (olive) *versus* alternating excitation (green circles). **A3:** tCCC recorded using continuous excitation in both channels simultaneously (brown) *versus* alternating excitation (orange circles). **B.** *Positive control:**tACC recorded in the cytoplasm of cells expressing eGFP-Tomato fusion protein.* **B1:** tACC recorded in the red channel using continuous excitation in both channels simultaneously (magenta) *versus* alternating excitation (red circles). **B2:** tACC recorded in the green channel using continuous excitation in both channels simultaneously (olive) *versus* alternating excitation (green circles). **B3:** tCCC recorded using continuous excitation in both channels simultaneously (brown) *versus* alternating excitation (orange dots). **C.** tCCC recorded using alternating excitation in cells expressing eGFP and Tomato (orange circles) and in cells expressing the eGFP-Tomato fusion protein (orange dots). The switching time was 120 µs.


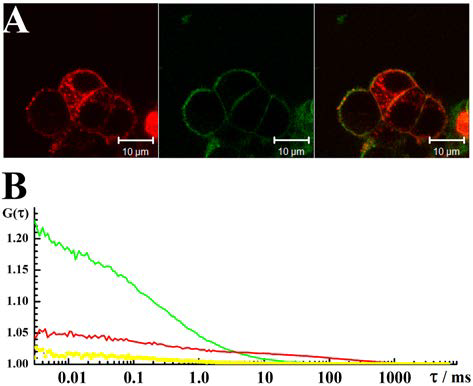


**Figure S7. Dual-color FCCS analysis of MOPwt-Tomato interactions with BODIPY® FL C5-ganglioside GM1.** **A.** CLSM image of live PC12 cells stably transformed to express MOPwt-Tomato (red) treated with BODIPY® FL C5-ganglioside GM1 (green). **B.** tACC of MOPwt-Tomato (red) and BODIPY® FL C5-ganglioside GM1 (green), and the corresponding tCCC (yellow). Fluorescence intensity fluctuations were recorded at the apical plasma membrane using continuous excitation.


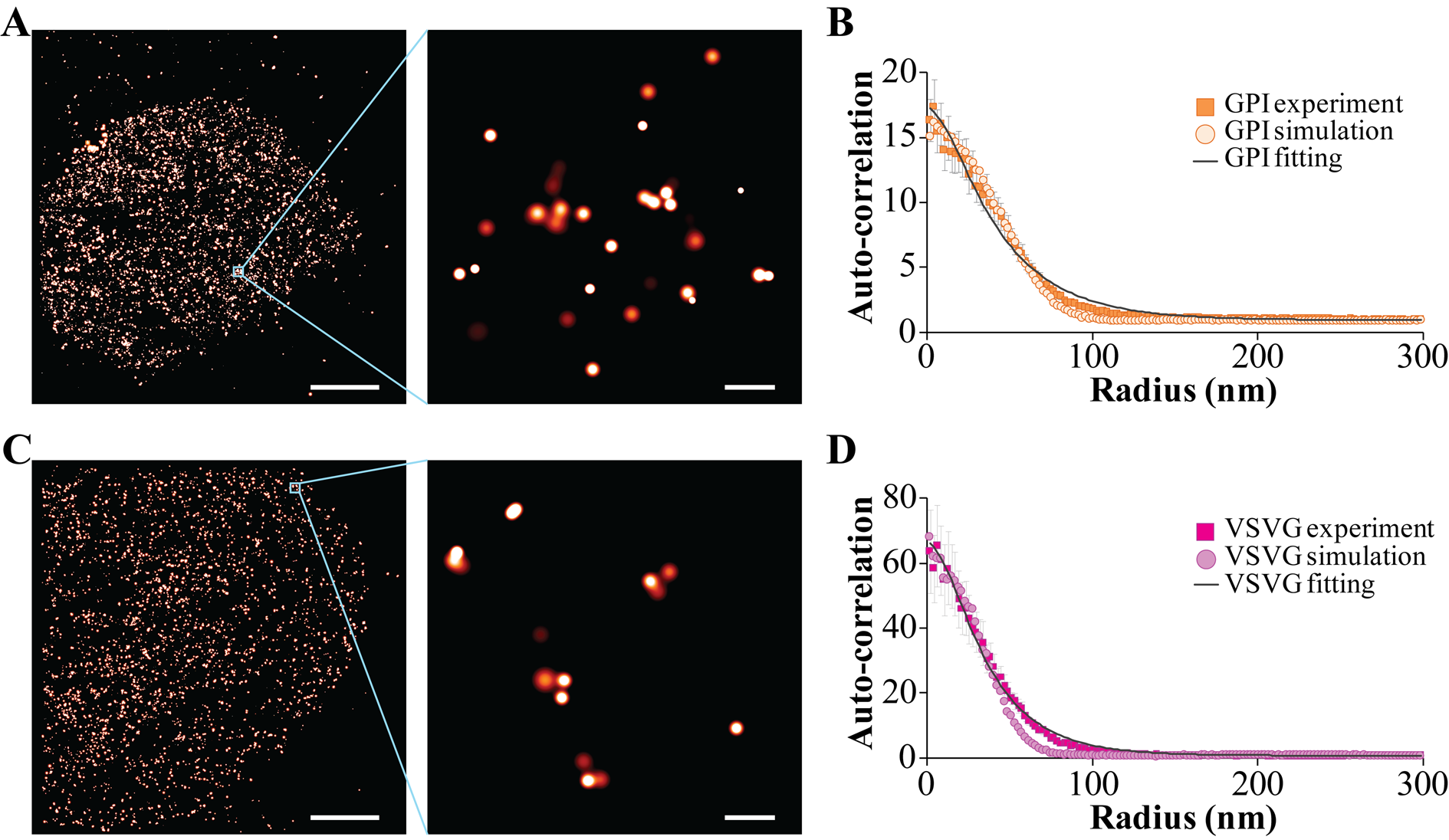


**Figure S8. paGFP-GPI and VSVG-paGFP molecular distribution. A.** A super-resolution microscopy image of the GPI distribution in MDA-MB-468 cells. The whole area is shown on the left (scale bar, 5 μm), and a magnified area is shown on the right (scale bar, 100 nm). **B.** The average GPI auto-correlation function based on experimental data (orange squares) and simulation (light orange circles). Simulation parameters were based on results of PC-PALM analysis: 17 ± 1 molecules per µm^2^, average domain radius of 37 nm, 1-4 proteins in each domain (8 cells, N = 16). **C.** The super-resolution microscopy image of the VSVG distribution in COS-7 cells. The whole area is shown on the left (scale bar, 5 μm), and a magnified area is shown on the right (scale bar, 100 nm). **D.** The average VSVG auto-correlation function based on experimental data (magenta squares) and simulation (light magenta circles). Simulation parameters were based on results of PC-PALM analysis: 7 ± 1 molecules per µm^2^, an average domain radius of 32 nm, 3 proteins in each domain (6 cells, N = 12). In presented images, we grouped peaks within the group radius of 3σ_MAX_ and maximum dark time of 5 s. Standard error bars are shown. A single exponential fit for average protein auto-correlation function is shown in black and it well describes the autocorrelation functions of both GPI and VSVG. Super-resolution images were generated by analyzing datasets in PeakSelector ([39](#_ENREF_39)). As demonstrated previously ([11](#_ENREF_11)), protein organization can be inferred either from averaged autocorrelation curves, or from individual correlation curves (results are subsequently averaged). For paGFP-GPI, we obtain 3.1 proteins per cluster and 37 nm cluster radius with the first approach and 2.9 ± 0.2 proteins per cluster and 37 ± 2 nm cluster radius with the second approach. For VSVG-paGFP, we obtain 3.6 proteins per cluster and 29 nm cluster radius with the first approach and 3.1 ± 0.1 proteins per cluster and 32 ± 2 nm cluster radius with the second approach. Thus, results between the two approaches are in a good agreement. This concept is further supported by MC simulations with different random seeds (Fig. S10).

**
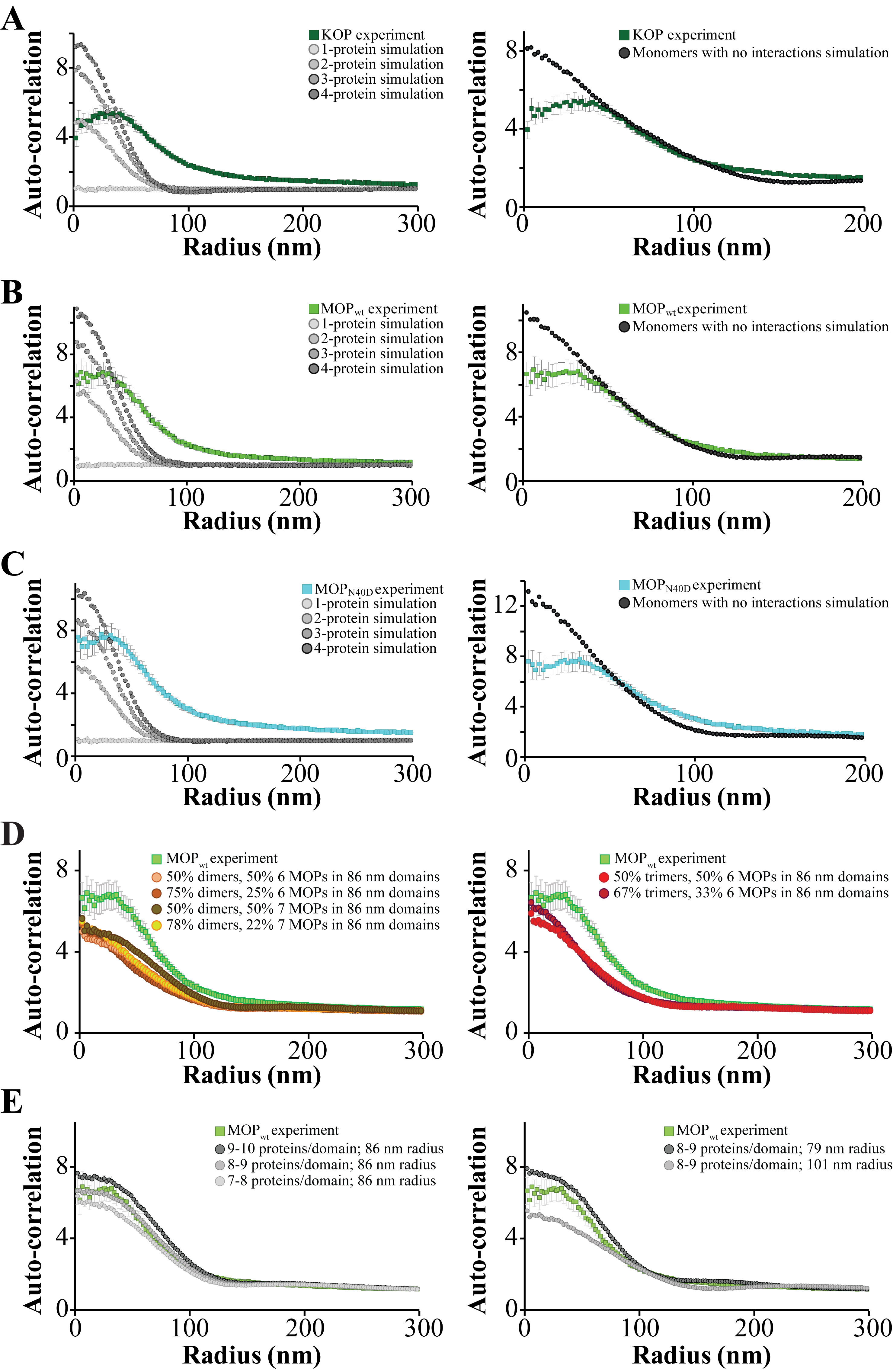
**

**Figure S9**. **Monte Carlo simulations without organized nano-domains do not resemble experimental results. A-C** Simulated images of proteins organized into random tight domains (oligomers, left panel) and random monomers in larger domains (right panel) do not match experimental data and follow a single exponential auto-correlation function. For simulations presented in the left panel, proteins were distributed randomly inside the domains without exclusion area; the protein occupancy/domain size indicated in Table 1 was used. Results are shown for: **A.** KOP; **B.** MOP_wt_; and **C.** MOP_N40D_**.** **D.** Combination of tight oligomers (dimers and trimmers) with proteins distributed in larger domain does not fit experimental data. F-test and S-values are shown in Table S2. **E.** Small changes in number of proteins or domain size have a profound effect on correlation curves. F-test and S-values are shown in Table S2. All simulations were computed using photon count distributions, point spread functions, average localization precisions, average number of appearances, and density of appearances obtained from experimental data.


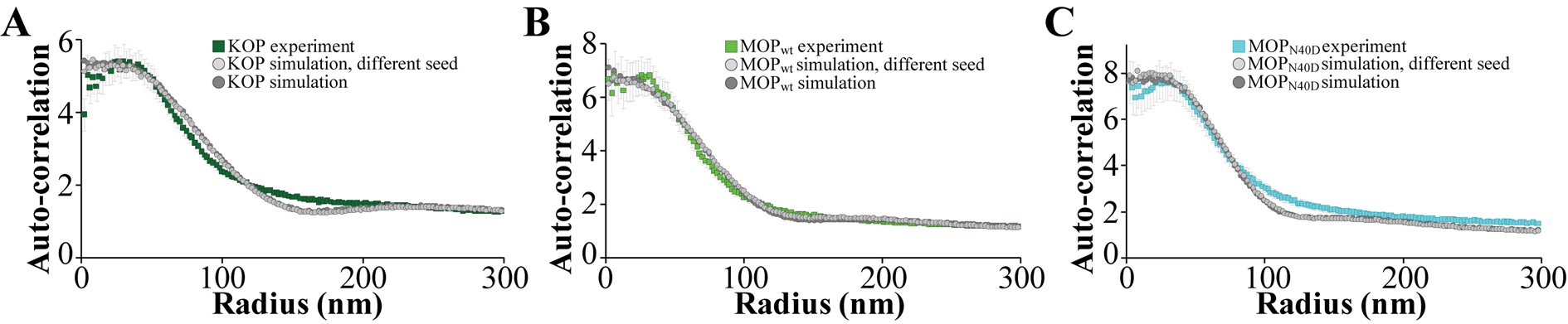


Figure S10. Monte Carlo simulations are robust to different random seeds. Consistent results are obtained for all opioid receptors: A. KOP; B. MOP_wt_; and C. MOP_N40D_.


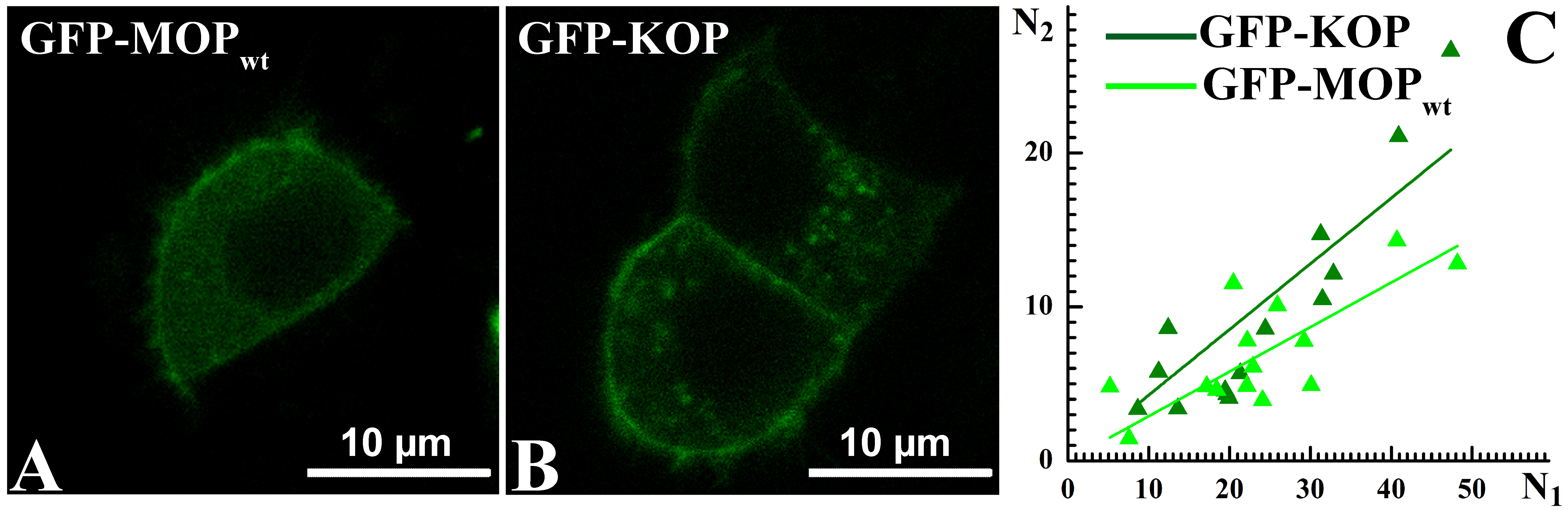


Figure S11. Opioid receptor lateral dynamics in the plasma membrane is not significantly altered by fluorescent protein tag localization. CLSM images of PC12 cells transiently transformed to express N-terminally labeled opioid receptors: A. GFP-MOP_wt_ and B. GFP-KOP. C. The number of molecules (N_2_) characterized by the long diffusion time (τ_D2_) as a function of the number of molecules (N_1_) characterized by the shorter diffusion time (τ_D1_). Points indicate measurements on individual cells. Slopes of the fitted lines show the fraction of molecules characterized by slow diffusion, which is (0.4 ± 0.1) for GFP-KOP and (0.3 ± 0.1) for GFP-MOP_wt_. Differences between the relative contribution of the second component, (1-*y*), were statistically significant for KOP and MOP_wt_, as evident from the two-tail P values: P < 0.05.

**References**

1. Lim S, Becker A, Zimmer A, Lu J, Buettner R, Kirfel J. SNAI1-mediated epithelial-mesenchymal transition confers chemoresistance and cellular plasticity by regulating genes involved in cell death and stem cell maintenance. PloS one 2013;8(6):e66558.

2. Stan D, Calin M, Manduteanu I, Pirvulescu M, Gan AM, Butoi ED, Simion V, Simionescu M. High glucose induces enhanced expression of resistin in human U937 monocyte-like cell line by MAPK- and NF-kB-dependent mechanisms; the modulating effect of insulin. Cell and tissue research 2011;343(2):379-387.

3. Fassnacht M, Weismann D, Ebert S, Adam P, Zink M, Beuschlein F, Hahner S, Allolio B. AKT is highly phosphorylated in pheochromocytomas but not in benign adrenocortical tumors. The Journal of clinical endocrinology and metabolism 2005;90(7):4366-4370.

4. Pan T, Sun J, Hu J, Hu Y, Zhou J, Chen Z, Xu D, Xu W, Zheng S, Zhang S. Cytohesins/ARNO: the function in colorectal cancer cells. PloS one 2014;9(3):e90997.

5. Chen QW, Edvinsson L, Xu CB. Role of ERK/MAPK in endothelin receptor signaling in human aortic smooth muscle cells. BMC cell biology 2009;10:52.

6. Gertsch J, Leonti M, Raduner S, Racz I, Chen JZ, Xie XQ, Altmann KH, Karsak M, Zimmer A. Beta-caryophyllene is a dietary cannabinoid. Proceedings of the National Academy of Sciences of the United States of America 2008;105(26):9099-9104.

7. Mark JK, Aubin RA, Smith S, Hefford MA. Inhibition of mitogen-activated protein kinase phosphatase 3 activity by interdomain binding. The Journal of biological chemistry 2008;283(42):28574-28583.

8. Chiron D, Pellat-Deceunynck C, Amiot M, Bataille R, Jego G. TLR3 ligand induces NF-{kappa}B activation and various fates of multiple myeloma cells depending on IFN-{alpha} production. Journal of immunology 2009;182(7):4471-4478.

9. Nassirpour R, Bahima L, Lalive AL, Luscher C, Lujan R, Slesinger PA. Morphine- and CaMKII-dependent enhancement of GIRK channel signaling in hippocampal neurons. The Journal of neuroscience : the official journal of the Society for Neuroscience 2010;30(40):13419-13430.

10. Manzke T, Niebert M, Koch UR, Caley A, Vogelgesang S, Hulsmann S, Ponimaskin E, Muller U, Smart TG, Harvey RJ, Richter DW. Serotonin receptor 1A-modulated phosphorylation of glycine receptor alpha3 controls breathing in mice. The Journal of clinical investigation 2010;120(11):4118-4128.

11. Tobin SJ, Cacao EE, Hong DW, Terenius L, Vukojevic V, Jovanovic-Talisman T. Nanoscale effects of ethanol and naltrexone on protein organization in the plasma membrane studied by photoactivated localization microscopy (PALM). PloS one 2014;9(2):e87225.

12. Kan L, Mutso AA, McGuire TL, Apkarian AV, Kessler JA. Opioid signaling in mast cells regulates injury responses associated with heterotopic ossification. Inflammation research : official journal of the European Histamine Research Society [et al] 2014;63(3):207-215.

13. Pichler A, Zelcer N, Prior JL, Kuil AJ, Piwnica-Worms D. In vivo RNA interference-mediated ablation of MDR1 P-glycoprotein. Clinical cancer research : an official journal of the American Association for Cancer Research 2005;11(12):4487-4494.

14. Strasner AB, Natarajan M, Doman T, Key D, August A, Henderson AJ. The Src kinase Lck facilitates assembly of HIV-1 at the plasma membrane. Journal of immunology 2008;181(5):3706-3713.

15. Tarassishin L, Loudig O, Bauman A, Shafit-Zagardo B, Suh HS, Lee SC. Interferon regulatory factor 3 inhibits astrocyte inflammatory gene expression through suppression of the proinflammatory miR-155 and miR-155*. Glia 2011;59(12):1911-1922.

16. Zheng YS, Zhang H, Zhang XJ, Feng DD, Luo XQ, Zeng CW, Lin KY, Zhou H, Qu LH, Zhang P, Chen YQ. MiR-100 regulates cell differentiation and survival by targeting RBSP3, a phosphatase-like tumor suppressor in acute myeloid leukemia. Oncogene 2012;31(1):80-92.

17. Gach K, Piestrzeniewicz M, Fichna J, Stefanska B, Szemraj J, Janecka A. Opioid-induced regulation of mu-opioid receptor gene expression in the MCF-7 breast cancer cell line. Biochemistry and cell biology = Biochimie et biologie cellulaire 2008;86(3):217-226.

18. Lu J, Liu Z, Zhao L, Tian H, Liu X, Yuan C. In vivo and in vitro inhibition of human liver cancer progress by downregulation of the mu-opioid receptor and relevant mechanisms. Oncology reports 2013;30(4):1731-1738.

19. Hölt V, Seizinger BR, Garzón J, Loh HH. Receptor Selectivities of the Three Opioid Peptide Families. In: Koch G, Richter D, editors. Biochemical and Clinical Aspects of Neuropeptides Synthesis, Processing, and Gene Structure. Orlando, Florida, USA: Academic Press Inc; 1983. p. 59-72.

20. Vukojevic V, Ming Y, D'Addario C, Hansen M, Langel U, Schulz R, Johansson B, Rigler R, Terenius L. Mu-opioid receptor activation in live cells. FASEB journal 2008;22(10):3537-3548.

21. Li JG, Chen C, Liu-Chen LY. N-Glycosylation of the human kappa opioid receptor enhances its stability but slows its trafficking along the biosynthesis pathway. Biochemistry 2007;46(38):10960-10970.

22. Bond C, LaForge KS, Tian M, Melia D, Zhang S, Borg L, Gong J, Schluger J, Strong JA, Leal SM, Tischfield JA, Kreek MJ, Yu L. Single-nucleotide polymorphism in the human mu opioid receptor gene alters beta-endorphin binding and activity: possible implications for opiate addiction. Proceedings of the National Academy of Sciences of the United States of America 1998;95(16):9608-9613.

23. Beyer A, Koch T, Schroder H, Schulz S, Hollt V. Effect of the A118G polymorphism on binding affinity, potency and agonist-mediated endocytosis, desensitization, and resensitization of the human mu-opioid receptor. Journal of Neurochemistry 2004;89(3):553-560.

24. Kroslak T, Laforge KS, Gianotti RJ, Ho A, Nielsen DA, Kreek MJ. The single nucleotide polymorphism A118G alters functional properties of the human mu opioid receptor. J Neurochem 2007;103(1):77-87.

25. Marchese A, Paing MM, Temple BR, Trejo J. G protein-coupled receptor sorting to endosomes and lysosomes. Annual review of pharmacology and toxicology 2008;48:601-629.

26. Vukojevic V, Heidkamp M, Ming Y, Johansson B, Terenius L, Rigler R. Quantitative single-molecule imaging by confocal laser scanning microscopy. Proceedings of the National Academy of Sciences of the United States of America 2008;105(47):18176-18181.

27. Müller CB, Loman A, Pacheco V, Koberling F, Willbold D, Richtering W, Enderlein J. Precise measurement of diffusion by multi-color dual-focus fluorescence correlation spectroscopy. EPL (Europhysics Letters) 2008;83(4):46001.

28. Haupts U, Maiti S, Schwille P, Webb WW. Dynamics of fluorescence fluctuations in green fluorescent protein observed by fluorescence correlation spectroscopy. Proceedings of the National Academy of Sciences of the United States of America 1998;95(23):13573-13578.

29. Elson EL. Fluorescence correlation spectroscopy measures molecular transport in cells. Traffic 2001;2(11):789-796.

30. Bacia K, Petrasek Z, Schwille P. Correcting for spectral cross-talk in dual-color fluorescence cross-correlation spectroscopy. Chemphyschem : a European journal of chemical physics and physical chemistry 2012;13(5):1221-1231.

31. Persson G, Thyberg P, Widengren J. Modulated fluorescence correlation spectroscopy with complete time range information. Biophysical journal 2008;94(3):977-985.

32. Lauer S, Goldstein B, Nolan RL, Nolan JP. Analysis of cholera toxin-ganglioside interactions by flow cytometry. Biochemistry 2002;41(6):1742-1751.

33. Moran-Mirabal JM, Edel JB, Meyer GD, Throckmorton D, Singh AK, Craighead HG. Micrometer-sized supported lipid bilayer arrays for bacterial toxin binding studies through total internal reflection fluorescence microscopy. Biophysical journal 2005;89(1):296-305.

34. Rissanen S, Grzybek M, Orlowski A, Rog T, Cramariuc O, Levental I, Eggeling C, Sezgin E, Vattulainen I. Phase Partitioning of GM1 and Its Bodipy-Labeled Analog Determine Their Different Binding to Cholera Toxin. Frontiers in physiology 2017;8:252.

35. Shi J, Yang T, Kataoka S, Zhang Y, Diaz AJ, Cremer PS. GM1 clustering inhibits cholera toxin binding in supported phospholipid membranes. Journal of the American Chemical Society 2007;129(18):5954-5961.

36. Krishnan P, Singla A, Lee C-A, Weatherston JD, Worstell NC, Wu H-J. Hetero-multivalent binding of cholera toxin subunit B with glycolipid mixtures. Colloids and Surfaces B: Biointerfaces 2017;160:281-288.

37. Kuziemko GM, Stroh M, Stevens RC. Cholera toxin binding affinity and specificity for gangliosides determined by surface plasmon resonance. Biochemistry 1996;35(20):6375-6384.

38. Sengupta P, Jovanovic-Talisman T, Skoko D, Renz M, Veatch SL, Lippincott-Schwartz J. Probing protein heterogeneity in the plasma membrane using PALM and pair correlation analysis. Nature methods 2011;8(11):969-975.

39. Betzig E, Patterson GH, Sougrat R, Lindwasser OW, Olenych S, Bonifacino JS, Davidson MW, Lippincott-Schwartz J, Hess HF. Imaging intracellular fluorescent proteins at nanometer resolution. Science 2006;313(5793):1642-1645.

40. Sengupta P, Jovanovic-Talisman T, Lippincott-Schwartz J. Quantifying spatial organization in point-localization superresolution images using pair correlation analysis. Nature protocols 2013;8(2):345-354.

41. van Zanten TS, Cambi A, Koopman M, Joosten B, Figdor CG, Garcia-Parajo MF. Hotspots of GPI-anchored proteins and integrin nanoclusters function as nucleation sites for cell adhesion. Proceedings of the National Academy of Sciences of the United States of America 2009;106(44):18557-18562.

42. Goswami D, Gowrishankar K, Bilgrami S, Ghosh S, Raghupathy R, Chadda R, Vishwakarma R, Rao M, Mayor S. Nanoclusters of GPI-anchored proteins are formed by cortical actin-driven activity. Cell 2008;135(6):1085-1097.

43. Zagouras P, Rose JK. Dynamic equilibrium between vesicular stomatitis virus glycoprotein monomers and trimers in the Golgi and at the cell surface. Journal of virology 1993;67(12):7533-7538.

44. Lubelski A, Sokolov IM, Klafter J. Nonergodicity mimics inhomogeneity in single particle tracking. Physical review letters 2008;100(25):250602.

45. Nicholas J. Giordano JN, Nakanishi H. Computational Physics. 2nd ed: Addison-Wesley; 2005.

46. Bates, D. M. , Watts DG. Nonlinear regression analysis and its applications. New York: Wiley; 1988.
