# Supplementary material for: Dynamic lateral organization of opioid receptors (kappa, muwt and muN40D) in the plasma membrane at the nanoscale level
Source: Traffic. 2018 Jun 21;19(9):690–709. doi: 10.1111/tra.12582 (PMC6120469; doi:10.1111/tra.12582)
Supplement: Supplementary file 1 — Editorial Process [file TRA-19-690-s002.pdf]

Title: **Dynamic lateral organization of opioid receptors (kappa, mu$_{wt}$ and mu$_{N40D}$) in the plasma membrane at the nanoscale level**

Authors: Maciej K. Rogacki, Ottavia Golfetto, Steven J. Tobin, Tianyi Li, Sunetra Biswas, Raphael Jorand, Huiying Zhang, Vlad Radoi, Yu Ming, Per Svenningsson, Daniel Ganjali, Devin L. Wakefield, Athanasios Sideris, Alexander R. Small, Lars Terenius, Tijana Jovanović-Talisman, Vladana Vukojević

Article Type: Original Research

Monitoring Editor	Trina Schroer
Date Submitted	2 February 2016
Date for Decision 1	1 April 2016
Date Resubmitted	27 December 2016
Date for Decision 2	24 January 2017
Date Resubmitted	2 November 2017
Date for Decision 3	12 December 2017
Date Resubmitted	30 March 2018
Date for Decision 4	23 April 2018
Accepted	18 May 2018

Decision and Reviews

Dear Dr. Vikojevic

Thank you for submitting your manuscript "Lateral organization of opioid receptor variants (kappa, muwt and muN40D) in the plasma membrane at the nanoscale level" for consideration for publication in Traffic. I asked two colleagues who are experts in the field to review this paper and their verbatim comments are appended below. Although their reasons differ, neither referee is supportive of publication of this paper. Referee 1 is of the view that your conclusions are not supported by the data presented and that a significant number of additional experiments would be needed to address his/her concerns. Referee 2 notes that your results do not provide the kind of conceptual or mechanistic advance that we aim for in the papers that are published in Traffic. Given the nature of these comments I must reject this paper. I am sorry that I do not have better news for you, but hope you find the detailed recommendations of the referees useful as your work progresses.

Sincerely,

Trina A. Schroer, Ph.D.
Co-Editor

Referee's Comments to the Authors

Referee: 1

Comments to the Author
This manuscript submitted by Rogacki et al applies various single molecule techniques (FCS and PALM) to study the lateral heterogeneity of opioid receptors in the plasma membrane. While the manuscript highlights results that are both potentially interesting and biological significant, I have major concerns about multiple aspects of the experimental methods and analysis. These concerns have significant implications for the biological interpretation of the results. For instance, the authors claim to measure two diffusion times, one for transient interactions and other

being free diffusion. However, I would argue that the results are not as clear-cut as claimed. Further, even the finding regarding how cholesterol influences opioid receptor organization is not conclusively shown. This undermines the model proposed in figure 6. Furthermore, although PALM experiments have been used to determine the density and protein organization of the opioid receptors and its mutants, the possibility of high heterogeneity may be ameliorated due to averaging and the results may not be as distinctive as discussed. In my opinion, the authors need to iron out these and other issues detailed below before concluding a significant portion of their biological claims.

Major Concerns

1. It is difficult to compare the FCS results across figures because the authors have not provided the results in a form that can be easily assessed. A table should be included that lists the values obtained from each set of experiments. The correlation times should also be converted into diffusion coefficients. This is essential to be able to compare the results with other measurements in the literature as well as across experiments. These comparisons are also important because the temporal autocorrelation curves for live cells look different across experiments, as in Figure 1B and 1E. I tried overlaying the two figures and the second decay constant is much higher for 1E. This becomes even more problematic when I look at the subsequent figures, say figure 2. The autocorrelation functions here are completely different from figure 1. Figure 2B shows that the autocorrelation function of MOP-WT is completely decayed within 10ms, while figure 1B has it extending till 100ms, an order of magnitude more. My guess is that the labels in figure 2B are switched and the text is probably correct (cholesterol is more mobile). However, Figure 2C seems very different from others.

2. On a related note, currently, many of the conclusions from the FCS measurements seem to be based on qualitative comparisons. The authors should clearly describe what statistical analyses were performed and which tests were used. They should also provide additional information about errors introduced by averaging autocorrelation curves across cells. For instance, take figure 2C. The authors have interpreted the effect of cholesterol sequestration for the different proteins. Since the difference between the untreated and treated appears to be at the maximum a factor of 2 or 3, one could imagine it may remain in the confidence interval of the curve. Without statistical tests, it would not be prudent to derive conclusions from these experiments. In my opinion, every one of the autocorrelation data should include this for interpreting the results.

3. For both the cross correlation and cholesterol depletion experiments, a positive control which shows the sensitivity of the measurement is needed, eg. cholera toxin B and GM1 ganglioside for cross correlation measurements is necessary.

4. In figure 3, I would have preferred if the authors have directly used GM1 instead of using the proxy, since CTxB binding can cause reorganization of membrane domains. Also, there appears to be significant colocalization between receptor and CTxB in the confocal images. Of course, cross-correlation is a superior technique to diffraction limited colocalization, but it is also brings us back to the previous comment 3, in that it is equally likely that the cross correlation itself could be noisy or the measurement or analysis not sensitive enough and this is the reason for the absence of signal. Cross correlation will also not be sensitive to immobile pools of molecules, a possibility that is not mentioned here.

5. The comparison of the FCS and PALM data would be more compelling if they were done in the same cell line and similar treatments were performed for the PALM and FCS experiments.

6. A major issue with using PALM as a quantification tool is that there are several artifacts that creep into the measurement. I commend the authors for performing several important controls and simulations that alleviate most of these. However, there are a few others that I think are important, especially with respect to undercounting of molecules. Undercounting of molecules could be due to maturation and other missed molecules (eg. Xan et al PNAS, Punchner et al PNAS) should be mentioned. More importantly, the choice of PA-GFP is puzzling to the reviewer. It has been shown by the Lakadamyali group (Durisic et al Nature Methods 2014) that PA-GFP has one of the worst "photoactivation efficiency" which leads to significant underestimation of stoichiometry. The authors should address this with regards to an estimate of how much their numbers would be off. A calibration control in the absence of blinking should theoretically be 1. Alternatively, it would be a good control to determine autocorrelation function of a protein that does not cluster in the membrane.

7. The authors have averaged the autocorrelation function of ~15 cells and 40 measurements for the PALM measurement. This would average out any cell-cell variation and would also neglect the distribution of the spatial organization of the proteins that could be important. Would it be possible for the authors to use individual

autocorrelation functions per cell or measurement and then determine density of protein? I suspect that this would greatly increase the error bars of the figure 5. Also, how much will the results vary if instead of using the average localization precision, you were to use the localization precision corresponding to each reconstructed image? What are the standard deviations of the localization measurements in the different PALM images?

8. Finally, from a biological perspective, the PALM data would be more informative if they were coupled to some simple colocalization analyses by confocal microscopy to test whether the receptor clusters detected here correspond to known structures such as clathrin coated pits or caveolae.

Minor Comments
1. The authors should state whether the cells were bleached prior to the FCS measurements. This is important because if the tagged protein is readily visible by confocal imaging (as appears to be the case in the images shown here), the expression levels are often too high to perform FCS.
2. The authors should ensure the colors for figures are consistent. Take for instance, in figure 1B and 1E, data for KOP is shown in red in one panel and green in another. This could potentially be confusing to the reader.
3. It would helpful if the authors were to speculate on why the slopes in figure 1C are different for the different proteins.
4. In figure S2, the authors should clarify whether the same fields of cells were imaged before and after addition of ligands. The cells seem to be the same in A/D and C/F.
5. Also in regard to figure S2, the authors should comment on how the ligand is being internalized in panel F since it does not colocalize with the tagged receptor.
6. What are the levels of the proteins in stably expressing PC-12 compared with other cell lines in S4?
7. Everywhere in the text, use superscript for µm2
8. A few important references with regards to PALM have not been mentioned.
a) Annibale et al PloS One 2011
b) Nan et al PNAS 2013
c) Gunzenhäuser et al Nano Letters 2012
d) Sengupta et al Nature Proc 2013
e) Lee et al PNAS 2012

Referee: 2

Comments to the Author
This paper uses FCS and PALM to estimate the lateral organization of KOR, MOR, and the MORN40D mutant. Based on time constants of autocorrelation curves and PALM data, the authors state a very broad model that receptors are organized in domains, are excluded from GM1 domains, and are organized in cholesterol domains.

It is difficult to put these findings in perspective, considering previously published data. The idea that cholesterol is involved in organization of membrane proteins including GPCRs such as opioid receptors is quite well established. While the argument that "quantitative and nondestructive methods are urgently needed to characterize under minimally invasive conditions the capacity of drug candidates to fine-tune receptor functions by harvesting this dynamic regulatory mechanism" in the context of allosteric modulators might be valid, this manuscript does not address this argument. There are a wide variety of agonists, antagonists, and allosteric modulators available, and if the authors can pick a few relevant ones and test opioid receptors by FCCS and PALM, this might be an opportunity to test a focused hypothesis and learn something new.

Alternatively, if the authors intend this as a methods paper, they need to be more rigorous in their controls.

A major concern is that receptors are tagged on the C-termini, this might cause them to behave differently from endogenous receptors. Most of the cells (e.g., Fig S2) show substantial ER fluorescence, which suggests that they are either overexpressed or are poorly folded. This is also compounded by the fact that MORN40D does not internalize, which suggests that the receptor might be altered. For example, KOR has a PDZ ligand on its C-terminus, and there is previous evidence that disrupting PDZ interactions can change diffusion kinetics of GPCRs (e.g., Valentine and Haggie, 2011).

The goodness of fit should be shown for the curves individually. While the curve might fit best to three decay times, how much worse is it when fit to two phases or a single phase curve?

The reason for ignoring the fast decay time is not explained well, especially as the data are not shown. What fraction of the total falls within this decay time? If they use a soluble eGFP, do they get just the fast decay time?

PC12 cells express endogenous opioid receptors, and also might secrete dynorphins and other opioid agonists, and it is not clear if these complicate the results.

Author Rebuttal

Dear Dr. Schroer,

Thank you for considering our manuscript "Lateral organization of opioid receptor variants (kappa, muwt and muN40D) in the plasma membrane at the nanoscale level" (TRA-16-0483) for publication in Traffic and for giving us the opportunity to revise it. We are grateful to the Reviewers for their constructive criticism and valuable suggestions. We have now conducted the additional experiments that were recommended by the Reviewers and revised our manuscript in accordance with their suggestions. Consequently, we have reorganized the presentation of our experimental results, focusing on our findings about opioid receptor organization; we have, in accordance with these changes, modified the text, and have included numerous controls. We provide a detailed explanation for the few points that we did not change, and we support our arguments with scientific literature and additional data.

We have also made the following changes in the Authors' list. Mr. Tobin is now third author, as he did significant amount of additional PALM experiments on cholesterol depletion. Dr. Wakefield was included as an author. He worked with Mr. Tobin to acquire and analyze TIRF images and contributed with PALM data analysis. MSc Vlad Radoi was included as an author as he performed additional FCS and FCCS experiments.

Please find enclosed below a detailed description of the changes made and the response to the Reviewers.

Referee: 1

Comments to the Author
This manuscript submitted by Rogacki et al applies various single molecule techniques (FCS and PALM) to study the lateral heterogeneity of opioid receptors in the plasma membrane. While the manuscript highlights results that are both potentially interesting and biological significant, I have major concerns about multiple aspects of the experimental methods and analysis. These concerns have significant implications for the biological interpretation of the results. For instance, the authors claim to measure two diffusion times, one for transient interactions and other being free diffusion. However, I would argue that the results are not as clear-cut as claimed. Further, even the finding regarding how cholesterol influences opioid receptor organization is not conclusively shown. This undermines the model proposed in figure 6. Furthermore, although PALM experiments have been used to determine the density and protein organization of the opioid receptors and its mutants, the possibility of high heterogeneity may be ameliorated due to averaging and the results may not be as distinctive as discussed. In my opinion, the authors need to iron out these and other issues detailed below before concluding a significant portion of their biological claims.

Major Concerns
1. It is difficult to compare the FCS results across figures because the authors have not provided the results in a form that can be easily assessed. A table should be included that lists the values obtained from each set of experiments. The correlation times should also be converted into diffusion coefficients. This is essential to be able to compare the results with other measurements in the literature as well as across experiments. These comparisons are also important because the temporal autocorrelation curves for live cells look different across experiments, as in Figure 1B and 1E. I tried overlaying the two figures and the second decay constant is much higher for 1E. This becomes even more problematic when I look at the subsequent figures, say figure 2. The autocorrelation functions here are completely different from figure 1. Figure 2B shows that the autocorrelation function of MOP-WT is completely decayed within 10ms, while figure 1B has it extending till 100ms, an order of magnitude more. My guess is that the labels in figure 2B are switched and the text is probably correct (cholesterol is more mobile). However, Figure 2C seems very different from others.

We thank the Reviewer for the constructive criticism and valuable suggestions for improvement. To begin with, we thank the Reviewer for noticing the typographical error in the old Fig. 2B and we sincerely apologize for this oversight,

which we have corrected in the revised manuscript. Thus, the Reviewer is correct in remarking that the labels on old Fig. 2B were swapped (cholesterol is indeed more mobile). Hence, the critique that: “Figure 2B shows that the autocorrelation function of MOP-WT is completely decayed within 10 ms, while figure 1B has it extending till 100 ms, an order of magnitude more.” does not stand and is due to this unfortunate mislabeling error.

The Reviewer states that: “the temporal autocorrelation curves for live cells look different across experiments, as in Figure 1B and 1E. I tried overlaying the two figures and the second decay constant is much higher for 1E.” For KOP, the green tACC in old Fig. 1E (which shows the tACC recorded on one cell, now Fig S5A, dark green), agrees well with the corresponding red ACC in old Fig. 1B (which shows an average of 10 cells, now Fig 1E, dark green). Moreover, the ACC presented in old Fig. 1B (now Fig. 1F) and in old Fig. 2C (now Fig. 4C) consistently show that the relative contribution of the second component is largest for KOP, intermediate for MOPwt and the lowest for MOPN40D.

Having said this, we recognize that color labeling in old Figs 1B and 1F may have been misleading. We have now corrected this, and we use throughout the manuscript cyan for MOPN40D, green for MOPwt, and dark green for KOP. Moreover, we have included numerical values in Table S1, as the Reviewer has suggested, and we comment on the consistency of diffusion times across experiments in the Supplementary Methods: FCS and FCCS (page 3, second paragraph), where we have added the following text: “Not surprisingly, average values of characteristic decay times (τD1 and τD2) and their corresponding relative amplitudes (y and (1 – y)) differed somewhat between different series of experiments (Table S1), subject to cell batch and passage number in culture, but the overall trend within the same series of experiments was always the same, with KOP showing the largest relative contribution of the second component, (1 - y), whereas corresponding values were lowest for MOPN40D (Table S1). Also, standard deviation between measurements recorded on the same cell was smaller than the standard deviation of measurements between different cells. See for example the closely overlapping tACC normalized to the same amplitude shown in Fig. S4B (bottom), and the values given in Table S1.”

Please note that in addition to changing colors, we have also changed figure order. Thus, former Fig. 1C is now Fig. 1F; former Fig. 1E, which is now Fig. S5A, was expanded to include data for eGFP in the cytoplasm of live cells; we have also provided a representative tACC for MOPwt (Fig. 1D) and for Rhodamine 6G (Fig. S4D).

Moreover, the Reviewer suggests that: “The correlation times should also be converted into diffusion coefficients.” While we do agree with the Reviewer that this is routinely done in the literature, extraction of diffusion coefficients may not be optimal here. Chemical reactions and lateral organization of the receptors, i.e. their exclusion from ganglioside GM1-enriched domains affect the apparent diffusion behavior of molecules, making it difficult to extract diffusion coefficients from diffusion times. We discuss this point in the Supplementary Methods: FCS and FCCS (page 3, third paragraph), where we point out that if receptor molecules are transiently trapped in domains, this would be reflected in FCS as longer diffusion times because the receptor is temporarily “stalled” when associated with domains. Conversely, if the receptor is excluded from domains, the area that is accessible by free diffusion is smaller than the total area that is determined by FCS calibration measurements, which are typically performed in solution. This would be reflected in FCS as shorter diffusion times, but without implying faster mobility.”

The comparative information that the Reviewer is requesting may be easily extracted from calibration measurements using Rhodamine 6G shown in Fig. S4D. In our system, the lateral diffusion time for Rh6G is τRh6G = (27 $\pm$ 3) μs. Using the diffusion coefficient of Rh6G, DRh6G = 4.14$\times$10-10 m2s-1 (Muller et al. Optics express 2008 16:4322), one can calculate that an apparent diffusion time of 10 ms yields under ideal conditions an apparent diffusion coefficient of D = 1.1$\times$10-12 m2s-1.

2. On a related note, currently, many of the conclusions from the FCS measurements seem to be based on qualitative comparisons. The authors should clearly describe what statistical analyses were performed and which tests were used. They should also provide additional information about errors introduced by averaging autocorrelation curves across cells. For instance, take figure 2C. The authors have interpreted the effect of cholesterol sequestration for the different proteins. Since the difference between the untreated and treated appears to be at the maximum a factor of 2 or 3, one could imagine it may remain in the confidence interval of the curve. Without statistical tests, it would not be prudent to derive conclusions from these experiments. In my opinion, every one of the autocorrelation data should include this for interpreting the results.

We thank the reviewer for this comment. We have included in the revised text information on statistical analysis. In particular, we have used paired sample t-test to compare the population means, i.e. to analyze the relative contribution of the second component, (1 – y), across experiments. We have given in Table S1 the standard deviation

for all FCS curves presented in this work and in the footnote to Table S1 the two-tailed P values. We have also included parts of this relevant information in the main text (page 3, left column, last paragraph).

3. For both the cross correlation and cholesterol depletion experiments, a positive control which shows the sensitivity of the measurement is needed, eg. cholera toxin B and GM1 ganglioside for cross correlation measurements is necessary.

We have performed dual-color FCCS analysis of CtxB Alexa Fluor® 647 interactions with BODIPY® FL C5-ganglioside GM1 using untransformed PC12 cells and measured the extent of these interactions also in the cell culture medium above the cells. We show the results obtained in the cell culture medium in Fig. S7 A-C, and the obtained relative cross-correlation amplitudes (RCCA) in Fig. S8.

As for cholesterol depletion, we are not aware of any good control experiments that would unequivocally demonstrate the extent of cholesterol depletion/enrichment. We have used in another study cholesterol oxidase to deplete the cellular pool of cholesterol and obtained similar results as with MβCD (Lundius et. al. J. Biol. Chem. 2014, 289:4660-4673).

4. In figure 3, I would have preferred if the authors have directly used GM1 instead of using the proxy, since CTxB binding can cause reorganization of membrane domains. Also, there appears to be significant colocalization between receptor and CTxB in the confocal images. Of course, cross-correlation is a superior technique to diffraction limited colocalization, but it is also brings us back to the previous comment 3, in that it is equally likely that the cross correlation itself could be noisy or the measurement or analysis not sensitive enough and this is the reason for the absence of signal. Cross correlation will also not be sensitive to immobile pools of molecules, a possibility that is not mentioned here.

Given the robust autocorrelation curves in individual channels and the fact that there is one pinhole in front of the two APD detectors in the ConfoCor 3 system, we don't expect the cross-correlation curve in old Fig. 3, now Fig. 5 A, to be unpredictably noisy. We have, however, followed the Reviewer's suggestion and performed additional, more direct control experiments. To this goal, we have developed PC12 cells that stably express MOPwt-Tomato and have treated these cells with green-fluorescent BODIPY® FL C5-ganglioside GM1. These results are shown in Fig. S7 D and E and in Fig. S8.

We have also performed TIRF experiments in COS-7 cells (Figs 5B and S12), where we similarly observe that most cells where opioid receptor expression is high (green) do not stain extensively with the ganglioside GM1 marker CtxB Alexa Fluor® 647 (red), as evident by imaging and the Scatter plots. At the same time, most cells with low opioid receptor expression, i.e. low green signal, readily stain in red. This suggests that also in COS-7 cells opioid receptors do not significantly co-localize, i.e. are largely excluded from GM1 ganglioside-enriched domains.

As to the comment on membrane domain reorganization due to the action of CtxB, we refer to a recently published study showing that: "CtxB does not alter the organization of the plasma membrane in a way that influences the diffusion of other molecules" (Day CA, Kenworthy AK. Mechanisms underlying the confined diffusion of cholera toxin B-subunit in intact cell membranes. PLoS One. 2012 7(4):e34923. doi: 10.1371/journal.pone.0034923). We also point out here that the autocorrelation curve for KOP-eGFP (dark green) shows similar characteristic decay times as observed in Fig 1.

Finally, we entirely agree with the Reviewer's comment that: "Cross correlation will also not be sensitive to immobile pools of molecules, a possibility that is not mentioned here." This is indeed a general limitation of FCS that we have failed to bring up in our original manuscript. We have now explicitly outlined this limitation of FCS in the Discussion (page 5, left column, second paragraph) where we write: "In addition, FCS cannot account for proteins associated with large immobile structures, which contribute to the overall background but do not give rise to fluorescence intensity fluctuations".

5. The comparison of the FCS and PALM data would be more compelling if they were done in the same cell line and similar treatments were performed for the PALM and FCS experiments.
While we do agree with the Reviewer that it would have been thorough to provide FCS and PALM data on both cell lines, this would not be practical for the following reasons. First, performance of this extensive set of experiments in each laboratory on two different cell lines would be repetitive and time and resource consuming. Secondly, the use of PC12 cells for PALM studies was complicated due to low coverslip adhesion and clumping of PC12 cells, which could

therefore not be effectively used for total internal reflection (TIRF) illumination. We have therefore regarded that it is best to perform measurements on different cell lines, which were chosen to best suit the methods used, and compare the results. As we present here, we have obtained by PALM in COS-7 cells results that are congruent with the results obtained by FCS in PC12 cells. We therefor argue that the fact that we have obtained consistent data in two different laboratories, with two different techniques, and in two different cell lines in a steady state and upon cholesterol depletion, compellingly suggests that this may be a general phenomenon.

6. A major issue with using PALM as a quantification tool is that there are several artifacts that creep into the measurement. I commend the authors for performing several important controls and simulations that alleviate most of these. However, there are a few others that I think are important, especially with respect to undercounting of molecules. Undercounting of molecules could be due to maturation and other missed molecules (eg. Xan et al PNAS, Punchner et al PNAS) should be mentioned. More importantly, the choice of PA-GFP is puzzling to the reviewer. It has been shown by the Lakadamyali group (Durisic et al Nature Methods 2014) that PA-GFP has one of the worst "photoactivation efficiency" which leads to significant underestimation of stoichiometry. The authors should address this with regards to an estimate of how much their numbers would be off. A calibration control in the absence of blinking should theoretically be 1. Alternatively, it would be a good control to determine autocorrelation function of a protein that does not cluster in the membrane.

paGFP is an excellent monomeric optical highlighter protein with good signal to noise ratio. Durisic et al (and many others) use 405 nm light for paGFP activation. We have optimized our activation and imaging conditions and we use only 488 nm light as described in the methods section (this approach has been also published; for example Sengupta et al. Nat. Methods, 8, 969, 2011). Using careful evaluation of paGFP activation/blinking properties for imaging conditions used in the study (as now described in detail in supplemental methods), we can obtain average number of appearances for paGFP and we include this value in our autocorrelation analysis and density calculations to prevent overcounting. These calibration measurements show random distribution of paGFP with auto-correlation curve at 1 as suggested by the Reviewer. Importantly, we obtained excellent agreement with literature using this approach. For example, we detect trimers for trimeric VSVG protein (supplemental figure S9D; Zagouras et al. J. Virol 1993 67:7533; Sengupta et al. Nat. Methods 2011 8:969). The paGFP-GPI distribution (supplemental figure S9B) is consistent with previously published data obtained using PALM and other biophysical techniques (van Zanten et al. PNAS 2009 106:8557; Goswami et al. Cell 2008 135:1085; Sengupta et al. Nat. Methods 2011 8:969). In addition, some GPI areas shown here exhibit a random distribution as expected from the above-cited literature. Finally, the obtained densities of opioid receptors (ORs) match well with the expected density of GPCRs in cells and previous super-resolution publications (for example Jonas et al. JBC 2015 290:3875). We have included possible issues regarding counting in super-resolution microscopy per reviewer's suggestion in the Discussion and significantly expanded our explanation on imaging and analysis in the main text and in supplemental methods.

7. The authors have averaged the autocorrelation function of ~15 cells and 40 measurements for the PALM measurement. This would average out any cell-cell variation and would also neglect the distribution of the spatial organization of the proteins that could be important. Would it be possible for the authors to use individual autocorrelation functions per cell or measurement and then determine density of protein? I suspect that this would greatly increase the error bars of the figure 5.

We have performed the analysis as suggested by the reviewer and this is now clarified in Methods and Supplemental information. We determine the density of proteins before computing auto-correlation functions. We calculate the density of proteins from each area of each cell, and then average the values and calculate standard error for the density.

Auto-correlation functions were calculated for each area independently to estimate protein organization parameters. As we have demonstrated previously (Tobin et al. Plos ONE 2014 9:e87225, Figure 1C-E), protein organization can be inferred either from averaged autocorrelation curves or from individual correlation curves (results are subsequently averaged). For example, comparison between the two approaches for paGFP-GPI and VSVG-paGFP (Fig. S9) is as follows:

PAGFP-GPI

1) When we compute the auto-correlation function from every image, we estimate the parameters from every image, and then average the parameters, we obtain:

paGFP-GPI: Number of proteins per cluster = 2.9 ± 0.2

Cluster radius (nm) = 37 ± 2

VSVG-paGFP: Number of proteins per cluster = 3.1 ± 0.1

Cluster radius (nm) = 32 ± 2

2) When we compute the auto-correlation function from every image, calculate the average auto-correlation function, and then estimate the parameters from the average, we obtain:
paGFP-GPI: Number of proteins per cluster = 3.1
Cluster radius (nm) = 37
VSVG-paGFP: Number of proteins per cluster = 3.6
Cluster radius (nm) = 29

Importantly, all PALM data is supported by Monte Carlo simulations.
We have expanded our discussion of these issues in the Supplementary Methods, subsection PALM image analysis protocol (page 4) and in Fig. S9.

Also, how much will the results vary if instead of using the average localization precision, you were to use the localization precision corresponding to each reconstructed image?
We use the localization precision for each measurement as suggested by the Reviewer. This is now clearly indicated in the Supplementary Methods, subsection PALM image analysis protocol (page 4).

What are the standard deviations of the localization measurements in the different PALM images?
KOP: (16.5 ± 1.0) nm
MOPwt: (15.8 ± 1.5) nm
MOPN40D: (16.2 ± 1.5) nm
We have included this information on page 3, right column, second paragraph.
8. Finally, from a biological perspective, the PALM data would be more informative if they were coupled to some simple colocalization analyses by confocal microscopy to test whether the receptor clusters detected here correspond to known structures such as clathrin coated pits or caveolae.

While we do agree that this is of interest and intend to do perform such studies in the future, we focus here on differences between the selected opioid receptor subtypes in the steady state.

Minor Comments
1. The authors should state whether the cells were bleached prior to the FCS measurements.

We thank the Reviewer for this comment. We have now included this information in the Materials and Methods section, subsection Optical setup for CLSM and FCS (page 8, left column, second paragraph).

This is important because if the tagged protein is readily visible by confocal imaging (as appears to be the case in the images shown here), the expression levels are often too high to perform FCS.

As explained on page 7, subsection Optical setup for CLSM and FCS, first paragraph, we use a uniquely modified system that allows photon detection with avalanche photodiodes (APDs) in the imaging mode (Vukojević et al. PNAS 2008 105:18176, reference 70 in the manuscript). Given the superior photon detection sensitivity, high quantum efficiency, and a broad spectral response range of APDs as compared to PMTs, which are typically used in CLSM (e.g. Lawrence et al. Imaging, Manipulation, and Analysis of Biomolecules, Cells, and Tissues VI, edited by Daniel L. Farkas, Dan V. Nicolau, Robert C. Leif, Proc. of SPIE Vol. 6859, 68590M, (2008)) reasonably bright images of cells are readily obtained. In order to avoid any confusion, we have replaced the old Fig. 1A with a CLSM image of a low expressing cell, new Fig. 1B. We also point out that the amplitude of the non-normalized tACC in Figs 1D, 4B (green), 5A (green), S4B (middle), S5B (green) and the graph in Fig. 1F clearly show that about $N \approx 4$-30 opioid receptor molecules were detected in the observation volume element (OVE). Cells showing $N > 30$ were not analyzed.

2. The authors should ensure the colors for figures are consistent. Take for instance, in figure 1B and 1E, data for KOP is shown in red in one panel and green in another. This could potentially be confusing to the reader.

We agree with the Reviewer and apologize for problems cussed by inconsistent color coding. We have corrected this throughout.

3. It would helpful if the authors were to speculate on why the slopes in figure 1C are different for the different proteins.

As we have now explained in the caption to Fig. 1, slopes of the fitted lines show the fraction of opioid receptor molecules characterized by slow diffusion, i.e. associated with domains, which is (0.42 ± 0.06) for KOP, (0.30 ± 0.15) for MOPwt and (0.16 ± 0.07) for MOPN40D. These slopes are different because the fraction of opioid receptor molecules that is associated with domains is different for different opioid receptor subtypes. We have discussed this in the Results section (page 3, left column, 10th line from below), and also in relation to the proposed model, Discussion (page 5, right column, paragraphs 2 and 3).

4. In figure S2, the authors should clarify whether the same fields of cells were imaged before and after addition of ligands. The cells seem to be the same in A/D and C/F.

In all three cases shown in Fig. S2, CLSM images were acquired before stimulation (Fig. S2 A-C) and after stimulation (Fig. S2 D-F). We have now clarified this in the figure legend. However, when we stimulate the cells and when massive ligand-induced receptor internalization is observed, the PC12 cells tend to move and turn. This is why the CLSM images at time point zero, t = 0 min, just before ligand addition, are not exactly identical to the images acquired later on. This is more obvious in the case of MOPwt and MOPN40D-expressing cells where higher optical zoom was used.

5. Also in regard to figure S2, the authors should comment on how the ligand is being internalized in panel F since it does not colocalize with the tagged receptor.

In comparison to MOPwt, the MOPN40D variant is lacking a putative N-linked glycosylation site in the N-terminus. Altered N-linked glycosylation was shown to affect receptor trafficking to the cell surface, ligand binding affinity, and the ensuing ligand-induced trafficking cascades in many GPCRs. For example, lack of N-glycosylation in hKOP was shown to increase agonist-induced receptor phosphorylation and affect downstream steps in agonist-induced receptor regulation (Li JG, Chen C, Liu-Chen LY. N-glycosylation of the human kappa opioid receptor enhances its stability but slows its trafficking along the biosynthesis pathway. Biochemistry 46: 10960–10970, 2007). Unfortunately, conflicting results are reported in the literature when it comes to MOPwt and its most commonly encountered variant MOPN40D. While the findings reported in the classical study by Bond et al. (Bond et al. Proc. Natl Acad. Sci. USA 1998 95:9608) showing that these two MOP variants differ about three times in beta-endorphin binding affinity were contested by Beyer et al. (Beyer et al. J Neurochem. 2004 89:553) showing that there are no differences at all between them, the study by Kroslak et al. (Kroslak et al. J. Neurochem 2007 103:77) suggested that receptor internalization is a primary rate-limiting step for receptor resensitization and that enhanced resensitization confers apparent resistance to agonist-induced desensitization. Thus, consequences of altered N-linked glycosylation may be multifaceted and the fact that the internalized ligand β-Endorphine-TAMRA does not co-localize with the receptor MOPN40D-eGFP in trafficking vesicles may reflect such differences occurring via rapid recycling, an internalization mechanism that is dominated by transient formation of rapidly dissociating ligand-receptor complexes (Marchese et al. Annu. Rev. Pharmacol. Toxicol. 2008 48:601).

It is, however, also possible that β-Endorphin-TAMRA internalization without an obvious internalization of the receptor isoform MOPN40D-eGFP may partially occur through endogenous MOPwt. At present, we cannot assess the contribution of one process with respect to the other.
We have, however, observed similar results with hepta-enkephalin-TAMRA (Fig. S2G), where ligand-receptor complexes were occasionally detected, but the relative contribution of vesicles containing both signals was small compared to the number of TAMRA-containing vesicles (red).

We have discussed this extensively in the Supplementary Methods; subsection Validation of opioid receptor functionality by agonist treatment (pages 1 and 2).

6. What are the levels of the proteins in stably expressing PC-12 compared with other cell lines in S4?

While we cannot answer this question exactly, it is possible to make the following comparison. PC-PALM determined an average receptor density of the order of 40-50 detected molecules per μm2 in transiently transformed COS-7 cells, which was also shown to be in a physiologically relevant range (Fig. S3). As evident from the amplitudes of the tACC (Fig. 1D, Fig. 4B (green), Fig. 5A (green), Fig. S4B (middle)) and Fig. 1F (N1+N2), FCS detected about 4 – 30 molecules in the beam area, which can be determined according to the following equation: A = π· = 3.14·27×10-6·4·4.14×10-10 m2 = 1.4×10-13 m2 (Elson EL, Biophys. J. 2011 101:2855–2870). Assuming that the whole area is populated by opioid receptors, an estimated average opioid receptor density of 30-200 molecules per μm2 could be estimated. Thus, opioid receptor surface density was likely similar in both cell lines.

7. Everywhere in the text, use superscript for µm2.

We thank the Reviewer for spotting this style error, which we have now corrected throughout.

8. A few important references with regards to PALM have not been mentioned.
a) Annibale et al PloS One 2011
b) Nan et al PNAS 2013
c) Gunzenhäuser et al Nano Letters 2012
d) Sengupta et al Nature Proc 2013
e) Lee et al PNAS 2012

We have included these references in the revised manuscript, page 2.

Referee: 2

Comments to the Author
This paper uses FCS and PALM to estimate the lateral organization of KOR, MOR, and the MORN40D mutant. Based on time constants of autocorrelation curves and PALM data, the authors state a very broad model that receptors are organized in domains, are excluded from GM1 domains, and are organized in cholesterol domains.

It is difficult to put these findings in perspective, considering previously published data. The idea that cholesterol is involved in organization of membrane proteins including GPCRs such as opioid receptors is quite well established. While the argument that "quantitative and nondestructive methods are urgently needed to characterize under minimally invasive conditions the capacity of drug candidates to fine-tune receptor functions by harvesting this dynamic regulatory mechanism" in the context of allosteric modulators might be valid, this manuscript does not address this argument. There are a wide variety of agonists, antagonists, and allosteric modulators available, and if the authors can pick a few relevant ones and test opioid receptors by FCCS and PALM, this might be an opportunity to test a focused hypothesis and learn something new.

Alternatively, if the authors intend this as a methods paper, they need to be more rigorous in their controls.

We thank the Reviewer for this comment. We have provided in the revised manuscript control experiments for FCS and FCCS. Specifically, we have added Figures S4-S7 and performed the analysis, as detailed out in the Supplementary Methods. We have also extended our discussion on PALM and performed additional PALM and TIRF experiments (Figs 4D, 5B and S12).

A major concern is that receptors are tagged on the C-termini, this might cause them to behave differently from endogenous receptors. Most of the cells (e.g., Fig S2) show substantial ER fluorescence, which suggests that they are either overexpressed or are poorly folded. This is also compounded by the fact that MORN40D does not internalize, which suggests that the receptor might be altered. For example, KOR has a PDZ ligand on its C-terminus, and there is previous evidence that disrupting PDZ interactions can change diffusion kinetics of GPCRs (e.g., Valentine and Haggie, 2011).

We respectfully disagree with this comment. We have shown in the Supplementary Information, Figs S1-S3, that the opioid receptors are indeed functionally active in all cell lines used, and that neither ligand binding, nor downstream consequences of receptor activation (internalization and signaling) are impaired by the fluorophores. In the supplementary Figure S2, we show that KOP and MOPwt receptors in PC12 cells bind their corresponding ligands (Fig.S2 D and E, respectively); that PC12 cells internalize the ligand receptor complexes (Fig.S2 D and E, orange vesicles), traffic new KOP receptors to the cell surface (Fig. S2 D, green vesicles) and that the organic fluorophore TAMRA, which is not biodegradable, accumulates in the lysosomes (Fig. S2 D, red vesicles). In contrast to KOP and MOPwt, which show the canonical ligand-induced GPCR receptor internalization mechanism (Hanyaloglu AC, von Zastrow M. Annu Rev Pharmacol Toxicol. 2008 48:537), ligand internalization without massive receptor internalization was observed for MOPN40D using two specific ligands β-Endorphin-TAMRA (Fig. S2 F) and hepta-Enkephalin-TAMRA (Fig. S2 G). While this may partially be due to the presence of endogenous MOPwt, it may also be an indication of the rapid recycling internalization mechanism that is characterized by transient formation of rapidly dissociating ligand-receptor complexes (Marchese et al. Annu. Rev. Pharmacol. Toxicol. 2008 48:601). In the supplementary figure Fig. S3, we have shown that downstream activation of ERK1/2 and AKT is very similar for endogenous receptors and OR-

paGFP constructs. Please see also our response to Reviewer 1, point #5.

In addition to the discussion above, we provide here a paper by Scherrer G, Tryoen-Tóth P, Filliol D, Matifas A, Laustriat D, Cao YQ, Basbaum AI, Dierich A, Vonesh JL, Gavériaux-Ruff C, Kieffer BL. Knockin mice expressing fluorescent delta-opioid receptors uncover G protein-coupled receptor dynamics in vivo. Proc Natl Acad Sci U S A. 2006 103(25):9691-9696, where it was shown that: "C-terminal DOR-EGFP fusion shows unchanged binding, signaling, internalization, and down-regulation properties when expressed in HEK 293 cells (Table 1 and Movie 1, which are published as supporting information on the PNAS web site).".

As for the second comment: "Most of the cells (e.g., Fig S2) show substantial ER fluorescence, which suggests that they are either overexpressed or are poorly folded.", we again respectfully disagree with this remark. In unstimulated PC12 cells, Fig. S2 A-C, Fig. S1, Fig. 1B, majority of the receptors are located in the plasma membrane. In comparison, the signal from the cytoplasm, where these receptors are synthesized, folded, assembled and degraded, is rather feeble. The fact that the receptors are inserted in the plasma membrane already indicates that they are likely to be correctly folded and trafficked. The fact that they are internalized upon ligand-stimulation further strengthens this fact.

The goodness of fit should be shown for the curves individually. While the curve might fit best to three decay times, how much worse is it when fit to two phases or a single phase curve?
We thank the Reviewer for this comment. We have provided the requested information in Supplementary Information, Fig. S4. As can be seen from the residuals (Fig. S4C, bottom), the model with the smallest number of variables that could best represent our data was the model assuming two components diffusing in 2D and triplet correction.

The reason for ignoring the fast decay time is not explained well, especially as the data are not shown. What fraction of the total falls within this decay time? If they use a soluble eGFP, do they get just the fast decay time?
We thank the Reviewer for pointing out this. We have provided the requested data in the Supplementary Information, Fig. S5A, where we now show eGFP diffusion time measured in an independent experiment in the cytoplasm of live eGFP-expressing cells (violet), alongside KOP-eGFP tACC recorded at the apical plasma membrane of a live PC12 cell (dark green) and KOP-eGFP diffusion recorded at the membrane of a native vesicle spontaneously formed in the culture by cell swelling (light magenta). As can be seen, these tACC largely overlap at short time lags, $\tau <$ 200 μs, which is attributed to eGFP related photophysical/photochemical processes. At longer time lags, characteristic of diffusion-related processes, we observe pronounced differences. Most notably, while eGFP in the cytoplasm and KOP-eGFP in the membrane of the native vesicle show one characteristic diffusion-related decay time, KOP-eGFP recorded at the apical plasma membrane of live PC12 cells shows two.

In relation to his question, we also point out that the contribution of the component characterized by the shortest decay time can be seen from the normalized tACC shown in Fig. 1E, and we also indicate that the relative contribution of this component was < 20 % (page 8, left column, last line).

PC12 cells express endogenous opioid receptors, and also might secrete dynorphins and other opioid agonists, and it is not clear if these complicate the results.

We thank the Reviewer for this important comment. Autocrine signaling may indeed explain why two dynamically linked fractions of opioid receptors are observed. While we don't have at present conclusive experimental data on this topic, we plan to explore this idea in the future.
As to the question whether presence of endogenous receptors/ligands complicates the results, we would like to point out that while we track opioid receptors tagged with eGFP or paGFP, we don't expect that the presence of endogenous receptors/ligands would affect the lateral distribution of fluorescently labeled opioid receptors. Binding constants would be affected by the presence of endogenous opioid receptors and endogenous ligands, but we don't measure them here. We also point out that for distribution studies by PALM, we use COS-7 cells that do not have endogenous opioid receptors, per Western blots and RT-PCR (Supplemental Information, page 1 and Fig S3).

Summary of revisions made:

1. We have revised Fig. 1, where we have retained information on FCS measurement that was presented in old Fig. 1A-C, and transferred the data from Fig. 1D-F to the Supplementary Information because it is more related to the

interpretation of the FCS data. Thus, we have augmented Figure 1 with relevant complementary information about actual FCS measurements. The revised Figure 1 now shows: fluorescence intensity fluctuations recorded at the apical plasma membrane of a live PC12 cell stably transformed to express the fluorescently tagged wild type mu-opioid receptor (MOPwt-eGFP) (Fig. 1A); we have replaced our previous CLSM image showing a cluster of cells, which was regarded by Reviewer 1 (Minor comment 1) to be too bright, with an image of a representative low-level MOP-eGFP expressing PC12 cell (Fig. 1B); we show how the apical plasma membrane is located by using the so-called z-scan (Fig. 1C); we show an autocorrelation curve (tACC) obtained from the measurement on one cell (Fig. 1D); we show average tACC obtained from measurements on 8-10 cells (Fig. 1E); we show that the ratio of freely diffusing opioid receptor molecules and the number of molecules presumably associated with nano-domains is specific for each opioid receptor subtype (Fig. 1F).
2. Fig. 2 is previous Fig. 4
3. Fig. 3 is previous Fig. 5.
4. Fig. 4 is previous Fig. 2 augmented to include PC-PALM data on the effect of cholesterol depletion on opioid receptor lateral organization in COS-7 cells.
5. Fig. 5 is previous Fig 3 augmented to include opioid receptor-GM1 ganglioside co-localization studies in COS-7 cells by TIRF.
6. Fig. 6 is revised to better represent the model of lateral opioid receptor organization that emerges from our FCS and PC-PALM data. This interpretation is supported by Monte Carlo simulations.

We have also provided extensive new information in the Supplementary Methods. In particular, the following changes were made to existing figures and new figures were given. We have expanded Fig. S2 to include data on MOPN40D-eGFP stimulation with hepta-Enkephalin-TAMRA and added new figures S4-S8 and S12.

To the best of our knowledge, this is the first time the lateral organization of KOP, MOPwt, and MOPN40D has been characterized in such detail. Our data suggest that the complex dynamic behavior reflects the existence of freely diffusing receptor molecules that are in a dynamic equilibrium with a population of molecules that is transiently associated with cholesterol enriched domains. The size and the population density of the domain is receptor specific: the largest and most populated nano-domains were observed for KOP (~105 nm in radius; 9-10 detected molecules/domain), whereas the smallest and least populated domains were observed for MOPN40D (~82 nm in radius; 7-8 detected molecules/domain), with KOP showing the smallest and MOPN40D the largest fraction of receptors that reside outside of these domains. This finding may have profound implications – through this dynamic equilibrium the number of free vs associated MOPwt and KOP receptors, but not MOPN40D, may be swiftly regulated by controlling cholesterol levels in the plasma membrane, thereby affecting cellular signaling. We do agree that further studies are needed to confirm this dynamical sorting mechanism, demonstrate its relevance for receptor function and characterize how it is affected by specific ligands at opioid receptors and by non-specific substances such as ethanol. Ongoing studies in our laboratories are addressing these questions.

In the light of this information, we hope that you will find our manuscript to be sufficiently improved and suitable for publication in Traffic, and we thank you for your consideration.

Yours sincerely,

Vladana Vukojević and Tijana Jovanović-Talisman

Decision and Reviews

Dear Dr. Vukojevic

Thank you for submitting your revised manuscript "Lateral organization of opioid receptor variants (kappa, muwt and muN40D) in the plasma membrane at the nanoscale level" to Traffic. I asked the referees to read the revised paper and their verbatim comments are appended below. The referees are in agreement that the first round of revisions have addressed many of the concerns raised previously, but they also feel that a good deal more work is needed because your conclusions remain poorly supported by the data presented. Some concerns can be addressed by making changes to the text, but it is clear that additional data will be needed before the paper can be reconsidered. Both referees also have made numerous recommendations for correcting and improving the overall presentation, including adding data that are currently in supplemental materials to the main body of the manuscript.

It is Traffic policy to consider only one resubmission of a manuscript before it must be considered as a completely

new submission. I am happy to inform you that Traffic is willing to waive this policy in this case to provide you with a final opportunity to address the referee concerns. Should you wish to pursue publication in Traffic you will need to make every possible effort to address the referee concerns as detailed. I must caution you that Traffic will not be able to consider your manuscript further if the second revision does not completely and thoroughly address the attached critiques. To expedite handling when you resubmit please be sure to include a response outlining how you have addressed each of the referees' concerns.

Sincerely,

Trina A. Schroer, Ph.D.
Co-Editor

Referee's Comments to the Authors

Referee: 1

Comments to the Author
In their revised manuscript, Rogacki et al have addressed many concerns that I have had in the previous version. From a scientific perspective, I think the authors have done significant and meticulous work in showing that opioid receptors differ in their lateral organization. However, some of their conclusions need to be qualified. The presentation of the manuscript also needs to be substantially improved and the results of control experiments that are currently presented in the Supplementary Methods should be moved to the main text. At present, it is my opinion that an average reader would have difficulties in following the approaches used in manuscript.

Scientific Comments
1. In the abstract and later in the paper a claim is made that KOP forms the most populated domains whereas MOP_N40D forms the least populated domains. The numbers reported here are 9-10 versus 7-8 receptors, respectively. Are these numbers statistically different from one another?
2. The supposition that "the number of opioid receptors (N2) characterized by is a linear function of the number of opioid receptors (N1) characterized by the shorter diffusion time" may not be true for both the MOP variants. Visual inspection suggests the R^2 for the linear fit is quite large for the green dataset (MOP_wt). Because of this the authors should qualify this conclusion.
3. It is surprising that in figure 2F (and maybe in other figures too) the autocorrelation very clearly does not go to 1 at long length scales when it most likely should be random at such length scales. Do the authors think there are still large-scale structures at these long distance similar to those observed by Sengupta et al? One thing the authors could provide for better understanding is the values of all the parameters used in the fits as a table. With respect to this, have the authors considered the possibility that there are two sets of domain sizes in the PALM experiments?
4. The authors have now included a positive control for the association with GM1. Surprisingly, this positive control seems to indicate that the here is not much association between CTxB and Bodipy GM1. Could the authors offer an explanation for this? If the assay is not very sensitive to this positive control for GM1, it is possible that their conclusion that opioid receptors are excluded from GM1 domains is simply because of experimental limitations rather than biological effects.
5. In several places in the manuscript the authors indicate that the opioid receptors are localized in GPI-enriched domains (for example on page 5). Unless I am missing something, I did not see any evidence in the current manuscript supporting this claim. If it is based on previous work, it should be explicitly stated.

Comments on presentation
1. The abstract would be improved by starting it off with a sentence or two placing the biological problem under study in context.
2. The manuscript is not readily accessible for biologists who would presumably constitute a significant portion of the readers. The authors should talk a bit about the analysis (which is currently in the supplementary info) prior to discussing the results of the experiments in the main text itself. For instance, in the PALM experiments, prior to stating that PALM image analysis revealed the densities as it currently is, the authors could introduce the autocorrelation curves, talk briefly about the exponential and Gaussian fits, how densities are obtained from these fits and then refer to controls (figure s9) and then introduce the results. This would provide a more logical flow for the interested reader. In case of FCS, it would help in the presentation for the manuscript if the authors could provide a brief introduction to how analysis of FCS are generally performed, what autocorrelation curves mean and what information can be obtained from these curves.

3. A major concern is that the “supplementary methods” and “methods” section contain many pertinent results, especially controls. Most of these results are never mentioned in the main text, yet they are critical to document the approach used to generate the data shown here. To alleviate this problem the authors should move the majority of the descriptions of results into the main section of the text.
4. The results shown in Figure S7 are not described.
5. The authors have clearly performed substantial amounts of work indicated by a total of close to 20 figures. Given the importance and substantial number of many of the supplementary figures, it might be better to combine some of these into single figures and move figures showing key results into the main body of the text.
6. There is significant overlap in the description of the methods in the Supplementary Methods section and the main Methods section.

Minor Comments
1. GM1 should be written as GM1
2. In the PALM analysis, if I understand the analysis correctly, the authors determine the autocorrelation functions for areas in the 7-18 um^2 areas. This process is opaque as to how the authors choose these regions. Are these the entire regions of the cell or did the authors choose a portion of the cell for the analysis? If it is the latter, how was it achieved in an unbiased way?
3. In figure 2b, d, and f it is difficult to distinguish the two symbols. For instance, in figure 2F, it is unclear if the fits agree better with the simulation or experiment.
4. The labeling on the y-axis and legend for figure 3 need further clarification.
5. In figure 6, it would be helpful if different colors were used in different parts of the figure to ensure there is enough contrast for the reader.
6. The precision localization reported here seems quite high (+/- 1 nm). Is this the SD or SE?
7. The reference to Sengupta et al is repeated in the bibliography
8. Is there any reason why the authors have not talked about the PALM microscopy analysis for cholesterol depletion of MOP_N40D?
9. It will be very useful to reader if the authors put legends in all their figures. It is missing in some (ex. Figure 5).

Referee: 2

Comments to the Author
This paper uses FCS and PALM to study organization of opioid receptors on the plasma membrane. The conclusion that different opioid receptors are in different nanodomains and that cholesterol affects them differently, could be interesting. The authors have added some controls to minimize some of the original concerns, for example about endogenous receptors. However, I still have concerns about the data and interpretations.

- The authors seem to have misunderstood the comment on tagging receptors on the C-termini. The question is not whether C-terminal tags inhibit receptor signaling or general assays of internalization, often they do not. But tags can change receptor organization on the plasma membrane, e.g., directly shown in the paper on PDZ interactions (that KOP has) I referenced in the original review. The question of how much receptors are correctly folded and inserted to the plasma membrane and how much is in the ER (Fig S2) is at best arguable, and cannot be used as an argument that the receptors are fine.

One control is to repeat a key experiment (decay times?) with an N-terminally tagged receptor and show that they are comparable. While it is possible that C-terminal tags do not affect receptor distributions in this case, published data suggest a high likelihood that they might, and it would not be in the authors’ best interests to publish a manuscript with this caveat.

- The interpretations of some of the data are still confusing. The authors conclude that “Opioid receptors partially associate with cholesterol-enriched domains” based on data in Fig 4A (yellow cross correlation line). Then they conclude the opposite, that “Opioid receptors are largely excluded from GM1 ganglioside-enriched domains” based on what seems to this reviewer an identical cross correlation in 5B between GM1 and receptors. Why are the interpretations so different?

In S12, it is not clear why the conclusion is that there is limited colocalization. In the cells that show both labels, there seems to be very good colocalization to this reviewer.

The same images are used in both Fig S12 and 5B.

- The authors conclude that MOPN40D has a higher domain density (about 33%) compared to average density, but they also state that it has a lower average density by about 20% - "PALM image analysis revealed that MOPwt and KOP surface densities are comparable, with (52 ± 4) and (51 ± 4) detected molecules/µm2 on average, respectively. In contrast, MOPN40D density was lower, with (43 ± 3) detected molecules/µm2 (P value < 0.03)." Is the difference in the domain density ratio simply because of a change in average density?

The PALM data also suggests that the MOPN40D is expressed at a lower level than the others. Could this influence the differences they see? The authors need to discuss this.

The authors state "significantly different" without statistical analysis, e.g. in 4B. The authors need to do statistical analysis of their curves and include the significance in the main manuscript.

As previously noted, I think authors need to be more thorough in their discussion of published literature and be careful in their claims. There have been several studies of GPCRs at single-molecule resolution, including, for example, a recent study by Halls et al., Sci Signal. 2016. PMID: 26861044, which studied MOPwt organization in plasma membrane nanodomains. Similarly, the Beyer et al. paper referenced (as part of a group of 18 papers), shows that N40D is internalized, which conflicts with this report. I encourage the authors to be more comprehensive in their discussion of relevant published data.

It is not clear why the authors have preferred to put the large part of their data in the supplemental figures, the main figures are small. Some of the supplemental analysis and data are important, and could be moved into the main manuscript.

Author Rebuttal

Dear Dr. Schroer,

Thank you for considering our manuscript "Lateral organization of opioid receptor variants (kappa, muwt and muN40D) in the plasma membrane at the nanoscale level" (TRA-16-0483) for publication in Traffic. We are grateful to the Reviewers for reading our work and for their constructive criticism. We also appreciate the opportunity to revise our writing. Following Reviewer suggestions, we have thoroughly revised our manuscript to address raised concerns. We hope that you will find our manuscript to be significantly improved and suitable for publication in Traffic.

On behalf of all Authors,

Vladana Vukojević and Tijana Jovanović-Talisman

Responses to Reviewer Comments

Reviewer: 1

Comments to the Author
In their revised manuscript, Rogacki et al have addressed many concerns that I have had in the previous version. From a scientific perspective, I think the authors have done significant and meticulous work in showing that opioid receptors differ in their lateral organization. However, some of their conclusions need to be qualified. The presentation of the manuscript also needs to be substantially improved and the results of control experiments that are currently presented in the Supplementary Methods should be moved to the main text. At present, it is my opinion that an average reader would have difficulties in following the approaches used in manuscript.
We thank the Reviewer for these important and valuable insights that have allowed us to improve the manuscript. We now provide a description of the methodology in the introduction, with a new Fig. 1. Additionally, we have included some data from Supplemental figures in the main text.

Scientific Comments

1. In the abstract and later in the paper, a claim is made that KOP forms the most populated domains whereas MOP_N40D forms the least populated domains. The numbers reported here are 9-10 versus 7-8 receptors, respectively. Are these numbers statistically different from one another?
We thank both Reviewers for raising this important question. To address this, we compare correlation curves using the F-test to identify differences among sample variance and standard errors of the estimate (S-values). Our results are summarized in Table S2. We show that variances of two populations are equal to within 5 % between experimental and best-fit simulated curves (parameters reported in Table 1). We also compared (1) different experimental curves, and (2) experimental MOPwt curves with an extensive set of simulated curves (number of receptors in domains was changed to 9-10 or 7-8; radius of domain was changed to 79 nm or 101 nm; the distribution pattern of receptors was changed). For all conditions when using the F-test, variances of the two populations are not equal and thus, the null hypothesis is rejected."

2. The supposition that "the number of opioid receptors (N2) characterized by is a linear function of the number of opioid receptors (N1) characterized by the shorter diffusion time" may not be true for both the MOP variants. Visual inspection suggests the R^2 for the linear fit is quite large for the green dataset (MOP_wt). Because of this the authors should qualify this conclusion.
We have performed additional measurements on MOPwt expressing PC12 cells in order to improve the statistics (Fig. 2F in the revised version). Original data were retained and newly measured data points were added. While small changes in the slope were observed, these differences were within the experimental error of the measurement. As can be seen, the general trend was retained and the overall conclusions were not altered.

3. It is surprising that in figure 2F (and maybe in other figures too) the autocorrelation very clearly does not go to 1 at long length scales when it most likely should be random at such length scales. Do the authors think there are still large-scale structures at these long distance similar to those observed by Sengupta et al? One thing the authors could provide for better understanding is the values of all the parameters used in the fits as a table. With respect to this, have the authors considered the possibility that there are two sets of domain sizes in the PALM experiments?
Long-range correlations (beyond 300 nm) were not observed in any of our correlation curves (Figs 3B, D, F in the revised version) and no evidence of larger-scale structures is seen in any case. In all cases reported here, autocorrelation functions approach a value close to 1 at longer distances. X-asymptote fitting of the autocorrelation function (from the pair-correlation method originally published in Sengupta et al., 2011 is allowed to vary as 1 ± 0.5. The set of parameters used for fitting is now clearly described in Materials and Methods: Fitting spatial autocorrelation curves (sACCs).
To address the possibility that we have different domain sizes (in particular, a combination of smaller and larger domains), we ran an extensive set of simulations and a subset is shown in Fig. S9D. In all tested simulated cases, we did not obtain curves that matched experimental curves. Although we cannot completely exclude alternative types of molecular organization, the set of tested conditions and variables from our data are consistent with organized nanodomains.

4. The authors have now included a positive control for the association with GM1. Surprisingly, this positive control seems to indicate that there is not much association between CTxB and Bodipy-GM1. Could the authors offer an explanation for this? If the assay is not very sensitive to this positive control for GM1, it is possible that their conclusion that opioid receptors are excluded from GM1 domains is simply because of experimental limitations rather than biological effects.
While CTxB is often used as a probe to detect GM1 ganglioside because of its high affinity for this glycosphingolipid in vitro, a number of studies on live cells have shown that CTxB binding does not necessarily correlate with GM1 levels (e.g. Yanagisawa et al. Glycobiology 2006 16:19G–23G). A possible explanation for this phenomenon was recently provided using complex glycolipid mixtures on supported lipid bilayers (Krishnan et al. Colloids Surf B Biointerfaces 2017 160:281-288. doi: 10.1016/j.colsurfb.2017.09.035), where it was shown that dissociation constants cannot exclusively represent multivalent CTxB bindings and that binding cooperativity also plays an essential role in determining CTxB-cell membrane recognition. In accordance with this notion, our finding by FCCS is not surprising. While this assay is not as specific in live cells as it is in vitro, we still believe that our conclusion that opioid receptors are largely excluded from GM1 domains is not an artifact that simply arises because of experimental limitations rather than biological effects. To begin with, we have shown in control experiments with stably transformed PC12 cells expressing MOPwt-Tomato (red) treated with BODIPY FL C5-ganglioside GM1 that a similar result, i.e. limited co-localization and cross-correlation, is obtained (Fig. S7). Secondly, we clearly observe by CLSM (Fig. 5A) and TIRF (Fig. 5D) that cells which express low levels of opioid receptors are readily stained by CTxB, whereas cells that express opioid receptors are generally not well stained by CTxB.

Having said this, we agree with the Reviewer that this limitation needs to be discussed. We have therefore transferred data from the SI to the main text (Fig. 5B, b1-b3), we have determined the equilibrium dissociation constant for the complex formed between fluorescently labeled CTxB-AF647 and BODIPY® FL C5-ganglioside GM1 in solution, Kd = $(2 \pm 1)\times10$-6 M, and have elaborated the writing in order to better convey our reasoning.
5. In several places in the manuscript the authors indicate that the opioid receptors are localized in GPI-enriched domains (for example on page 5). Unless I am missing something, I did not see any evidence in the current manuscript supporting this claim. If it is based on previous work, it should be explicitly stated.
We apologize for this oversight and we now properly cite our previously published work by Tobin et al., 2014 at appropriate places.

Comments on presentation

1. The abstract would be improved by starting it off with a sentence or two placing the biological problem under study in context.
We thank the reviewer for the suggestion. The abstract has been rewritten.

2. The manuscript is not readily accessible for biologists who would presumably constitute a significant portion of the readers. The authors should talk a bit about the analysis (which is currently in the supplementary info) prior to discussing the results of the experiments in the main text itself. For instance, in the PALM experiments, prior to stating that PALM image analysis revealed the densities as it currently is, the authors could introduce the autocorrelation curves, talk briefly about the exponential and Gaussian fits, how densities are obtained from these fits and then refer to controls (figure s9) and then introduce the results. This would provide a more logical flow for the interested reader. In case of FCS, it would help in the presentation for the manuscript if the authors could provide a brief introduction to how analysis of FCS are generally performed, what autocorrelation curves mean and what information can be obtained from these curves.
Methodological details are now included per Reviewer's suggestion.

3. A major concern is that the "supplementary methods" and "methods" section contain many pertinent results, especially controls. Most of these results are never mentioned in the main text, yet they are critical to document the approach used to generate the data shown here. To alleviate this problem the authors should move the majority of the descriptions of results into the main section of the text.
We agree that this is more appropriate, and have moved descriptions of results into the main section of the text. We have, retained in the supplement information that relate to instrument calibration, optical setting validation and control numerical simulations.

4. The results shown in Figure S7 are not described.
We have corrected this.

5. The authors have clearly performed substantial amounts of work indicated by a total of close to 20 figures. Given the importance and substantial number of many of the supplementary figures, it might be better to combine some of these into single figures and move figures showing key results into the main body of the text.
We have now revised all figures and included a portion of the material from the Supplementary Information to the main text.

6. There is significant overlap in the description of the methods in the Supplementary Methods section and the main Methods section.
The overlap is now significantly reduced and only additional information is included in SI.

Minor Comments
1. GM1 should be written as GM1
This is now corrected.

2. In the PALM analysis, if I understand the analysis correctly, the authors determine the autocorrelation functions for areas in the 7-18 um^2 areas. This process is opaque as to how the authors choose these regions. Are these the entire regions of the cell or did the authors choose a portion of the cell for the analysis? If it is the latter, how was it achieved in an unbiased way?
Areas are chosen randomly. Only flat regions are included to avoid artifacts from cell regions that do not completely

adhere to coverslips. The protocol follows that applied in Sengupta et al., 2011 and is now clarified in the text.

3. In figure 2b, d, and f it is difficult to distinguish the two symbols. For instance, in figure 2F, it is unclear if the fits agree better with the simulation or experiment.
We have changed the shading to clarify the image. The results are now presented in Figs 3B,D,F.

4. The labeling on the y-axis and legend for figure 3 need further clarification.
The labeling and legend for Fig 3 (Fig. 3G in the revised version) are further clarified.

5. In figure 6, it would be helpful if different colors were used in different parts of the figure to ensure there is enough contrast for the reader.
We have increased the contrast in the image to improve visibility.

6. The precision localization reported here seems quite high (+/- 1 nm). Is this the SD or SE?
We now clarify this as standard deviation. Standard errors are approximately 0.3 nm.

7. The reference to Sengupta et al is repeated in the bibliography 8. Is there any reason why the authors have not talked about the PALM microscopy analysis for cholesterol depletion of MOP_N40D?
Thank you for pointing this out, duplicate references are now removed. The MORN40D experiment has not been performed due to time constrains (months of work). We have included only MOR and KOR. This is now clarified in the text.

8. It will be very useful to reader if the authors put legends in all their figures. It is missing in some (ex. Figure 5).
We have corrected this.

Reviewer: 2

Comments to the Author
This paper uses FCS and PALM to study organization of opioid receptors on the plasma membrane. The conclusion that different opioid receptors are in different nanodomains and that cholesterol affects them differently, could be interesting. The authors have added some controls to minimize some of the original concerns, for example about endogenous receptors. However, I still have concerns about the data and interpretations.

The authors seem to have misunderstood the comment on tagging receptors on the C-termini. The question is not whether C-terminal tags inhibit receptor signaling or general assays of internalization, often they do not. But tags can change receptor organization on the plasma membrane, e.g., directly shown in the paper on PDZ interactions (that KOP has) I referenced in the original review. The question of how much receptors are correctly folded and inserted to the plasma membrane and how much is in the ER (Fig S2) is at best arguable, and cannot be used as an argument that the receptors are fine. One control is to repeat a key experiment (decay times?) with an N-terminally tagged receptor and show that they are comparable. While it is possible that C-terminal tags do not affect receptor distributions in this case, published data suggest a high likelihood that they might, and it would not be in the authors' best interests to publish a manuscript with this caveat.
Valentine and Haggie (Valentine and Haggie, 2011) make several important points about scaffolding interactions between the C-terminal motifs in β1- and β2- adrenergic receptors (AR) and their partners, the PDZ-domain and AKA proteins. They conclude that specific interactions with the C-terminal tail of ARs are central for receptor tethering, as the reviewer mentioned. However, it has been shown by von Zastrow and colleagues (Gage et al. J Biol Chem. 2001 276(48):44712-20) that: "the β2-adrenergic receptor contains an autonomous, transplantable endocytic sorting signal, which is sufficient to re-route a heterologous GPCR (delta-opioid receptor (DOP) was used) into a rapid recycling pathway and to cause functionally significant changes in agonist-induced down-regulation of receptors". Thus, a specific sequence, which is naturally present in the C-terminal domain of β2- adrenergic receptor, is needed to re-route the innate trafficking of DOP. Moreover, it was shown that the sequence required for this protein interaction is not conserved in most other GPCRs, that the cytoplasmic tail of mu-opioid receptor (MOP) contains a sequence that does not bind detectably neither to known PDZ proteins nor to the N-ethylmaleimide-sensitive factor (NSF), and that the carboxyl-terminal sequence present in KOP does not correspond to a consensus PDZ ligand (Gage et al. J Biol Chem. 2005 280(5):3305-13 and references therein). While the Reviewer raises an important question, this question is particularly relevant for β-adrenergic receptors, and the same extent of importance was not established for opioid receptors and other GPCRs. We would also like to underline that our opioid receptor constructs contain the full-

length C-terminal tail, a short linker, and eGFP/paGFP. As such, C-terminal motifs can remain available for physiologically relevant interactions. Finally, we would like to point out that over the last 2 decades, many researchers demonstrated that C-terminal GFP tags do not alter GPCR functionality and GPCR-GFP constructs have been regularly employed to study receptor trafficking, localization, and agonist/antagonist interactions (Barak et al., 1997; Tarasova et al., 1997; Sarramegna et al., 2002; McLaughlin et al., 2003; Perret et al., 2003; McLaughlin et al., 2004; Wang et al., 2005; Bruchas et al., 2006; Carrel et al., 2008; Vukojevic et al., 2008a; Vukojevic et al., 2008b; Kieffer and Evans, 2009; Manzke et al., 2010; Poole et al., 2011; Liebmann et al., 2012; Erbs et al., 2015). Further evidence using super resolution microscopy suggests that minimal changes occurred in the clustering behaviors of the serotonin receptor upon addition of a C-terminal fluorescent tag (Vreja et al., 2015). Furthermore, in the context of a MOP-eGFP construct, it has been shown that the fusion protein can insert into the plasma membrane similar to MOPwt, remain sensitive to its respective ligands, and couple with trafficking machinery (Vukojevic et al., 2008a; Vukojevic et al., 2008b). Similar to this characterization, further references for MOP (Wang et al., 2005; and for KOP (McLaughlin et al., 2003; McLaughlin et al., 2004; Wang et al., 2005; Bruchas et al., 2006) have used C-terminal GFP tagged proteins, some with single molecule methods. The overwhelming evidence in the literature, in addition to functional studies that have been presented, indicate that our constructs are well suited for the study.

- The interpretations of some of the data are still confusing. The authors conclude that “Opioid receptors partially associate with cholesterol-enriched domains” based on data in Fig 4A (yellow cross correlation line). Then they conclude the opposite, that “Opioid receptors are largely excluded from GM1 ganglioside-enriched domains” based on what seems to this reviewer an identical cross correlation in 5B between GM1 and receptors. Why are the interpretations so different?
We have revised the text in order to better convey the information. In Fig. 4A, we show using CLSM imaging that co-localization between the fluorescently labeled cholesterol analog cholesteryl-BODIPY (red) and MOPwt-eGFP (green) is observed in some regions of the cellular plasma membrane. This is seen from the overlap of the red and green signals that give rise to a clearly visible yellow signal, but not in other regions where we interchangeably see red or green signal. In Fig. 4B we show that there is no cross-correlation between these signals. Based on this, and the fact that the lateral dynamics and organization of opioid receptors is affected by plasma membrane cholesterol depletion, we have concluded that (1) opioid receptors sort into some cholesterol-enriched domains, but not into other cholesterol-enriched domains which may be enriched with other proteins; and (2) we conclude that the cholesteryl-BODIPY probe is largely unbound to opioid receptors.
For the CTxB experiments, where red-labeled CTxB is used to visualize GM1 (and other gangliosides), we most often observe clearly separated CTxB- and opioid receptor-related fluorescence signals. In fact, they appear to be mutually exclusive and are most often observed in different cells – cells expressing low levels of opioid receptors readily bind CTxB, which confirms that the probe works reliably, whereas cells expressing opioid receptors at a moderate-high level do not bind CTxB efficiently. And, when occasionally observed in the same cell, the fluorescence signals from the distinct fluorophores are most often clearly spatially separated (Fig. 5). We also show by FCCS that these signals do not cross-correlate. We therefore conclude that opioid receptors are largely excluded from GM1 (and other ganglioside-containing domains).
However, we agree with the Reviewer that this is an important question. We have therefore thoroughly revised this section. In particular, we have transferred data from the SI to the main text (Fig. 5B, b1-b3), we have determined the equilibrium dissociation constant for the complex formed between fluorescently labeled CTxB-AF647 and BODIPY® FL C5-ganglioside GM1 in solution, Kd = $(2 \pm 1) \times 10$-6 M, and have elaborated the writing in order to better convey our reasoning.
Please see also our response to Reviewer 1, point 4 in Scientific Comments.

In S12, it is not clear why the conclusion is that there is limited colocalization. In the cells that show both labels, there seems to be very good colocalization to this reviewer. The same images are used in both Fig S12 and 5B.
We now provide quantification of co-localization to support our conclusion. We provide separate channels of images, and also show a summary for all cells in Fig 5D. Old Fig. S12 is now removed.

- The authors conclude that MOPN40D has a higher domain density (about 33%) compared to average density, but they also state that it has a lower average density by about 20% - “PALM image analysis revealed that MOPwt and KOP surface densities are comparable, with (52 ± 4) and (51 ± 4) detected molecules/µm2 on average, respectively. In contrast, MOPN40D density was lower, with (43 ± 3) detected molecules/µm2 (P value < 0.03).” Is the difference in the domain density ratio simply because of a change in average density?
We now include additional clarification. Increased local density in domains represents the ratio of the density in a domain to the total cell density. While the Reviewer is completely correct that lower total density can influence this

number, the domain density is also a factor.

The PALM data also suggests that the MOPN40D is expressed at a lower level than the others. Could this influence the differences they see? The authors need to discuss this.
Lower MOPN40D compared to MOPwt density observed with PALM is congruent with Western blots and extensive literature data. While some GPCRs show density dependence (Rocheville et al., 2000; Hern et al., 2010; Kasai et al., 2011; Calebiro et al., 2013), others show a similar distribution over a wide range of expression levels (Herrick-Davis et al., 2013). Thus, the differences in density can influence organization in certain cases, where other factors (such as the loss of a glycosylation site) can be a driving force for organization changes. This is now included in the discussion and references pointing to lower density of MOPN40D are included. Additionally, it is worth noting that MOP and KOP have similar density, but different organization.

The authors state "significantly different" without statistical analysis, e.g. in 4B. The authors need to do statistical analysis of their curves and include the significance in the main manuscript.
Statistical analysis is now included in Table S2 and results are discussed in the main text. Please also see our response the first comment from reviewer 1.

As previously noted, I think authors need to be more thorough in their discussion of published literature and be careful in their claims. There have been several studies of GPCRs at single-molecule resolution, including, for example, a recent study by Halls et al., Sci Signal. 2016. PMID: 26861044, which studied MOPwt organization in plasma membrane nanodomains. Similarly, the Beyer et al. paper referenced (as part of a group of 18 papers), shows that N40D is internalized, which conflicts with this report. I encourage the authors to be more comprehensive in their discussion of relevant published data.
We have expounded the introduction to refer to important previous work where single-molecule approaches were used to study opioid receptor lateral dynamics and organization in the plasma membrane, and have highlighted the most important findings in the study by Halls et al. Sci. Signal. 2016.

It is not clear why the authors have preferred to put the large part of their data in the supplemental figures, the main figures are small. Some of the supplemental analysis and data are important, and could be moved into the main manuscript.
We have moved some figures and results from supplementary to the main text.

____

References

Barak, L.S., Ferguson, S.S., Zhang, J., Martenson, C., Meyer, T., and Caron, M.G. (1997). Internal trafficking and surface mobility of a functionally intact beta2-adrenergic receptor-green fluorescent protein conjugate. Molecular pharmacology 51, 177-184.
Bruchas, M.R., Macey, T.A., Lowe, J.D., and Chavkin, C. (2006). Kappa opioid receptor activation of p38 MAPK is GRK3- and arrestin-dependent in neurons and astrocytes. The Journal of biological chemistry 281, 18081-18089.
Calebiro, D., Rieken, F., Wagner, J., Sungkaworn, T., Zabel, U., Borzi, A., Cocucci, E., Zurn, A., and Lohse, M.J. (2013). Single-molecule analysis of fluorescently labeled G-protein-coupled receptors reveals complexes with distinct dynamics and organization. Proc Natl Acad Sci U S A 110, 743-748.
Carrel, D., Masson, J., Al Awabdh, S., Capra, C.B., Lenkei, Z., Hamon, M., Emerit, M.B., and Darmon, M. (2008). Targeting of the 5-HT1A serotonin receptor to neuronal dendrites is mediated by Yif1B. The Journal of neuroscience : the official journal of the Society for Neuroscience 28, 8063-8073.
Erbs, E., Faget, L., Scherrer, G., Matifas, A., Filliol, D., Vonesch, J.L., Koch, M., Kessler, P., Hentsch, D., Birling, M.C., Koutsourakis, M., Vasseur, L., Veinante, P., Kieffer, B.L., and Massotte, D. (2015). A mu-delta opioid receptor brain atlas reveals neuronal co-occurrence in subcortical networks. Brain structure & function 220, 677-702.
Hern, J.A., Baig, A.H., Mashanov, G.I., Birdsall, B., Corrie, J.E., Lazareno, S., Molloy, J.E., and Birdsall, N.J. (2010). Formation and dissociation of M1 muscarinic receptor dimers seen by total internal reflection fluorescence imaging of single molecules. Proc Natl Acad Sci U S A 107, 2693-2698.
Herrick-Davis, K., Grinde, E., Cowan, A., and Mazurkiewicz, J.E. (2013). Fluorescence correlation spectroscopy analysis of serotonin, adrenergic, muscarinic, and dopamine receptor dimerization: the oligomer number puzzle. Mol Pharmacol 84, 630-642.
Kasai, R.S., Suzuki, K.G., Prossnitz, E.R., Koyama-Honda, I., Nakada, C., Fujiwara, T.K., and Kusumi, A. (2011). Full characterization of GPCR monomer-dimer dynamic equilibrium by single molecule imaging. J Cell Biol 192, 463-480.
Kieffer, B.L., and Evans, C.J. (2009). Opioid receptors: from binding sites to visible molecules in vivo. Neuropharmacology 56 Suppl 1, 205-212.

Liebmann, T., Kruusmagi, M., Sourial-Bassillious, N., Bondar, A., Svenningsson, P., Flajolet, M., Greengard, P., Scott, L., Brismar, H., and Aperia, A. (2012). A noncanonical postsynaptic transport route for a GPCR belonging to the serotonin receptor family. The Journal of neuroscience : the official journal of the Society for Neuroscience 32, 17998-18008.
Manzke, T., Niebert, M., Koch, U.R., Caley, A., Vogelgesang, S., Hulsmann, S., Ponimaskin, E., Muller, U., Smart, T.G., Harvey, R.J., and Richter, D.W. (2010). Serotonin receptor 1A-modulated phosphorylation of glycine receptor alpha3 controls breathing in mice. The Journal of clinical investigation 120, 4118-4128.
McLaughlin, J.P., Myers, L.C., Zarek, P.E., Caron, M.G., Lefkowitz, R.J., Czyzyk, T.A., Pintar, J.E., and Chavkin, C. (2004). Prolonged kappa opioid receptor phosphorylation mediated by G-protein receptor kinase underlies sustained analgesic tolerance. The Journal of biological chemistry 279, 1810-1818.
McLaughlin, J.P., Xu, M., Mackie, K., and Chavkin, C. (2003). Phosphorylation of a carboxyl-terminal serine within the kappa-opioid receptor produces desensitization and internalization. The Journal of biological chemistry 278, 34631-34640.
Perret, B.G., Wagner, R., Lecat, S., Brillet, K., Rabut, G., Bucher, B., and Pattus, F. (2003). Expression of EGFP-amino-tagged human mu opioid receptor in Drosophila Schneider 2 cells: a potential expression system for large-scale production of G-protein coupled receptors. Protein expression and purification 31, 123-132.
Poole, D.P., Pelayo, J.C., Scherrer, G., Evans, C.J., Kieffer, B.L., and Bunnett, N.W. (2011). Localization and regulation of fluorescently labeled delta opioid receptor, expressed in enteric neurons of mice. Gastroenterology 141, 982-991 e981-988.
Rocheville, M., Lange, D.C., Kumar, U., Patel, S.C., Patel, R.C., and Patel, Y.C. (2000). Receptors for dopamine and somatostatin: Formation of hetero-oligomers with enhanced functional activity. Science 288, 154-157.
Sarramegna, V., Talmont, F., Seree de Roch, M., Milon, A., and Demange, P. (2002). Green fluorescent protein as a reporter of human mu-opioid receptor overexpression and localization in the methylotrophic yeast Pichia pastoris. Journal of biotechnology 99, 23-39.
Sengupta, P., Jovanovic-Talisman, T., Skoko, D., Renz, M., Veatch, S.L., and Lippincott-Schwartz, J. (2011). Probing protein heterogeneity in the plasma membrane using PALM and pair correlation analysis. Nat. Methods 8, 969-975.
Tarasova, N.I., Stauber, R.H., Choi, J.K., Hudson, E.A., Czerwinski, G., Miller, J.L., Pavlakis, G.N., Michejda, C.J., and Wank, S.A. (1997). Visualization of G protein-coupled receptor trafficking with the aid of the green fluorescent protein. Endocytosis and recycling of cholecystokinin receptor type A. The Journal of biological chemistry 272, 14817-14824.
Valentine, C.D., and Haggie, P.M. (2011). Confinement of beta(1)- and beta(2)-adrenergic receptors in the plasma membrane of cardiomyocyte-like H9c2 cells is mediated by selective interactions with PDZ domain and A-kinase anchoring proteins but not caveolae. Mol Biol Cell 22, 2970-2982.
Vreja, I.C., Nikić, I., Göttfert, F., Bates, M., Kröhnert, K., Outeiro, T.F., Hell, S.W., Lemke, E.A., and Rizzoli, S.O. (2015). Super-resolution Microscopy of Clickable Amino Acids Reveals the Effects of Fluorescent Protein Tagging on Protein Assemblies. ACS Nano 9, 11034-11041.
Vukojevic, V., Ming, Y., D'Addario, C., Hansen, M., Langel, U., Schulz, R., Johansson, B., Rigler, R., and Terenius, L. (2008a). Mu-opioid receptor activation in live cells. FASEB journal : official publication of the Federation of American Societies for Experimental Biology 22, 3537-3548.
Vukojevic, V., Ming, Y., D'Addario, C., Rigler, R., Johansson, B., and Terenius, L. (2008b). Ethanol/naltrexone interactions at the mu-opioid receptor. CLSM/FCS study in live cells. PloS one 3, e4008.
Wang, D., Sun, X., Bohn, L.M., and Sadee, W. (2005). Opioid receptor homo- and heterodimerization in living cells by quantitative bioluminescence resonance energy transfer. Mol Pharmacol 67, 2173-2184.

Decision and Reviews

Dear Dr. Vukojević,

Thank you for submitting your revised manuscript “Dynamic lateral organization of opioid receptor variants (kappa, muwt and muN40D) in the plasma membrane at the nanoscale level” to Traffic. I asked the referees to read the revised paper and their verbatim comments are appended below. I am a bit dismayed that both referees still have concerns, some of which remain significant. Both recommend extending and clarifying the discussion. Referee 1 makes suggestions to improve the presentation of some of the data. Very importantly, referee 2 raises a very significant concern about the potential complications resulting from C-terminal tagging of the receptor. I am in complete agreement that this concern must be addressed experimentally if it is your goal to have this study published in Traffic. Furthermore, it is essential that you do a better job of completely citing the published literature to include work that may not be fully compatible with your conclusions.

As you know, it is Traffic policy to consider only one resubmission of a manuscript before it must be considered as a completely new submission. Moving forward at this point will involve a third resubmission. Traffic will only be able to consider another version that addresses the referee concerns completely via additional experimentation and proper treatment of the scientific literature as it pertains to your work. This will be your final opportunity to perform essential controls and to provide a fully balanced treatment of the literature.

To expedite handling when you resubmit, please include a response detailing how you have addressed each of the referees' concerns and the changes you have made to the manuscript.

Sincerely,

Trina A. Schroer, Ph.D.
Co-Editor

Referee's Comments to the Authors

Referee: 1

Comments to the Author
The authors have addressed most of the previous round of comments. The current version of the manuscript is improved and more accessible as a result. I have several minor comments on the current version that the authors should consider prior to publication of this work.

1) While the authors report differences in the behavior of the different opioid receptors, there is very little or no discussion of why this might be the case except for the glycosylation mutant. The biological implications of this study would be strengthened if they were to include some appropriately labeled speculation on this issue as part of the Discussion.
2) In response to the comments about the low cross-correlation between BODIPY-GM1 and CTxB, the authors included a paragraph in the results discussing evidence that they bind. This paragraph seems out of place and could be moved to the supplementary material section.
3) On a related note, a recent paper (Rissanen et al, 2017, Front Physiol) discussed some of the complications with the use of BODIPY GM1 and CTxB. It would be worth mentioning this paper as part of the discussion of the limitations of this study.
4) The authors claim to have made adjustments to Figure 6, but it is still very difficult to distinguish between the different types of structures depicted in this cartoon. Using colors with higher contrast to differentiate between the different types of domains would be helpful.

Referee: 2

Comments to the Author
In this revised manuscript, the authors have performed additional simulations and data analysis, and have unfortunately argued against most concerns. The manuscript is still very dense and fragmented, and it is hard to read and appreciate it. This also makes it difficult to assess if the changes made address the concerns raised previously.

- I still believe the C-terminal tag is a serious issue that is the source of some controversy in the field. The authors' repeated reluctance to perform a straightforward and easy experiment (tag the receptor on either end and show that there is no difference in a key assay) to resolve this confusion, even after two requests, is disappointing. The authors cite work by von Zastrow and colleagues, that adrenergic receptors have a transplantable PDZ-binding sorting signal, to argue that opioid receptors are different. The von Zastrow group has shown that mu opioid receptors also have a transplantable sorting signal on the C-terminal tail, which when transplanted to the delta opioid receptor can re-route the receptor (Tanowitz, 2003), just like the adrenergic receptors. The kappa opioid receptor can bind NHERF1, a PDZ protein, through the C-terminus (e.g., Li et al., 2002). Tagging the delta opioid receptors on the C-terminus can change its localization (Wang et al., 2008). All these have been ignored by the authors.

- The conclusions are often drawn without statistical tests - for example, Fig 5B, 5D. The authors have included a table S2 for statistics, but it is not clear which test relates to which result. The authors have to show results from relevant

statistical tests on each graph on each figure from which they draw a conclusion.

- The discussion of previous literature needs to be thorough beyond the one or two papers that the reviewers have brought to their attention. I will repeat the previous concern that the Beyer et al. paper, which suggests that N40D is internalized, is referenced, but not in this context, which is important for their results. Some of the literature discussion is in the Supplemental methods section, which will not be read by most readers.

Author Rebuttal

Dear Dr. Schroer,

We sincerely appreciate the opportunity to resubmit our manuscript (TRA-16-0483) to Traffic. We thank you and the Reviewers on the thorough feedback and constructive criticism. The manuscript has been strengthened by the insightful comments and suggestions that we have received during the reviewing process and the writing is significantly improved as a result. As suggested by the Reviewers, we have added more citations, updated our discussion section, and we have also presented a more comprehensive survey of the literature. Most importantly, we have also performed the control experiment with N-terminally tagged opioid receptors that were requested. We show the obtained results, which are in agreement with our previous results with C-terminally tagged opioid receptors, in the Supplementary Methods Fig. S11. An account of changes made and detailed responses to the Reviewers' requests are enclosed below.

We also kindly ask to slightly change the title of our work. We propose to omit the word "variants" from the title and suggest a new title for our revised manuscript: "Lateral organization of opioid receptors (kappa, muwt and muN40D) in the plasma membrane at the nanoscale level". We are motivated to propose this change because it is in line with text editing that we have made. If this is not possible, due to technical or other reasons, we will retain our original title.

We hope that you will find the revised manuscript to be significantly improved and suitable for publication in Traffic, and we thank you for your time and for your consideration.

Best regards,
Vladana Vukojevic and Tijana Jovanovic-Talisman

Decision and Reviews

Dear Dr. Vukojević,

Thank you for submitting your revised manuscript "Dynamic lateral organization of opioid receptor variants (kappa, muwt and muN40D) in the plasma membrane at the nanoscale level" to Traffic. I asked the referees to read the revised paper and their verbatim comments are appended below. I agree with the referees that you have addressed the concerns raised previously. Referee 1 recommends acceptance at this time. Referee 2 recommends revision of the text to include a more careful review of the previous literature that I agree would strengthen this paper.

With this minor revision I would be pleased to accept this paper for publication.

Sincerely,

Trina A. Schroer, Ph.D.
Co-Editor

Referee's Comments to the Authors

Referee: 1

Comments to the Author
The authors have made additional revisions to the manuscript that have further improved it. I support its publication

in its current form.

Referee: 2

Comments to the Author
The authors have addressed the concerns appropriately within the scope of the paper. Similar experiments have been done by others (e.g., Halls et al., 2016), these can be better discussed and directly compared, considering that there is overlap in experiments and conclusions.

Author Rebuttal

Dear Dr. Schroer,

We were pleased to hear that our manuscript (TRA-16-0483) was provisionally accepted for publication in Traffic, pending minor revision.
As suggested by the Referees:

Referee: 1
The authors have made additional revisions to the manuscript that have further improved it. I support its publication in its current form.

Referee: 2
The authors have addressed the concerns appropriately within the scope of the paper. Similar experiments have been done by others (e.g., Halls et al., 2016), these can be better discussed and directly compared, considering that there is overlap in experiments and conclusions.

we have revised the last paragraph on page 9 in the Discussion in order to provide a more detailed description of the results by Halls et al. 2016 and relate them to our findings. The revised paragraph reads: "Irrespective of how the lateral organization of opioid receptors (or other GPCRs) is brought about and maintained in live cells, enrichment of plasma membrane proteins in multi-protein assemblies may be a fundamental principle with important functional implications for lateral transfer of information in the plasma membrane and for signal transduction across the plasma membrane.46,52,102-105 Recently, Halls et al.52 have shown that MOPwt lateral organization is subject to distinct changes upon stimulation with specific agonists. They have also shown that lateral organization is a relevant determinant of MOPwt function, as it gives rise to distinct ligand-induced spatiotemporal signaling profiles.52 Our observation that lateral organization of MOPwt is dynamic in unstimulated cells is in line with the finding by Halls et al.52. Importantly, our results suggest that other investigated opioid receptors also have dynamic and distinct lateral organization in unstimulated cells…"

We have also written a synopsis for the TOC and have made a figure for the graphical abstract, as requested by the Managing Editor.

We hope that you will find the revised manuscript to be suitable for publication in Traffic, and we thank you for your time and for your consideration.

Best regards,
Vladana Vukojevic and Tijana Jovanovic-Talisman
